# Genomic organization and recombinational unit duplication-driven evolution of ovine and bovine T cell receptor gamma loci

**DOI:** 10.1186/1471-2164-9-81

**Published:** 2008-02-18

**Authors:** Giovanna Vaccarelli, Maria C Miccoli, Rachele Antonacci, Graziano Pesole, Salvatrice Ciccarese

**Affiliations:** 1 Department of Genetics and Microbiology, University of Bari, via Amendola 165/A, 70126 Bari, Italy; 2 Department of Biochemistry and Molecular Biology "Ernesto Quagliariello", University of Bari, via Orabona 4, 70125 Bari, Italy

## Abstract

**Background:**

In humans and mice ("γδ low species") less than 5% of the peripheral blood T lymphocytes are gamma/delta T cells, whereas in chicken and artiodactyls ("γδ high species") gamma/delta T cells represent about half of the T cells in peripheral blood. In cattle and sheep (Bovidae) two paralogous T cell receptor gamma loci (TRG1 and TRG2) have been found. TRG1 is located on 4q3.1, within a region of homology with the human TRG locus on chromosome 7, while TRG2 localizes on 4q2.2 and appears to be unique to ruminants. The purpose of this study was the sequencing of the genomic regions encompassing both loci in a "γδ high" organism and the analysis of their evolutionary history.

**Results:**

We obtained the contiguous genomic sequences of the complete sheep TRG1 and TRG2 loci gene repertoire and we performed cattle/sheep sequence analysis comparison using data available through public databases. Dot plot similarity matrix comparing the two sheep loci with each other has shown that variable (V), joining (J) and constant (C) genes have evolved through a series of duplication events involving either entire cassettes, each containing the basic V-J-J-C recombinational unit, or single V genes. The phylogenetic behaviour of the eight enhancer-like elements found in the sheep, compared with the single copy present in the human TRG locus, and evidence from concordant insertions of repetitive elements in all analyzed TRGJ blocks allowed us to infer an evolutionary scenario which highlights the genetic "flexibility" of this region and the duplication-driven evolution of gene cassettes. The strong similarity of the human and Bovidae intergenic J-J-C regions, which display an enhancer-like element at their 3' ends, further supports their key role in duplications.

**Conclusion:**

We propose that only duplications of entire J-J-C regions that possessed an enhancer-like element at their 3' end, and acquired at least one V segment at their 5' end, were selected and fixed as functional recombinational units.

## Background

In vertebrates cellular immunity is based on antigen recognition by αβ or γδ T cell receptors (TR) chains. Although γδ T cells share many functions and features with αβ T cells, they are unique because of their thymic development, their antigen recognition (they recognize soluble protein and non protein antigens without MHC restriction) [1,2], and their tissue distribution. In general γδ T cells show an intermediate phenotype between innate and adaptive immunity, and exhibit a pronounced immunoregulatory function [3]. T cell receptor variable domains are assembled during lymphocyte development from V, D, and J segments by V(D)J recombination. In developing T cells, specific molecular mechanisms target the recombinase activity to appropriate TR loci according to the allelic exclusion rule. This recombinase targeting at the level of the substrate locus, is known as recombinational accessibility [4-6]. The enhancer elements are involved in controlling the accessibility since they govern the general accessibility of each locus in V(D)J recombination. Deletion of the enhancers abolishes rearrangements in the TRB locus and greatly reduces rearrangements in the TRA locus [7]. In TRA/TRD and TRB loci a tightly correlation has been demonstrated between histone acetylation and V(D)J recombination and a mechanism for coupling enhancer activity with accessibility has been proposed [8,9].

The percentage of γδ T cells is variable among species. Sheep and cattle, as well as swine [10], and chicken [11], are considered "γδ high" species. In young ruminants the circulating γδ T cells account for more than 50% of circulating blood cells, but this percentage decreases to 5% by adulthood [12]. On the other hand, in "γδ low" species, like humans and mice, although the proportion of γδ T cells in the blood decreases with age, even before adulthood their percentage is consistently lower than in "γδ high" species. Moreover for both "γδ low and high" species γδ T cells can represent up to 50% of the lymphocytes in epithelium-rich tissues, such as the skin, the gut and the reproductive tract [1].

While γδ T cells from humans and mice have been extensively studied in their function, antigen responsiveness and structural organization of the genomic loci coding for the γ and δ chains, less is known in γδ high species. Bovine γδ T cells are able to respond to nonpeptidic molecules derived from Mycobacteria, including isopentenylpyrophosphate (IPP) [13]. Whatever the role of γδ T cells in cellular immunity the mechanisms, which led to a significant increase of these cells in young ruminants and chickens, are still unknown. In sheep, the TRG locus has a double chromosomal localization on the 4q3.1 (TRG1) and 4q2.2 (TRG2) chromosome bands; the locus is localized on the homologous bands in cattle, goat and river buffalo. Only TRG1 is found within a region of the *Ovis aries *genome which is syntenic with the human region containing the TRG locus on chromosome 7, while the TRG2 locus appears to be unique to ruminants [14,15]. The organisation of loci in their basic structure was determined, each locus consisting of three cassettes composed by one variable (V), two joining (J) and one constant (C) genes lying in the same transcriptional orientation [16,17].

In humans and mice the structure of the TRG locus is peculiar when compared to the other T cell receptor loci, since only a few genes encode γ chains [18-20], while a repertoire of more than 50 genes, distributed in long arrays of V and/or J genes, encodes α and β chains [20].

Moreover, in the TRG locus a considerably higher number of C genes is found in ruminants, than in other mammalian species: for example, there are only two TRGC genes in human [18-20] and four, of which one is a pseudogene, in mouse [21]. It has been proposed [2] that different TRGC genes, each possessing unique connecting regions [22], could mediate different cellular responses after the T cell receptor has engaged the antigen. In cattle the expression of specific TRGC genes may be restricted to specific lymphocyte T populations [23,24].

In this study our aims were: i) to determine the overall genomic arrangement of the sheep TRG1 and TRG2 loci by assembling their contiguous genomic sequences; ii) to outline the sequence of events that gave rise to the current structural organisation of the two TRG loci. Closer inspection of the cassettes, each containing the basic V-J-J-C unit, using evidence from DNA sequence relationships and repetitive elements insertion allowed us to propose a model highlighting the cassette duplication-driven evolution in Bovidae.

## Results and Discussion

### Overall arrangement of TRG1 and TRG2 loci in sheep

T cell receptor gene families exhibit tremendous genetic diversity, not only in the number of genes, but also in the rearrangements used to create protein coding sequences [19,20]. In previous papers we described the entire ovine repertoire of the variable (V), joining (J) and constant (C) ovine genes and their organization in the TRG1 and TRG2 loci. The isolation of five TRG1 BAC clones and two TRG2 BAC clones, their sub-cloning in plasmid vectors and the sequencing of their inserts allowed us to obtain contiguous genomic sequences spanning 158.8 Kb for TRG1 [available in GEDI for GenBank/EMBL/DDBJ and IMGT/LIGM-DB: DQ992075] and 95.0 Kb for TRG2 [available in GEDI for GenBank/EMBL/DDBJ and IMGT/LIGM-DB: DQ992074].

In the γδ high Bovidae lineage, as pointed out by recent phylogenetic results on cattle and sheep variable genes, the complex TRG1 locus, containing the ancestral V-J-C cassette, contrasts with the more recent TRG2 locus presumably originated by a translocation event [17]. Furthermore, an enhanced expression deriving from a coordinated expansion of TRDV1 subgroup genes of the variable δ repertoire was recently discovered in adult sheep [25].

The availability of the complete sequence of the ovine TRG loci allowed us to attempt a reconstruction of the succession of events that shaped their current genomic organization starting from the most likely ancestral locus, and led to the rapid expansion of their copy number. We first analysed the compositional properties (G + C content) of the sequences obtained and identified tandem and interspersed repeated sequences. The GC content of 40.6% is almost identical for the two ovine clusters and is consistent with the general observation that SINE-rich regions have a high GC content.

The Tandem Repeats Finder program identified 26 different tandem repeats in the TRG1 locus and 14 in TRG2. Repeats vary in monomer length from 2 to 142 bp for TRG1 and from 2 to 46 bp for TRG2, and in copy number from 1.9 to 23.5 for TRG1 and from 2 to 28.5 for TRG2 (See Additional file 1, Table S1a, b).

With regard to the interspersed repeated elements, the analysis carried out through the Repeat Masker program provided the results summarized in additional Tables a (TRG1) and b (TRG2) (See Additional file 2, Table S2a, b) and presented in detail in additional Tables c and d (See Additional file 3, Table S3c, d). The density of total repeats both in TRG1 (30.39%) and in TRG2 (32.59%), is slightly lower than the density reported for the TRG loci of man and mouse [19]. SINEs are the most abundant elements in the ovine loci as in their human (16.32%) and murine counterpart (14.83%).

**Figure 1:**
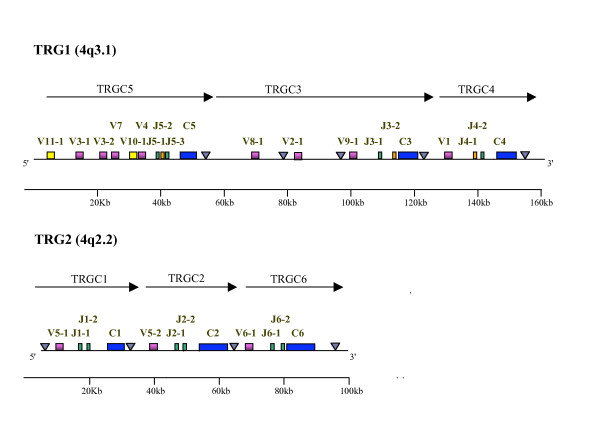
**Schematic representation of the TRG1 and TRG2 loci**. Diagram of the general arrangement of the TRG1 and TRG2 ovine loci. The diagram shows the position of all V, J and C TRG genes. Boxes representing genes are not to scale. Exons are not shown. Yellow box: V pseudogene; violet box: V functional; green box: J functional; orange box: J pseudogene; blue box: C. Arrow heads indicate the position of the Enhancer-like sequences. Arrows indicate the transcriptional orientation of TRGC cassettes.

Fig. 1 shows the overall organization of the ovine TRG1 and TRG2 loci. It should be noted that according to an IMGT suggestion (correspondence with MP Lefranc) as an upgrade to the nomenclature used in previous reports, in the present report, we will use the term "cassette" instead of "cluster" when referring to the "V-J-J-C" units belonging to the sheep and bovine TRG loci. Fig. 1 shows the entire TRG1 locus, which encompasses three cassettes, TRGC5, TRGC3 and TRGC4, named according to the constant genes [16,17,26-29] and following the recommendations of the IMGT Nomenclature Committee. The limit dividing the three cassettes from each other was set at approximately 4–5 Kb downstream of the last exon of each C gene. Similarly Fig. 1 shows the entire TRG2 locus, consisting of TRGC1, TRGC2 and TRGC6 cassettes. All TRG cassettes lie in the same transcriptional orientation and are closely spaced. In each "V-J-J-C" cassette, the V gene and a J gene rearrange and, after transcription, the V-J is then spliced to the relevant C in mature transcripts [17,26]. The only exception is the TRGC1, where the variable V5-1 is rearranged with J1-1 and/or J1-2, both spliced to C2 [17]. We detected by RT-PCR the transcripts for all genes belonging to TRGC5 [17], including the TRGV10-1 gene [29], which contains a premature stop codon in exon 2 [17]. Furthermore we identified the V11-1 gene, never before described, which was deposited [GenBank: DQ992075]. V11-1 belongs to the TRGC5 5' region, presents 79% nucleotidic similarity with the functional human counterpart TRGV9 [GenBank: NG_001336], has a premature stop codon and has never been found transcribed. All C-proximal (Jp) and C-distal (Jd) J genes have been found transcribed in adult and foetus with the only exception of the J5-2, located in the middle [27]. J3-2, wrongly previously reported as a vestigial form [17], presents the FVNGIKF motif and a premature stop codon at 3' end of coding region. With regard to the C genes, it should be noted that EX1, corresponding to the disulfide-linked constant domain, and EX3, corresponding to the transmembrane and cytoplasmatic domains, are similar in length in all genes. Conversely, the EX2A, EX2B and EX2C exons, which encode the connecting region, differ between C genes both in number and length (Fig. 1). The only peculiar feature being C1 gene, where the presence of only the first three codons in EX2B has been demonstrated [28].

### Comparison of TRG1 and TRG2 loci in Bovidae

**Figure 2:**
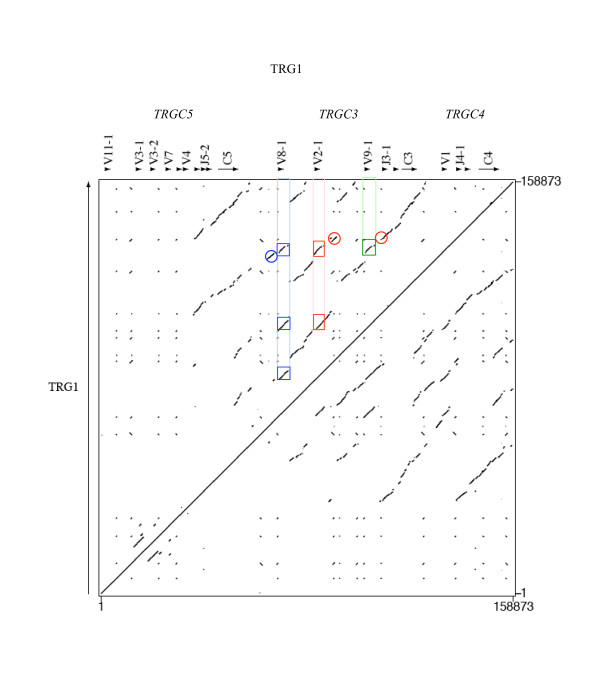
**Dotplot matrix of ovine TRG1 genomic comparison**. Using the PipMaker program ovine TRG1 has been plotted against itself. The transcriptional orientation of each gene is indicated by arrows and arrow-heads. Coloured rectangles enclose identical regions including V8-1, V2-1, V9-1 and V1. Blue circle indicates that V1 shows in its upstream position a short similarity region shared with V8-1. Red circles indicate that the 3' region of V1 compared with the corresponding V9-1 and V2-1 regions features 80% and 74.9% identity, respectively.

**Figure 3:**
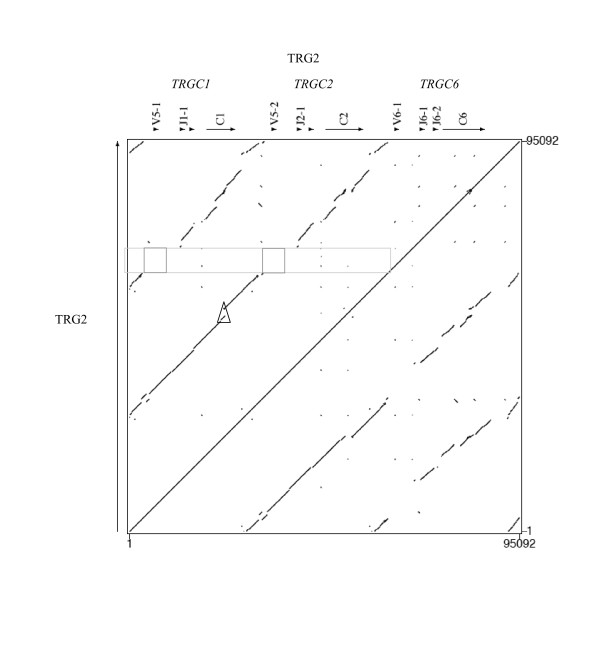
**Dotplot matrix of ovine TRG2 genomic comparison**. Using the PipMaker program ovine TRG2 has been plotted against itself. The transcriptional orientation of each gene is indicated by arrows and arrow-heads. The ovine C1 internal deletion is shown in a triangle. Squares indicate absence of similarity.

Figs. 2 and 3 show the dotplot matrixes comparing the sequence of each ovine locus against itself. As expected for loci consisting of a series of clusters of correlated genes, the matrixes show numerous homologous regions of various sizes. The most continuous block of similarity exists between the J-C regions of the TRG cassettes and the longest similarity block starting from V and ending in C regions is found at the diagonal concerning TRGC2 vs. TRGC1 in Figure 3. In Figure 2 the TRGC3 vs. TRGC4 similarity extends to the V regions while the 5' region of the TRGC5 cassette, characterized by the presence of six V genes, does not show similarity with any other region of the entire locus, except partially with itself. This is also true for the single V gene (V6-1) belonging to TRGC6 cassette in the TRG2 locus.

**Figure 4:**
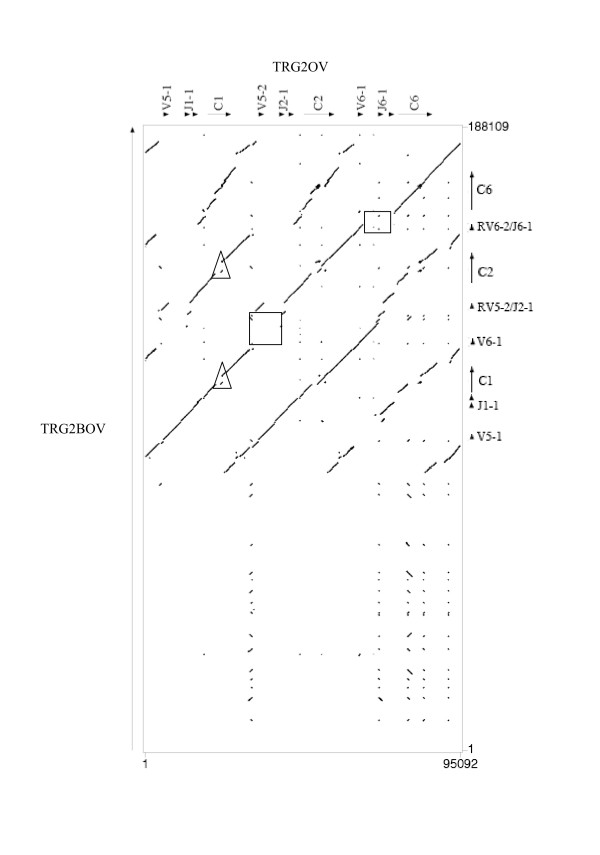
**Dotplot matrix of ovine TRG2/bovine TRG2 genomic comparison**. Using the PipMaker program ovine TRG2 has been plotted against bovine TRG2. The transcriptional orientation of each gene is indicated by arrows and arrow-heads. The ovine C1 internal deletion is shown in a triangle. Squares indicate absence of similarity.

All the genes and intergenic regions of the TRGC2 and TRGC1 blocks, feature high identity levels (Table 1); the region of similarity is largely uninterrupted and the duplicated blocks appear superimposed "end to end". Thus the duplication that originated the TRGC2 and TRGC1 units is likely to have occurred very recently. Duplication events in the genomic regions containing variable genes only occurred in the TRG1 locus, giving origin to three (TRGV8-1, TRGV2-1, TRGV9-1) and 6 (TRGV11-1, TRGV3-1, TRGV3-2, TRGV7, TRGV10-1, TRGV4,) genes in the TRGC3 and TRGC5 cassettes respectively (Fig. 2). We have compared cattle contiguous sequences [AY644517] (See Additional file 4, Figure S4), [NW_937068] (not shown) and [AY644518] (Fig. 4) [29] vs. the sheep TRG1 and vs. the sheep TRG2 loci. Considering TRG1, there are 7 genes in the TRGC3 cassette in bovine but only 3 in ovine. The TRG2 sequences from cattle and sheep are superimposable with the following exception: a) there is a deletion of exons 2B and 2C in ovine TRGC1 (triangles in Fig. 4); b) there are two sequence gaps in the intergenic region that is one between TRGV5-2 and TRGJ2-1 and another one from the TRGV6-2 exonic region to TRGJ6-1 (squares) due to two TRGV-J rearrangements observed in the bovine TRG2 (See Additional file 5, Figure S5) [29]; c) there are two TRGV6 genes in bovine but only one in ovine.

**Table 1:** Percentage^a ^of nucleotide sequence identity in pairwise alignments of genomic regions including TRG V8-1, V2-1, V9-1, V1, V5-1 and V5-2 genes

	**V8-1**	**V2-1**	**V9-1**	**V1**	**V5-1**	**V5-2**
**V8-1**	-	3206 bp	4036 bp	2151 bp	2319 bp	2376 bp
		78.4%	78.3%	77.4%	77.0%	77.0%
**V2-1**		-	5227 bp	2825 bp	3627 bp	4833 bp
			78.0%	78.0%	77.0%	77.9%
**V9-1**			-	2184 bp	3016 bp	3063 bp
				78.8%	76.8%	76.8%
**V1**				-	2580 bp	2796 bp
					79.8%	79.4%
**V5-1**						4863 bp
					-	97.9%
**V5-2**						-

^a^The percentages have been calculated as weighted average of identity values obtained by blastz algorithm (PipMaker) on the conserved regions.

**Figure 5:**
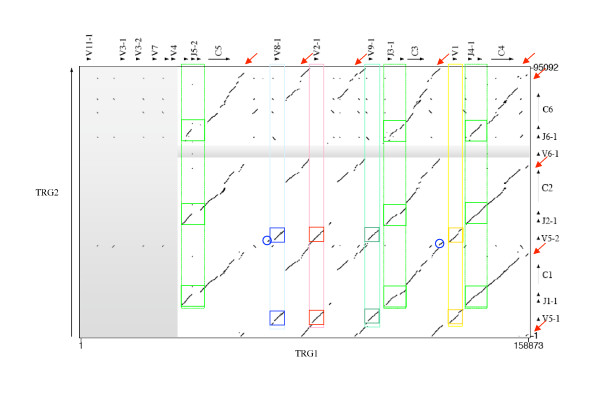
**Dotplot matrix of ovine TRG1/TRG2 genomic comparison**. Transcriptional orientation of genes is indicated by arrows and arrow-heads. Coloured rectangles include identical regions in the alignment of V8-1 (blue), V2-1 (red), V9-1 (dark green) and V1 (yellow) with V5-1 and V5-2 genes while grey areas represent absence of identity and are discussed in the text. Blue circles indicate that V8-1 and V1 show in their upstream position a short similarity region shared with V5-2. Light green rectangles highlight the identical regions between the V and C genes of each cassette (TRGJ blocks). Red arrows indicate the position of the enhancer-like sequences in ovine TRG loci.

Fig. 5 shows the dotplot matrix comparing the sequences of TRG1 vs. TRG2 ovine loci. The conservation of the J-J-C region of each cassette, starting from the distal J gene and ending at 3' of the C gene, is quite straightforward. Also the absence of similarity diagonals is evident for the V genes of the TRGC5 cassette vs. all other Vs of the TRG2 locus and for the single TRGV6-1 gene of TRGC6 cassette vs. all the other V of the TRG1 locus. Instead in the comparison of TRGC3 and TRGC4 cassettes vs. TRGC2 and TRGC1 cassettes the conservation of similarity is extended to the whole block from the 5' region of the TRGV9-1 gene up to 3' of the C4 gene. Therefore the TRGV5-1 and TRGV5-2 genes seem to be equally correlated to TRGV8-1, TRGV2-1, TRGV9-1 and TRGV1 genes of the TRG1 locus. The identity percentages between similarity regions, ranging in size from 2.1 to 5.2 Kb and containing variable genes, are shown in Table 1. The analysis of the matrix gives an idea of the duplicative mechanisms, which appear to have involved either the entire V-J-J-C unit, as in the TRG2 locus or single V genes (V2-1, V8-1, V9-1) as in the TRGC3 cassette of TRG1 locus. The relationship between the V genes belonging to TRGC3 and those belonging to the remaining cassettes will be discussed further below. It must be observed that the region at 5' of the TRGV5-1 gene and at 3' of C1, C2 and C6 genes of the TRG2 locus share high identity percentage with the regions located at 3' of C4, C3 and C5 genes and with the region at 5' of TRGV2-1 and TRGV9-1 genes of the TRG1 locus.

**Figure 6:**
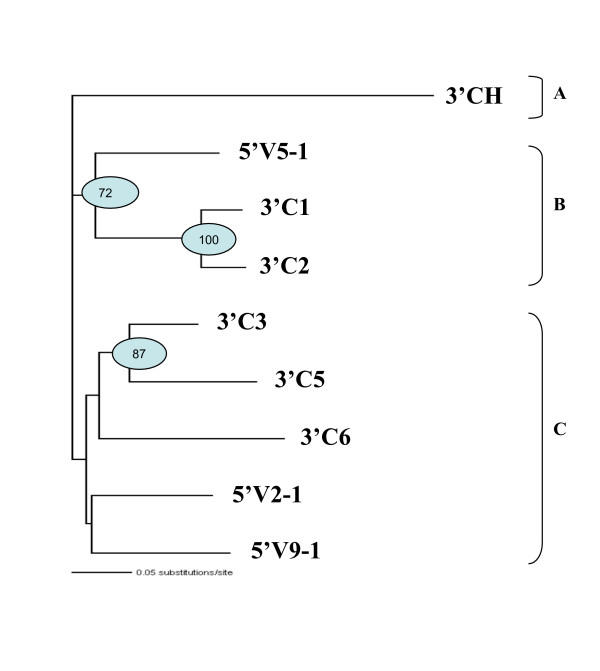
**Phylogram tree of the TRG loci enhancer-like sequences**. The enhancer-like sequences present in the ovine TRG loci were identified analyzing the regions of similarity between the human enhancer region (position 140573–141360 of the TRG locus contig [GenBank: NG_001336] [GenBank: AC006033]) and the entire ovine TRG loci by using the PipMaker program. Human (3'CH) and ovine (3'C5, 3'C3, 3'C1, 3'C2, 3'C6, 5'V5-1, 5'V2-1, 5'V9-1) enhancer-like sequences were multialigned. The phylogenetic tree has been constructed on 506 ungapped sites of the enhancer alignment by using the PAUP* software with the Neighbor Joining method on GTR distances. Significant bootstrap values (>50%) over 1,000 replicates are also shown.

Fig. 5 highlights in each locus the conservation in similarity and in length of the intergenic regions inclusive of the C gene of a cassette and the V gene of the next cassette, the only difference lying in the C5 – V8-1 region that is rich in repeated elements, which are the main cause of its peculiar behaviour in the matrix. These C-V intergenic regions are characterized by the presence of a sequence similar to the only single copy of the TRG locus enhancer localized at 3' of the human C2 gene [30] (See accession number and positions in the sequence for the human TRG enhancer sequence in Methods). From now on this sequence will be referred to as the enhancer-like sequence. In the TRG2 locus the enhancer-like sequence is present in four copies distributed either immediately downstream of the C6, C2 and C1 genes or immediately upstream of TRGV5-1. The TRG1 locus contains five copies of the enhancer-like sequence, localized immediately downstream of the C5, C3 and C4 genes or immediately upstream of TRGV2-1 and TRGV9-1. Fig. 6 shows a phylogram obtained from the alignment of a common region about 900 bp long of eight of the nine enhancer-like sequences analyzed. The division of the phylogram in three parts (A, B and C) is useful to understand the evolutionary relationship of the analyzed sequences. The upper part (A) features the human enhancer element. The middle part (B) features a cluster with including the sheep enhancer-like sequences located at the 5' end of TRGV5-1 (TRGC1) and the sequences at the 3' end of C2 and C1 constant genes, which derive from the most recent duplications. The lower part (C) refers to a cluster containing both the sheep enhancer-like sequences at the 3' end of the oldest C5, C6 and C3 and at 5' end of TRGV2-1 (TRGC3) and TRGV9-1 (TRGC3).

### Relationships between the variable genes of TRG1 and TRG2 loci

The total lack of similarity of the V genes of the TRGC5 cassette and the single V gene of the TRGC6 cassette with all the remaining Vs (Figs. 2, 3, and 5) suggests their more ancient origin, in accord with the phylogenetic results obtained, which highlighted the existence of an ancient TRGC5 cassette in cattle and sheep [16]. Human/mouse/Bovidae comparison data presented in the last subsection of Results and Discussion support this "evolutionary freezing" which appears to have only concerned the genomic regions containing the V genes involved in antigen recognition, excluding them from the subsequent duplications of Bovidae TRG loci. On the other hand, similarities were found between V genes belonging to the TRGC3, TRGC4, TRGC2 and TRGC1 cassettes. We used several features of the dot plot to help deduce the nature of the duplication events that occurred.

The areas of high similarity related to the TRGV8-1, TRGV2-1, TRGV9-1 and TRGV1 genes (Figs. 2 and 5), stretching from 5' to 3' ends (average of 6–12 Kb), may help define the most probable duplicative events that occurred in the TRGC3 V region. In particular:

i) it is clear that TRGV2-1 and TRGV9-1 originated through an extended duplication that mostly concerned their 5', where the similarity region appears a little fragmented in the matrix.

ii) TRGV1 shows in its upstream position a short similarity region shared with TRGV8-1, but absent in TRGV2-1 and TRGV9-1 (blue circle in Fig. 2); the same upstream region is present, though less expanded, in TRGV8-1 and TRGV1 vs. TRGV5-2, but is lost in TRGV8-1 and TRGV1 vs. TRGV5-1 (blue circle in Fig. 5).

Considering the succession of evolutive events proposed in previous reports [16] (according to which the cassette TRGC3 is more recent than TRGC5, but older than TRGC4) and the similarities highlighted in the previous point ii, we can hypothesize that the duplication of the entire TRGC3 cassette (V8-1, J3-1, J3-2, C3) originated TRGC4 (V1, J4-1, J4-2, C4). This hypothesis is in agreement with the data on the distribution of the repeated elements shown in Table 2, since TRGV8-1 shares with TRGV1 six out of nine elements in 5'V.

**Table 2:** Repeats shared by two or more conserved blocks corresponding to rows 1–14: V8-1, V2-1, V9-1, V1, V5-1 and V5-2 intergenic regions and rows 15–47: cassette-specific J-C intergenic regions

**No**	**Type^a^**	**Position^b^**	**V8-1**	**V2-1**	**V9-1**	**V1**	**V5-1**	**V5-2**
***1***	***Sine BovtA2***	5' V	X			X(A2)	X(tA3)	X
***2***	***Sine MIR***	5' V	X(b)	X		X(b)	X	X(3)
***3***	***LTR MLT1H (C)***	5' V	X			X		
***4***	***LTR MLT1H (C)***	5' V	X			X		
***5***	***Line L1MD2***	5' V	X			X		
***6***	***Line L1MD2***	5' V		X		X	X	X
***7***	***Sine BovtA2***	5' V	X			X		
***8***	***GA-rich***	5' V	X	X		X	X	X
***9***	***Line BovB***	5' V	X		X		X	X
***10***	***Line L1M2 (C)***	5' V	X(MA8)	X(MA9)	X(MA9)	X	X	X
***11***	***MER5A***	3' V	X	X	X		X	X
***12***	***Sine BovtA2 (C)***	3' V		X		X	X	X
***13***	***Line L1M2***	3' V				X	X	X
***14***	***Sine MIR***	3' V		X	X			

			**TRGC4**	**TRGC2**	**TRGC3**	**TRGC6**	**TRGC5**	**TRGC1**
			
***15***	***Sine MIRb (C)***	5' Jd			X		X	
***16***	***AT rich***	5' Jd		X			X	X
***17***	***Line L1M5 (C)***	5' Jd	X(MC)	X	X(MD)			X
***18***	***Sine BovtA3***	5' Jd		X			X(tA2)	X
***19***	***Line L2 (C)***	3'Jd	X	X	X	X	X	X
***20***	***Sine MIRb (C)***	3'Jd	X	X	X	X	X	X
***21***	***Sine BovtA2***	3'Jd	X		X			
***22***	***Line L4 (C)***	3'Jd	X	X	X	X		X
***23***	***Sine BovA2***	3'Jd				X(tA3)	X	
***24***	***AT rich***	3'Jd				X	X	
***25***	***Sine MIRb***	3'Jp	X	X	X		X	X
***26***	***Sine BovtA2***	3'Jp		X				X
***27***	***CHR-L***	3'Jp	X(2B)	X				X
***28***	***Line L1 Art (C)***	3'Jp	X					X
***29***	***CHR-L***	5'C		X				X
***30***	***Sine BovtA3***	5'C		X			X	X
***31***	***Sine MIRb***	5'C	X	X(m)	X	X	X	X
***32***	***Sine BovtA2***	I intr. C				X	X	
***33***	***Sine MIRb***	I intr. C	X	X	X	X	X	X
***34***	***Line L4 (C)***	I intr. C	X	X		X	X	X
***35***	***Sine BovtA2***	I intr. C		X	X	X		X
***36***	***Sine CHR-2 (C)***	I intr. C	X		X(B)	X(B)		
***37***	***Sine BovA2***	I intr. C		X			X	
***38***	***Sine MIRb***	I intr. C	X	X	X	X	X	X
***39***	***Line L2 (C)***	I intr. C	X				X	
***40***	***Sine MIRm (C)***	I intr. C		X				X
***41***	***Sine BovtA1***	II intr.C			X	X		
***42***	***Sine MIRb (C)***	ult.intr.C	X MARS	X		X(m)	X	X
***43***	***Sine MIR***	ult.intr.C	X	X			X(b)	X(b)
***44***	***Sine BovA2***	ult.intr.C		X	X(tA2)	X(tA2)		X
***45***	***Sine MIR***	ult.intr.C		X	X	X		X
***46***	***Sine MIR***	3'C	X	X	X	X	X(m)	X
***47***	***Sine MIR (C)***	3'C			X	X	X(b)	

^a ^Type of repeat as reported in RepeatMasker output file. (C) indicates the opposite orientation of the repeat. ^b ^Repeat position. Jd e Jp signify respectively the distal or the proximal J segments with respect to the C gene of each cassette. The cassette-specific J-C intergenic regions include the sequence spanning from 5' of distal J segment and 3' of C gene (exon 3) for each TRGC cassette.

In consideration of point i) and of data in Fig. 6, we can therefore hypothesize that TRGV2-1 (or TRGV9-1) was initially generated by the duplication of a genomic region including the 3' end of C3 gene, the TRGV1 gene and a small portion at its 3' end, while the other gene is assumed to have originated subsequently by duplication of a region including the newly formed one. It is not possible to unequivocally establish the order of events that originated the TRGV2-1 and TRGV9-1 genes, whether TRGV2-1, TRGV9-1 or TRGV9-1, TRGV2-1. Moreover, the identity data (from comparison of regions of about 2.2 Kb around and inclusive of TRGV1 vs. the corresponding regions of TRGV8-1, TRGV2-1 and TRGV9-1) indicates a higher resemblance of TRGV1 with TRGV9-1 than with TRGV8-1 or TRGV2-1 (Table 1). In addition the 3' region of TRGV1 compared to TRGV9-1 features 80% identity, which is higher than the identity with the corresponding TRGV2-1 region (74.9%) (red circles in Fig. 2). As mentioned above, the comparison of identity percentages presented (Tab. 1) does not highlight a significantly higher identity for any gene pair, the only exceptions being TRGV5-l and TRGV5-2 identity values vs. TRGV1 of 79.8 and 79.4%, respectively suggesting their possible origin from this gene.

### Evolution of TRGJ blocks in Bovidae

**Figure 7:**
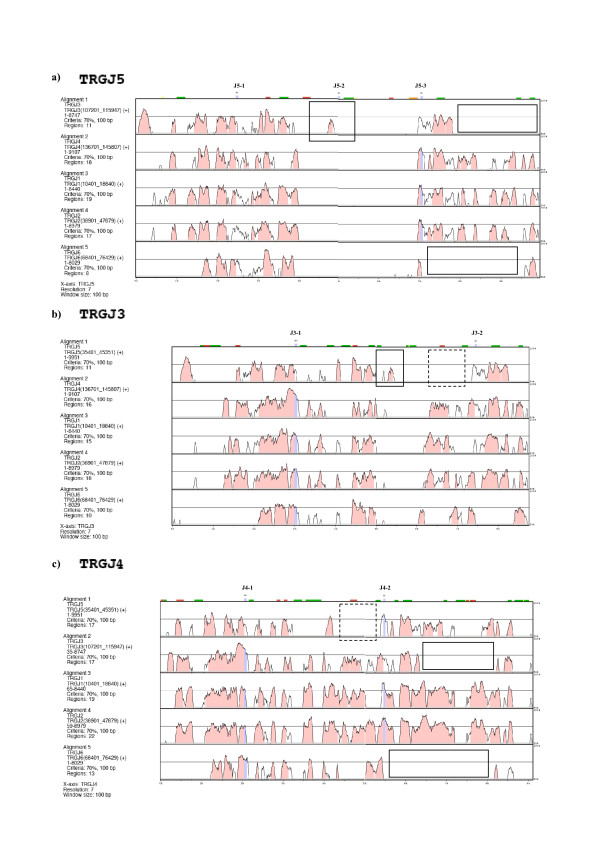
**mVISTA plots of the alignment of ovine TRGJ5, TRGJ3 and TRGJ4 blocks with all the others**. The query sequence is in bold while the others are listed on the left of each panel. Genes of the reference sequence (plotted at the top) are indicated by arrow-heads and exons are blue coloured; repeats are shown above the plot. Numbers on the x axis refer to positions within the reference sequence. Horizontal rectangles, squares and dotted squares are discussed in the text. For length and extension of each analysed sequence and parameters used see Materials and Methods.

**Figure 8:**
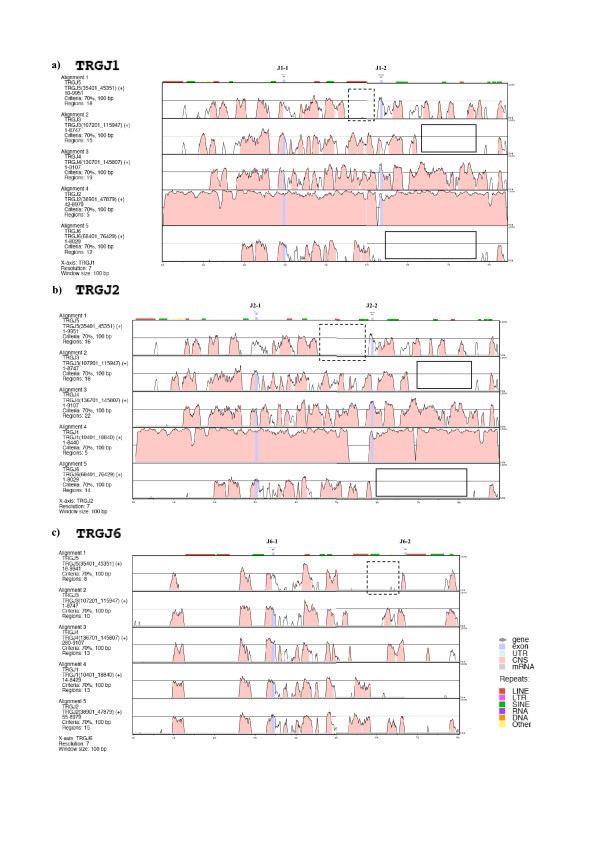
**mVISTA plots of the alignment of each ovine TRGJ1, TRGJ2 and TRGJ6 blocks with all the others**. The query sequence is in bold while the others are listed on the left of each panel. Genes of the reference sequence (plotted at the top) are indicated by arrow-heads and exons are blue coloured; repeats are shown above the plot. Numbers on the x axis refer to positions within the reference sequence. Horizontal rectangles, squares and dotted squares are discussed in the text. For length and extension of each analysed sequence and parameters used see Materials and Methods.

The results obtained so far are in accordance with previously obtained comparative data on Bovidae and human germline promoters located at the 5' of each C-distal J (Jd) gene. This data strongly supports a key role of such promoters in determining local accessibility to the site-specific recombinational machinery [27,31]. Figs. 7 and 8, generated using the mVISTA program, show the comparison of each TRGJ block with the others.

**Figure 9:**
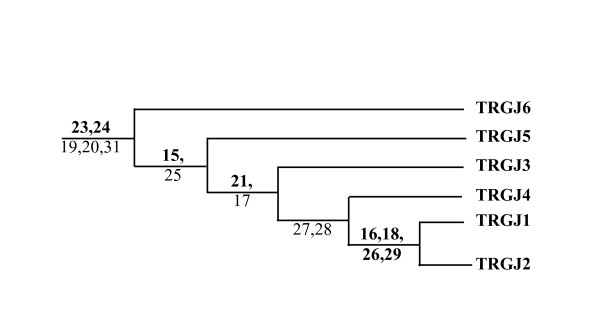
**Repeat elements as landmarks of evolution of TRGJ blocks**. The tree has been constructed considering the repeats content (RepeatMasker) of each cassette-specific TRGJ block. The repeats shared by two (bold) or more (regular) TRGJ blocks are numbered as in Table 2.

The mVISTA program provides a multi-alignment of nucleotide sequences and highlights their local similarity regions in the compared genomic regions. Indeed, since the insertion of a mobile element in a given position of the genome is a rather unique event, the presence in the same genomic position in different homologous regions can be used to infer a common origin [32,33]. The comparative analysis of the TRGJ blocks(dark green rectangles in Fig. 5), was particularly useful to formulate a plausible model of the formation and evolution of the ovine TRG loci. The analysis was conducted considering three different parameters: 1) the regions featuring a similarity higher than 70% shared by coding and non coding regions obtained by multialigned TRGJ blocks (Figs. 7 and 8); 2) the repeated elements shared by the six cassettes displayed in Table 2 and Fig. 9; 3) the similarity values obtained from comparing all the TRGJ regions using the mVISTA program (See Additional file 6, Table S6).

By looking at the so-called "peaks and valleys" mVISTA graphs obtained by comparison of each TRGJ block with all the others, the following considerations can be made: i) a high similarity is preserved between the regions right uphill and downstream of the C-distant J (Jd) (Figs. 7 and 8); ii) the region including the J5-2 between J5-1 and J5-3 (Fig. 7a), presents a similarity peak higher than 70% only in the alignment TRGJ5 vs. TRGJ3 and vice versa (squares in a e b); iii) the region immediately at 5' of the C-proximal J (Jp) gene in each unit seems to be similar in TRGJ6, TRGJ3, TRGJ4, TRGJ2 and TRGJ1 and different in TRGJ5 (dot line square in Fig. 7b, c and Fig. 8a, b, c); these regions immediately at 5'of each Jp gene have become homogeneous in all cassettes, with the exception of TRGJ5, maybe for one or more gene conversion events that initially involved only TRGJ3 and TRGJ6 blocks; iv) the TRGJ6 block, more significantly, and the TRGJ3 block would seem to be deleted in 3' Jp region (horizontal rectangles in Fig. 7a, c, and Fig. 8a, b).

In consideration of the above observations, the TRGJ blocks can be clearly sub-divided into two groups based on their "resemblance" and sharing of repeated elements. Some of these have provided valuable information suggesting the most likely succession of events.

One group consists of the TRGJ6 and TRGJ5 (Fig. 9), since these two blocks share repeats r23 e r24 that the remaining four blocks lack. r19, r20 and r31 are shared by all blocks. Finally, r22 is present in all blocks except TRGJ5, while r25, is always present except in TRGJ6. For these two repeats, we can hypothesize that a rearrangement, which could have generated the third J in TRGJ5, may have obscured their phylogenetic origin. The region including the J5-2 gene was subsequently lost in all cassettes except TRGC3, where only residual sequences from the region remain (Fig. 7). A series of specific shared repeats (See Table 2 and Additional file 7, Figure S7) substantiate the scenario depicted in the next section: r15 is shared only by TRGJ5 and TRGJ3, just like r21 is shared by TRGJ3 and TRGJ4. r17 keeps its sharing starting from TRGJ3, up to TRGJ4, TRGJ1 and TRGJ2, while r27 and r28 are shared by TRGJ4, TRGJ1 and TRGJ2. The most recent shared insertions, present only in blocks TRGJ1 and TRGJ2, are r16, r18, r26 and r29. This latter data, together with the similarities of the TRGV1 vs. TRGV5-2 rather than of TRGV1 vs. TRGV5-1, as mentioned above in the analysis ii) of the matrix in Fig. 5 and the set of data presented in Fig. 6, led us to assume the origin of cassette TRGC1 by duplication of cassette TRGC2.

**Figure 10:**
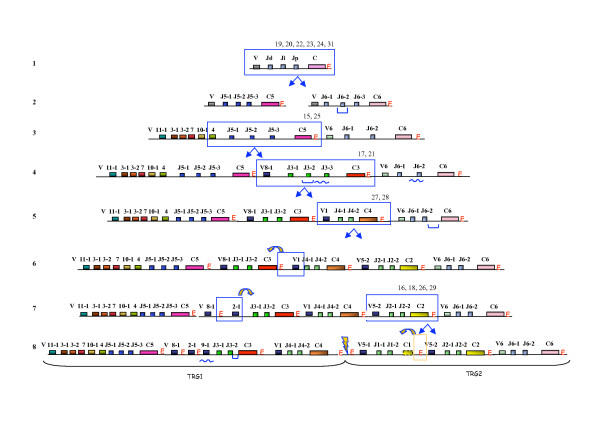
**Hypothetic evolutionary model for the ovine TRG loci**. The genes are represented (not in scale) as small coloured boxes with transcriptional orientation from left to right, while the enhancer-like sequences are indicated with a red coloured **E**. It is not clear whether the three-Js conformation of the most ancient cassette preceded or followed the formation of the ancestors of TRGC6 and TRGC5 cassettes (the most probable hypothesis is shown in lines 1 and 2). Rectangles, squared parentheses and wavy lines are used to indicate the regions involved in duplicative events, deletions and conversions, respectively. Duplications of a cassette or a gene are indicated by two blue coloured head-arrows or by a single dark yellow arrow while for the chromosome break a flash of lightning was used. The likely insertion of repeats, listed in Table 2 and shown in Fig. 9, is indicated with the corresponding number. Panels 1–8 are discussed in Results.

Concerning the shared repeats in genomic regions relating to the C genes, the parameter of the common repeats may be used only in the regions immediately at 5'(r29, r30 and r31), in I intron (r32 and r33) and immediately at 3'(r46 and r47) of the coding segment (Table 2). Repeated gene conversion, duplication and deletion events concerning exons II have probably obscured the evolutive relationship of these genes. However, the phylogenetic analysis conducted only on the coding regions of C genes [28] supports the succession of events hypothesized in the evolutionary scenario proposed in Fig. 10.

### A model for the evolution of the V-J-J-C cassettes in ovine TRG1 and TRG2 loci

The distribution of the "similarity peaks" (>70%) obtained through the mVISTA program by comparing each TRJG block against all the others (Figs. 7 and 8) as well as evidence from the presence or absence of repeated elements in different TRJG blocks (Fig. 9) and from similarity percentages between all TRJG blocks (See Additional file 6, Table S6) provided us the basis to propose a model for the formation of sheep TRG loci, although multiple gene conversion events, which complicate this analysis, have almost certainly occurred. The model will be discussed by referring in turn to each numbered panel of Fig. 10. We propose that after the duplication of a minimum ancestral cassette consisting of one V, three Js and one C genes, the ancestral TRG locus consisted of two cassettes, which had the repeated elements r23 and r24 in common and probably were the forerunners of the TRGC5 and TRGC6 cassettes. The ancestral cassette likely possessed three J genes, of which the central one was subsequently lost in the TRGC6 cassette by a deletion event (panel 2 of Fig. 10). This hypothesis is supported by the phylogenetic tree of the J genes, where the bootstrap value close to 85% indicates a clear subdivision of human and sheep sequences into two main groups. A third minor group consists of chicken J genes, human JP and sheep J5-2 [27]. Concerning the six V genes of the TRGC5 cassette, which present a low level of similarity with the V genes of the TRGC3, TRGC4, TRGC1 and TRGC2 cassettes, we hypothesize that they are the result of ancestral duplicative events which only involved V genes presumably in the same manner as occurred in the human locus (See the last subsection). The specific sharing of repeats and the dot plot comparison analysis substantiate our scenario that proposes (panel 4 of Fig. 10) the origin of the TRGC3 (V8-1, J3-1, J3-2, C3) cassette from TRGC5 (Vn.i., J5-1, J5-2, J5-3, C5). In addition, we postulate the occurrence of a deletion of the region including the centrally positioned TRGJ5-2 gene (panel 2) as well as gene conversion events that made the regions immediately to 5' of the Jp in TRGC3 more similar to the corresponding one of TRGC6 (panel 4) than to the one of TRGC5. Panel 5 shows the origin of TRGC4 (V1, J4-1, J4-2, C4) from a duplication of TRGC3. Insertion of repeats r3, r4, r5 and r7, shared by TRGV8-1 and TRGV1 (Table 2), and repeat r21 found in the TRGJ3 and TRGJ4 blocks (Table 2), most probably occurred at this stage.

Panel 6 of Fig. 10 hypothesizes a duplication of the region containing the 3'C3 enhancer-like sequence inclusive of TRGV1 that originated TRGV2-1 and also shows the origin of the TRGC2 cassette by a duplication of the TRGC4 cassette. Both events are proposed to have occurred at this stage because repeats r6 and r12, shared by TRGV2-1, TRGV1, TRGV5-1 and TRGV5-2 are likely to have been inserted at the same time. Panel 7 shows a second-step duplication of the TRGV2-1 region originating TRGV9-1. Repeat r14, which is shared by TRGV2-1 and TRGV9-1 only, could have been inserted at this time. The analysis of the repeats and of the phylogenetic tree of Fig. 6 favours a more recent origin of TRGV9-1 than TRGV2-1. However, this is in conflict with the dot plot analysis shown in Fig. 2, which indicates that TRGV9-1, in contrast to TRGV2-1 originated directly from TRGV1 or from a gene conversion event. Panel 7 also shows the origin of the TRGC1 cassette depicted in panel 8 by duplication of the entire TRGC2.

This reconstruction of the sequence of ancient duplications events illustrated in Fig. 10 is supported not only by the similarity levels observed (See Additional file 6, Table S6) but also by the observed concordances in the distribution of the repeated elements: r19, r20 and r31 are shared by all six TRGJ blocks; repeat r15 is TRGC5 and TRGC3 specific; repeat r21 is TRGC3 and TRGC4 specific, r27 and r28 are TRGC4, TRGC2 and TRGC1 specific, and finally r16, r18, r26, r29 and r30, shared by TRGC2 and TRGC1 cassettes, seem to have been the last inserted in the TRG locus. A further duplication event concerning the 3'C1 enhancer-like sequence region (see panel 8) is proposed to have originated a region containing two adjoining enhancer-like sequences following the genesis of the six cassettes. The TRG2 locus is assumed to have later originated by a chromosomal rearrangement that probably occurred in the region where two-adjoining enhancer sequences were located at the 3' of the C4 gene and at the 5' of the TRGV5-1 gene respectively (panel 8). As a result, a translocation event removed the part of the locus consisting of TRGC1, TRGC2 and TRGC6 cassettes from the 4q3.1 band taking it to the 4q2.2 band.

There is no doubt that some aspects of the model may be incorrect, especially because some evolutive relationships might may have been obscured by gene conversion. However, the model highlights the genetic "fluidity" of this region, that led to a strikingly variable and complex genetic locus in Bovidae.

### Comparison of human, murine and Bovidae TRG loci

When comparing the sheep genomic organization described above to the human TRG locus [18,20,34], we find that in Bovidae (cattle, sheep and goat) a significant number of duplicative events has led to a substantial increase in the number of V, J and C genes, to the point of colonizing an additional region of chromosome 4, while in man duplications are considerable only in the region of V genes. The human TRG locus spans 160 Kb and features in its 5' region fourteen variable genes, of which only six are functional, grouped into six subgroups according to the identity values [18,20].

PipMaker dotplot matrixes of the ovine TRG1 and TRG2 loci, against the human TRG locus display two identity diagonals in line with the J-C regions of each ovine cassette (See Additional file 8, Figure S8a, b). This result reflects the organisation of the two J-C regions duplicated in tandem in man, which are superimposed "end to end": the first consists of three J genes (TRGJP1, TRGJP and TRGJ1) and one C gene (TRGC1) while the second consist of two J genes (TRGJP2 and TRGJ2) and one C gene (TRGC2), all functional. In both matrixes, the longest diagonals refer to block JP2-J2-C2. The apparent greater length is due to the small diagonals located downstream of each ovine C and corresponding to the single human enhancer sequence. The nine variable genes of the human TRGV1 subgroup, all located at 5' of the locus, include three pseudogenes (TRGV5P, TRGV6 and TRGV7) and one ORF (TRGV1) [18,20], and display short similarity traits, only in the coding part against both the V genes of TRGC3 and TRGC4 cassettes and those of TRGC1 and TRGC2. The human functional TRGV9 displays similarity only with TRGV11-1 of TRGC5, while TRGV10 and TRGV11 (ORFs) and TRGVB (pseudogene) recognize the areas corresponding to TRGV3-1, TRGV3-2 and TRGV7. Finally, the single TRGVA (pseudogene) features one similarity diagonal including both its coding and non-coding parts against TRGV6-1 of TRGC6 (green square) (See Additional file 8, Figure S8b). When looking at the human vs. Bovidae matrixes, the importance that the J-J-C blocks must have had during the evolution of these loci emerges clearly. Indeed, these regions, by preserving their intergenic portions, behave quite differently than other regions of the same locus, as for example those including the V genes, for which a strong intra-species and inter-species sequence divergence has been reported [17]. The phylogenetic analyses of the J genes [27] are in full agreement with this sort of preservation of a spatial constriction in the J-C regions, at least when considering human and Bovidae species comparison.

The genomic organisation of the murine TRG locus [GenBank: AF037352] [21] is apparently similar to that of Bovidae, as it features four V-J-C cassettes for a total length of about 200 Kb on chromosome 13. The TRGC1 cassette, which correlates to human, has four V genes, all of which are functional, one J gene (TRGJ1) and one C gene (TRGC1) [34] (See Additional file 9, Figure S9a, b); the TRGC3, TRGC2 and TRGC4 cassettes all consist of one V, one J and one C, which are non functional in TRGC3 and display a transcriptional orientation inverted with respect to the other three cassettes in TRGC2. A sequence putatively serving as an enhancer is found downstream of each C gene. However, the resemblance to the Bovidae genomic organisation is only apparent because in PipMaker dotplot matrices obtained by comparison of each sheep TRG locus with the murine TRG1 cassette, only short similarity traits corresponding to exonic regions are evident. In the three gray rectangles in particular, three spots are indicated, which correspond to the J gene and to the first and last exon of the constant genes, respectively (See Additional file 9, Figure S9a). The murine TRGV7 gene recognizes all the V genes of TRGC3 and TRGC4 cassettes (blue circles). On the other hand, the murine TRGV4 is recognized by TRGV3-1, TRGV3-2 and TRGV7 while murine TRGV6 and TRGV5 are recognized by TRGV10-1 and TRGV4, respectively (red rectangle). As regards the matrix obtained by comparison of the ovine TRG2 locus vs. the murine TRG1 cassette, only murine TRGV7 recognizes TRGV5-1 and TRGV5-2, respectively (See Additional file 9, Figure S9b). The similarity relationship between V genes in a three species comparison (not shown) highlights the absence in the murine locus of the TRGVA and TRGV9 human genes and the ovine TRGV6-1 and TRGV11-1 genes, and the absence of ovine TRGV10-1 and TRGV4 and murine TRGV6 and TRGV5 genes in the human locus. The human/murine/Bovidae comparison revealed that the V genes in the TRGC5 cassette are highly related across species and lie in a syntenic region, indicating that this cassette existed before the primate – rodent – artiodactyl lineages diverged.

## Conclusion

The main objective of this study was to focus on the evolutionary history of the two TRG loci in Bovidae (cattle and sheep) as compared to the single human locus. The contiguous genomic sequence has allowed us to define the genomic structure of the sheep TRG1 and TRG2 loci that emphasises the peculiarity of the organization of the Bovidae loci in cassettes, each containing the basic V-J-J-C unit; on the other hand, the human TRG locus features duplicated J-C genes separated from the set of V genes [18]. The analysis of the dot plot similarity matrix comparing the two sheep loci allowed us to propose an evolutionary model explaining the origin of two paralogous regions in chromosome 4 of Bovidae. Evidence from concordant insertions of repetitive elements strongly supports the interaction of the J-J-C regions in driving the sequence of duplicative events. J-J-C regions are delimited at their 5' end by promoters for germline transcription containing STAT motifs, which control the local recombinational accessibility [31] and at their 3' end by enhancer-like elements, which govern the general recombinational accessibility. The phylogenetic conservation of the eight enhancer-like elements found in sheep compared with the single copy present in the human indicates that they play a key role in the functional organization of the Bovidae TRG loci. Our proposed model suggests that only duplicated entire J-J-C regions, which possessed an enhancer-like element at their 3' end, and at least one variable gene at their 5' end, were selected and fixed as functional cassettes. Requirements related to immunoprotective functions, which include mechanisms establishing a first defensive barrier in the digestive tract of ruminant animals, are likely to have induced a sort of genomic fluidity of this region favouring its evolution by reiterated duplications. Interspecies comparison of TRG loci reveals that Bovidae and human loci, although apparently not correlated in general structure, share extensive colinearity and high similarity levels in the regulatory and intergenic as well as in the coding regions. On the other hand, Bovidae and murine TRG loci, although almost identical in genomic structure, show significant similarity only in the genic sequences. Studies in other ruminant families will be needed to further clarify evolutionary events and answer questions regarding how genetic fluidity is generated in genomic loci determining "γδ high" condition. We suggest that an extended phylogenetic analysis comparing ovine-bovine sequences with other bovid species might provide further insight into the evolutionary history of the TRG locus in artiodactyls and mammals in general, and provide some clues to support functional studies through phylogenetic footprinting.

## Methods

### Construction of the contigs

Ovine TRG1 [GenBank: DQ992075] and TRG2 [GenBank: DQ992074] contigs were assembled from single sequences of overlapping or contiguous plasmid subclones obtained by PCR or restriction digestion of various genomic DNA sources: BAC clones from the "Romanov" ovine breed [35], Lambda phage clones from the "Altamurana" breed [28] and genomic DNA from the "Gentile di Puglia" breed. A detailed list of these subclones is provided (See Additional file 10, Tables 10Sa, b).

Bovine TRG1 and TRG2 sequences were retrieved from the accession numbers [GenBank: AY644517], [NW_937068] and [GenBank: AY644518], respectively.

Human TRG sequence: position 1–140728 of the GenBank entry [GenBank: NG_001336]; position 140729–141380 of the GenBank entry [GenBank: AC006033] (Complement 53466–54680).

Murine TRGC1 correspond to position 1–50000 of the GenBank entry [GenBank: AF037352].

### Bioinformatic analyses

For the detection of repeats, the Tandem Repeats Finder [36,37] and RepeatMasker [38] programs were used. TRF locates and displays tandem repeats in DNA sequences with pattern size in the range from 1 to 2000 bases. RepeatMasker screens DNA sequences for interspersed repeats and low complexity DNA sequences. The RepeatMasker analysis has been carried out using the Cetartiodactyla Repbase division section.

### Multialignment of genomic contigs

For the comparative analyses of genomic sequences two bioinformatic tools were used: PipMaker and mVISTA. PipMaker [39,40] computes alignments of similar regions in two DNA sequences. The resulting alignments are summarized with a "percent identity plot", or "pip", a dotplot matrix, a report of the relevant annotation for the occurrence of genes, interspersed repeats and CpG islands. The comparative analysis of genomic sequences providing peak and valley conservation plot has been obtained using mVISTA [41]. The extension of each analysed TRGJ blocks was established in an approximative manner on the basis of the conserved regions identified by the PipMaker program and shown in the corresponding dotplots. More precisely they were on TRG1 contig: 35401–45351 (TRGJ5 block), 107201–115947 (TRGJ3 block), 136701–145807 (TRGJ4 block); on TRG2 contig: 10401–18840 (TRGJ1 block), 38901–47879 (TRGJ2 block), 68401–76429 (TRGJ6 block).

### Construction of the enhancer-like sequences phylogram tree

The phylogenetic tree has been constructed on 506 ungapped sites of the enhancer alignment by using the PAUP* software with the Neighbor Joining method on GTR distances. The ovine sequences identical to the one (position 140573–141360 of the contig [GenBank: NG_001336] [GenBank: AC006033]) containing the human TRG locus enhancer (located downstream of C2 gene), in particular: positions 89–852 (5'TRGV5-1), 28460–29461 (3'C1), 59712–60714 (3'C2), 92330–93111 (3'C6) of the TRG2 contig; and positions 56800–57555 (3'C5), 78380–79154 (5'TRGV2-1), 97280–98048 (5'TRGV9-1), 124645–125360 (3'C3) of the TRG1 contig were multialigned. The identity at 3'C4 gene (ovine TRG1 locus) was not included in the multialignment because of the partial relevant genomic sequence.

## Abbreviations

IMGT: IMGT^®^, the international ImMunoGeneTics information system^®^, TRG: T cell receptor gamma; V-J-J-C: cassette [genomic unit in immunoglobulin (IG) or T cell receptor (TR) loci];

V: variable gene ; J: joining gene; C: constant gene.

## Authors' contributions

GV, MCM and SC designed the experiments. GV carried out the construction of the sheep TRG1 contig and performed the bioinformatic analyses. MCM carried out the construction of the sheep TRG2 contig. RA participated in the design of the study and was involved in revising the manuscript. GP participated in the bioinformatic analyses and interpretation of data. GV and SC wrote the manuscript, which was read and approved by all authors.

## Supplementary Material

Additional File 1 **Table S1a, b – Tandem repeats in TRG1 (a) and TRG2 (b) sheep loci**. Tables presenting list of the tandem repeats obtained by the Tandem Repeat Finder program. Click here for file

Additional File 2 **Table S2a, b – Repeats content of TRG1 and TRG2 sheep loci**. Tables presenting repeats content obtained by the RepeatMasker program reported as a summary (a, b). Click here for file

Additional File 3 **Table S3c, d – Repeats content of TRG1 and TRG2 sheep loci**. Tables presenting repeats content obtained by the RepeatMasker program reported in details (c, d). Click here for file

Additional File 4 **Figure S4 – Genomic comparison of ovine and bovine TRG1 loci**. The alignment of ovine TRG1 locus and bovine TRG1 locus [GenBank: AY644517] was visualized as a dotplot matrix obtained with the PipMaker program. The gene transcriptional orientation is indicated by arrows. Click here for file

Additional File 5 **Figure S5 – Genomic comparison of ovine and bovine TRG2 loci**. The alignment of ovine TRG2 locus and bovine TRG2 locus [GenBank: AY644518] was obtained by the AVID program through the mVISTA server. The percent of conservation between the two hortologous sequences is reported on the vertical axis and is visualized as pink regions (conserved non-coding sequences or CNS) or dark blue regions (exon sequences). Genes are indicated by arrows; repeats are shown above the plot. Click here for file

Additional File 6 **Table S6 – Similarity percentages of ovine TRGJ blocks pairwise alignments**. Table presenting similarity percentages of ovine TRGJ blocks pairwise alignments (mVISTA). Click here for file

Additional File 7 **Figure S7 – Alignments of the retropositions in the six investigated TRGJ blocks**. LINE L2, Sine MIRb, SINE BovA2, LINE L1M5, SINE BovtA2, CHR-L, SINE BovtA3, LINE L1 sequences are in upper case letters. The 5' and 3' ends of the retroposon insertions are boxed. For the detection of repeats, the Tandem Repeats Finder [36,37] and RepeatMasker [38] programs were used. The RepeatMasker analysis has been carried out using the Cetartiodactyla Repbase division section. Click here for file

Additional File 8 **Figure S8a, b – Genomic comparison of ovine and human TRG loci**. The dotplots were obtained with the PipMaker program by using the complete sequences of ovine TRG1 and human TRG [GenBank: NG_001336] [GenBank: AC006033] (a) and ovine TRG2 and human TRG (b). The gene transcriptional orientation is indicated by arrows. The red rectangle includes similar regions between V genes belonging to ovine TRGC5 cassette and human V11, VB, V10 (a) while green rectangle highligths the similarity between V6-1 belonging to ovine TRGC6 cassette and human VA (b). Blue circles highlight similar regions between ovine V1, V2, V5, V8, V9 gene subgroups and human TRGV1 subgroup genes (a and b). The gray rectangles include conserved J-C containing regions, whereas gray arrows point to ovine enhancer-like sequences. Click here for file

Additional File 9 **Figure S9a, b – Genomic comparison of ovine and murine TRG loci**. The dotplots were obtained with the PipMaker program by using the complete sequences of ovine TRG1 and murine TRG1 cassette [GenBank: AF037352] (a) and ovine TRG2 and murine TRGC1 cassette (b). The gene transcriptional orientation is indicated by arrows. The red rectangle includes similar regions between V genes belonging to ovine TRGC5 cassette and murine V4, V6, V5 (a). Blue circles highlight similar regions between ovine V1, V2, V5, V8, V9 gene subgroups and murine V7 (a and b). The gray rectangles include conserved J-C containing regions. Click here for file

Additional File 10 **Table S10a, b – Plasmid subclones covering the entire sheep TRG1 and TRG2 loci**. Tables presenting list of the plasmid subclones covering the entire sheep TRG1 (a) and TRG2 (b) loci. Click here for file
