# Supplementary material for: Genomic organization and recombinational unit duplication-driven evolution of ovine and bovine T cell receptor gamma loci
Source: BMC Genomics. 2008 Feb 18;9:81. doi: 10.1186/1471-2164-9-81 (PMC2270265; doi:10.1186/1471-2164-9-81)
Supplement: Additional File 1 — Table S1a, b – Tandem repeats in TRG1 (a) and TRG2 (b) sheep loci. Tables presenting list of the tandem repeats obtained by the Tandem Repeat Finder program. [file 1471-2164-9-81-S1.pdf]

Tandem repeats in TRG1 (a) and TRG2 (b) loci.

a)

Indices [a]	Period Size	Copy Number	Consensus Size	Percent Matches	Percent Indels	Score	A	C	G	T	Entropy (0-2)
3671--3704	12	2.8	12	90	4	50	35	0	11	52	1.38
5377--5407	15	2.1	15	93	0	53	38	32	25	3	1.72
19400--19487	27	3.1	30	75	12	94	15	11	12	60	1.59
19406--19478	13	5.2	13	71	12	56	15	12	10	61	1.56
21735--21782	2	23.5	2	95	4	87	0	2	50	47	1.12
38402--38431	15	2.0	15	100	0	60	20	33	0	46	1.51
39161--39204	23	1.9	23	86	9	63	27	9	6	56	1.55
40798--40826	3	9.7	3	100	0	58	34	31	34	0	1.58
64145--64187	17	2.3	20	76	23	65	23	9	0	67	1.19
64148--64183	17	2.1	17	100	0	72	27	5	0	66	1.13
66582--66642	21	2.8	21	80	5	68	54	3	37	4	1.39
71380--71405	3	8.7	3	100	0	52	0	34	30	34	1.58
74306--74366	29	2.1	28	96	3	113	22	19	39	18	1.92
75961--76007	2	23.5	2	100	0	94	0	0	51	48	1.00
76262--76654	142	2.8	142	95	1	691	20	30	27	21	1.98
90624--90672	4	11.3	4	83	16	62	67	0	28	4	1.09
90628--90668	14	2.9	14	100	0	82	65	0	29	4	1.13
90617--90676	18	3.6	18	82	17	92	65	0	28	6	1.18
101848--101882	2	17.5	2	100	0	70	0	48	0	51	1.00
101891--101950	29	2.0	30	93	3	104	51	18	0	30	1.46
109058--109093	14	2.5	14	95	4	63	41	13	27	16	1.87
113003--113029	5	5.4	5	100	0	54	44	18	18	18	1.87
119246--119275	9	3.3	9	100	0	60	13	43	10	33	1.77
119939--119971	12	2.8	12	86	4	50	42	30	6	21	1.77
131295--131339	17	2.6	17	100	0	90	60	0	31	8	1.28
158185--158215	14	2.2	14	94	0	53	9	29	16	45	1.79

b)

Indices [a]	Period Size	Copy Number	Consensus Size	Percent Matches	Percent Indels	Score	A	C	G	T	Entropy (0-2)
2903--2944	19	2.2	19	83	8	50	33	21	21	23	1.97
5241--5306	18	3.7	18	91	0	114	43	6	43	6	1.53
22563--22608	17	2.6	18	89	6	76	45	0	0	54	0.99
22553--22638	42	2.0	42	84	4	111	44	4	0	51	1.22
33998--34081	18	4.7	18	93	0	150	40	5	47	5	1.52
51878--51914	13	2.7	14	84	16	51	45	13	0	40	1.43
52237--52282	15	3.1	15	100	0	92	28	39	13	19	1.89
64726--64767	2	21.0	2	100	0	84	50	0	0	50	1.00
68199--68256	19	3.0	19	97	2	107	15	25	20	37	1.92
77383--77474	46	2.0	46	100	0	184	28	30	8	32	1.87
80026--80063	19	2.0	19	100	0	76	42	15	15	26	1.87
82861--82917	2	28.5	2	100	0	114	49	0	0	50	1.00
88733--88764	15	2.1	15	94	0	55	28	31	34	6	1.82
88912--88960	2	24.5	2	87	0	71	46	46	4	2	1.33

[a] Repeat position in the contig.
