# Supplementary material for: Genomic organization and recombinational unit duplication-driven evolution of ovine and bovine T cell receptor gamma loci
Source: BMC Genomics. 2008 Feb 18;9:81. doi: 10.1186/1471-2164-9-81 (PMC2270265; doi:10.1186/1471-2164-9-81)
Supplement: Additional File 2 — Table S2a, b – Repeats content of TRG1 and TRG2 sheep loci. Tables presenting repeats content obtained by the RepeatMasker program reported as a summary (a, b). [file 1471-2164-9-81-S2.pdf]

Repeats content of TRG1 and TRG2 loci.
a)

```
==================================================
file name: RM2_TRG1ov.txt_114493219
sequences:            1
total length:     158873 bp  (158873 bp excl N/X-runs
GC level:         40.71 %
bases masked:      48282 bp ( 30.39 %)
==================================================
               number of      length   percentage
               elements*    occupied  of sequence
--------------------------------------------------
SINEs:              168        25926 bp    16.32 %
      Alu/B1          0            0 bp     0.00 %
      MIRs           60         9201 bp     5.79 %

LINEs:               57        18615 bp    11.72 %
      LINE1          32         8606 bp     5.42 %
      LINE2           5          436 bp     0.27 %
      L3/CR1          2          153 bp     0.10 %
      RTE            18         9420 bp     5.93 %

LTR elements:         5          813 bp     0.51 %
      MaLRs           2          491 bp     0.31 %
      ERVL            2          251 bp     0.16 %
      ERV_classI      0            0 bp     0.00 %
      ERV_classII     0            0 bp     0.00 %

DNA elements:         8          864 bp     0.54 %
      MER1_type       5          548 bp     0.34 %
      MER2_type       0            0 bp     0.00 %

Unclassified:         0            0 bp     0.00 %

Total interspersed repeats:    46218 bp    29.09 %


Small RNA:            2          120 bp     0.08 %

Satellites:           0            0 bp     0.00 %
Simple repeats:      21          880 bp     0.55 %
Low complexity:      19         1064 bp     0.67 %
==================================================
*most repeats fragmented by insertions or deletions
 have been counted as one element
```

b)

```
==================================================
file name: RM2_TRG2ov.txt_114493334
sequences:            1
total length:      95092 bp  (95091 bp excl N/X-runs)
GC level:         40.68 %
bases masked:      30987 bp ( 32.59 %)
==================================================
               number of      length   percentage
               elements*    occupied  of sequence
--------------------------------------------------
SINEs:                90       14277 bp    15.01 %
      Alu/B1           0           0 bp     0.00 %
      MIRs            37        5642 bp     5.93 %

LINEs:                41       14460 bp    15.21 %
      LINE1           25        9208 bp     9.68 %
      LINE2            3         370 bp     0.39 %
      L3/CR1           1          93 bp     0.10 %
      RTE             12        4789 bp     5.04 %

LTR elements:          1          69 bp     0.07 %
      MaLRs            0           0 bp     0.00 %
      ERVL             0           0 bp     0.00 %
      ERV_classI       0           0 bp     0.00 %
      ERV_classII      0           0 bp     0.00 %

DNA elements:          5         629 bp     0.66 %
      MER1_type        5         629 bp     0.66 %
      MER2_type        0           0 bp     0.00 %

Unclassified:          0           0 bp     0.00 %

Total interspersed repeats:    29435 bp    30.95 %


Small RNA:             0           0 bp     0.00 %

Satellites:            0           0 bp     0.00 %
Simple repeats:       12         598 bp     0.63 %
Low complexity:        9         954 bp     1.00 %
==================================================
*most repeats fragmented by insertions or deletions
 have been counted as one element
```
