# Supplementary material for: Genomic organization and recombinational unit duplication-driven evolution of ovine and bovine T cell receptor gamma loci
Source: BMC Genomics. 2008 Feb 18;9:81. doi: 10.1186/1471-2164-9-81 (PMC2270265; doi:10.1186/1471-2164-9-81)
Supplement: Additional File 3 — Table S3c, d – Repeats content of TRG1 and TRG2 sheep loci. Tables presenting repeats content obtained by the RepeatMasker program reported in details (c, d). [file 1471-2164-9-81-S3.pdf]

Repeats content of TRG1 and TRG2 loci.

c)

SW score[a]	perc div.[b]	perc del.[c]	perc ins.[d]	query sequence	position in query[e] begin	end	(left)[f]		matching repeat[g]	repeat class/family	position in repeat[h] begin	end	(left)[i]	ID	
339	31.0	5.4	0.8	TRG1	332	461	(158412)	+	MIRb	SINE/MIR	28	163	(105)	1	
350	33.5	5.0	2.5	TRG1	812	970	(157903)	+	MIRb	SINE/MIR	30	192	(76)	2	
187	29.2	5.4	2.7	TRG1	1095	1168	(157705)	C	MIRb	SINE/MIR	(157)	111	36	3	
182	25.6	12.9	3.2	TRG1	3174	3266	(155607)	C	L3	LINE/CR1	(3684)	805	704	4	
260	22.8	6.3	0.0	TRG1	3571	3649	(155224)	+	L1_Art	LINE/L1	2370	2453	(179)	5	
187	13.3	2.2	2.2	TRG1	3660	3705	(155168)	+	(TATATG)n	Simple_repeat	3	48	(0)	6	
488	17.5	0.0	4.6	TRG1	3715	3822	(155051)	+	L1_Art	LINE/L1	2465	2567	(65)	5	
26	0.0	0.0	0.0	TRG1	3970	3995	(154878)	+	AT_rich	Low_complexity	1	26	(0)	7	
414	17.9	8.2	1.2	TRG1	4012	4096	(154777)	C	CHRL	SINE/tRNA-Glu	(77)	91	1	8	
233	26.7	6.5	2.2	TRG1	4838	4929	(153944)	+	MIRb	SINE/MIR	1	96	(172)	9	
614	8.2	4.8	4.8	TRG1	4958	5060	(153813)	+	BOV-A2	SINE/BovA	32	134	(138)	10	
1437	13.7	0.0	0.5	TRG1	5676	5887	(152986)	+	BovB	LINE/RTE	3088	3298	(4)	11	
367	15.7	3.3	0.0	TRG1	5973	6048	(152825)	+	Bov-tA3	SINE/BovA	143	270	(2)	12	
227	28.3	5.0	7.1	TRG1	6729	6827	(152046)	C	CHR-1	SINE/tRNA-Glu	(0)	115	19	13	
382	32.0	3.1	0.0	TRG1	7942	8069	(150804)	+	LTR33	LTR/ERVL	6	137	(384)	14	
261	35.8	4.1	0.0	TRG1	8415	8537	(150336)	+	LTR33	LTR/ERVL	3	130	(391)	15	
1536	11.9	0.0	0.0	TRG1	8896	9096	(149777)	+	Bov-tA2	SINE/BovA	9	209	(3)	16	
364	19.9	5.3	7.4	TRG1	9118	9290	(149583)	+	L1MA9	LINE/L1	5875	6039	(364)	17	
232	23.5	1.5	0.0	TRG1	9381	9448	(149425)	+	L1_Art	LINE/L1	2483	2551	(81)	18	
6577	5.2	0.0	0.0	TRG1	10970	11750	(147123)	+	BovB	LINE/RTE	2522	3302	(0)	19	
368	20.9	0.0	0.0	TRG1	11837	11903	(146970)	+	Bov-tA3	SINE/BovA	141	207	(6)	20	
440	33.9	1.1	0.7	TRG1	13250	13531	(145342)	+	L1_SS	LINE/L1	1962	2244	(4574)	21	
432	27.2	5.4	2.1	TRG1	13788	14027	(144846)	+	L1M4c	LINE/L1	2181	2428	(3956)	22	
238	14.6	5.5	0.0	TRG1	14151	14205	(144668)	C	L1_Art	LINE/L1	(74)	2558	2501	23	
365	32.0	0.0	1.6	TRG1	14318	14441	(144432)	+	MIRb	SINE/MIR	66	187	(81)	24	
408	19.5	2.3	1.1	TRG1	16048	16135	(142738)	+	CHRL	SINE/tRNA-Glu	1	89	(79)	25	
316	33.7	3.2	5.8	TRG1	16352	16540	(142333)	C	MIR	SINE/MIR	(44)	218	35	26	
1037	21.5	2.0	0.5	TRG1	16584	16779	(142094)	+	Bov-tA2	SINE/BovA	8	206	(6)	27	
247	16.9	7.3	19.8	TRG1	17199	17294	(141579)	+	CHR-2B	SINE/tRNA-Glu	1	84	(239)	28	*
1048	15.8	1.0	5.5	TRG1	17215	17415	(141458)	+	Bov-tA1	SINE/BovA	24	215	(12)	29	
400	29.7	5.3	0.3	TRG1	17440	17738	(141135)	C	L1_Art	LINE/L1	(73)	2559	2226	30	
1367	16.2	0.0	0.0	TRG1	17745	17948	(140925)	+	Bov-tA2	SINE/BovA	5	208	(4)	31	
335	30.1	6.5	1.5	TRG1	18675	18951	(139922)	+	L1MCa	LINE/L1	1530	2381	(4715)	32	
218	23.1	0.0	4.9	TRG1	19405	19486	(139387)	+	(TTTA)n	Simple_repeat	2	79	(0)	33	
703	15.7	0.0	0.0	TRG1	19493	19594	(139279)	C	CHRL	SINE/tRNA-Glu	(64)	104	3	34	
323	16.7	0.0	0.0	TRG1	19599	19658	(139215)	+	L1MCa	LINE/L1	2309	2368	(3947)	32	
299	26.4	2.3	0.0	TRG1	21571	21657	(137216)	+	CHRL	SINE/tRNA-Glu	2	90	(78)	35	
383	0.0	0.0	2.1	TRG1	21735	21782	(137091)	+	(TG)n	Simple_repeat	1	47	(0)	36	

	2142	10.4	2.1	0.0	TRG1	21935	22223	(136650)	+	BovB	LINE/RTE	3008	3302	(0)	37
	477	16.4	0.0	0.0	TRG1	22319	22391	(136482)	+	Bov-tA3	SINE/BovA	141	213	(0)	38
	362	32.1	4.2	2.8	TRG1	22804	22947	(135926)	C	MIRb	SINE/MIR	(28)	240	95	39
	455	32.5	2.5	3.5	TRG1	23280	23480	(135393)	C	MIR	SINE/MIR	(44)	218	20	40
	539	18.9	0.0	7.0	TRG1	24428	24541	(134332)	+	CHR-2B	SINE/tRNA-Glu	17	122	(201)	41
	2153	14.3	2.5	0.0	TRG1	24757	25070	(133803)	+	CHR-2A	SINE/tRNA-Glu	1	322	(0)	42
	26	0.0	0.0	0.0	TRG1	25149	25174	(133699)	+	AT_rich	Low_complexity	1	26	(0)	43
	455	31.2	8.7	1.2	TRG1	25307	25478	(133395)	+	THER1_MD	SINE/MIR	77	261	(13)	44
	743	7.1	2.0	0.0	TRG1	27666	27763	(131110)	C	CHRL	SINE/tRNA-Glu	(67)	101	2	45
	477	28.9	4.5	1.8	TRG1	27765	28042	(130831)	+	L1MDa	LINE/L1	1617	2011	(4692)	46
	3076	8.9	0.0	0.0	TRG1	28790	29181	(129692)	+	BovB	LINE/RTE	2911	3302	(0)	47
	371	19.7	1.5	0.0	TRG1	29277	29342	(129531)	+	Bov-tA3	SINE/BovA	141	207	(6)	48
	257	36.7	3.3	0.8	TRG1	31425	31545	(127328)	+	MIRb	SINE/MIR	18	141	(127)	49
	781	26.7	8.5	7.5	TRG1	31558	31982	(126891)	C	L1_Art	LINE/L1	(70)	2562	2134	50
	549	14.0	4.0	0.0	TRG1	32671	32770	(126103)	C	CHRL	SINE/tRNA-Glu	(64)	104	1	51
	415	32.2	8.5	1.1	TRG1	33545	33720	(125153)	C	MIR3	SINE/MIR	(1)	207	19	52
	899	9.4	0.0	0.0	TRG1	33941	34067	(124806)	C	BOV-A2	SINE/BovA	(3)	269	143	53
15	428	36.6	6.9	0.0	TRG1	35291	35465	(123408)	C	MIRb	SINE/MIR	(58)	210	24	54
16	30	5.9	0.0	0.0	TRG1	36035	36085	(122788)	+	AT_rich	Low_complexity	1	51	(0)	55
18	1316	14.8	0.0	0.5	TRG1	36401	36604	(122269)	+	Bov-tA2	SINE/BovA	7	209	(3)	56
	194	20.7	6.7	3.3	TRG1	38303	38362	(120511)	C	L3_Mars	LINE/CR1	(1771)	2651	2590	57
19	233	26.5	15.3	2.6	TRG1	38581	38686	(120187)	C	L2	LINE/L2	(2)	3417	3285	58
20	417	26.0	17.9	3.8	TRG1	38918	39129	(119744)	C	MIRb	SINE/MIR	(0)	268	27	59
	254	15.8	26.3	0.0	TRG1	39490	39565	(119308)	+	L1_Art	LINE/L1	2326	2421	(211)	60
23	2047	8.1	0.0	0.0	TRG1	40538	40797	(118076)	+	BOV-A2	SINE/BovA	1	260	(12)	61
	264	3.0	0.0	0.0	TRG1	40798	40830	(118043)	+	(CAG)n	Simple_repeat	2	34	(0)	62
	325	20.4	20.7	2.7	TRG1	41652	41762	(117111)	C	L1ME4a	LINE/L1	(11)	6110	5980	63
	189	29.8	3.5	3.5	TRG1	41783	41869	(117004)	+	THER1_MD	SINE/MIR	160	246	(28)	64
24	22	3.5	0.0	0.0	TRG1	42117	42145	(116728)	+	AT_rich	Low_complexity	1	29	(0)	65
	356	25.8	9.1	0.0	TRG1	42149	42346	(116527)	C	Kanga2_a	DNA/Tc2	(79)	809	594	66
25	470	33.0	11.8	0.0	TRG1	42821	43023	(115850)	+	MIRb	SINE/MIR	25	251	(17)	67
30	698	10.5	0.9	0.0	TRG1	44785	44889	(113984)	+	Bov-tA3	SINE/BovA	108	213	(0)	68
31	259	26.5	15.0	0.8	TRG1	45106	45238	(113635)	+	MIRb	SINE/MIR	51	202	(66)	69
32	1068	12.2	9.0	0.1	TRG1	45839	46025	(112848)	+	Bov-tA2	SINE/BovA	1	204	(8)	70
33	247	31.5	8.3	1.5	TRG1	46046	46177	(112696)	+	MIRb	SINE/MIR	104	244	(24)	71
	1523	8.6	5.0	0.0	TRG1	46472	46669	(112204)	C	Bov-tA2	SINE/BovA	(0)	212	5	72
	1074	12.5	6.2	0.0	TRG1	46860	47035	(111838)	C	Bov-tA2	SINE/BovA	(10)	202	16	73
	929	12.2	0.0	0.0	TRG1	47059	47189	(111684)	C	BOV-A2	SINE/BovA	(0)	272	142	74
34	231	36.0	7.8	8.1	TRG1	47798	48157	(110716)	C	L4	LINE/RTE	(184)	1776	1418	75
	22	0.0	0.0	0.0	TRG1	48269	48290	(110583)	+	AT_rich	Low_complexity	1	22	(0)	76
37	1963	4.4	0.8	0.0	TRG1	48414	48661	(110212)	+	BOV-A2	SINE/BovA	23	272	(0)	77
	1198	16.5	5.2	3.3	TRG1	48812	49080	(109793)	+	CHR-2A	SINE/tRNA-Glu	2	340	(4)	78
38	347	25.2	5.9	6.6	TRG1	49903	50055	(108818)	+	MIRb	SINE/MIR	109	256	(6)	79

	705	14.7	2.3	0.8	TRG1	50056	50185	(108688)	C	Bov-tA3	SINE/BovA	(1)	212	81	80
39	248	31.2	0.0	0.0	TRG1	50463	50526	(108347)	C	L2	LINE/L2	(16)	3403	3340	81
	1126	15.8	0.0	7.8	TRG1	50550	50768	(108105)	C	Bov-tA2	SINE/BovA	(3)	209	8	82
	201	3.9	0.0	0.0	TRG1	51442	51467	(107406)	+	(TTG)n	Simple_repeat	2	27	(0)	83
42	272	33.0	12.8	3.4	TRG1	51485	51719	(107154)	C	MIRb	SINE/MIR	(8)	260	4	84
43	243	33.6	9.7	0.7	TRG1	51793	51936	(106937)	+	MIRb	SINE/MIR	1	157	(111)	85
46	340	28.2	6.5	7.0	TRG1	53550	53806	(105067)	+	MIRm	SINE/MIR	1	263	(5)	86
	268	26.2	5.0	0.0	TRG1	53952	54031	(104842)	C	THER1_MD	SINE/MIR	(66)	208	125	87
	207	13.9	0.0	0.0	TRG1	54155	54190	(104683)	+	(CAAAA)n	Simple_repeat	2	37	(0)	88
	464	32.1	4.2	0.6	TRG1	54868	55033	(103840)	C	MIR	SINE/MIR	(71)	191	20	89
47	259	31.9	6.1	5.9	TRG1	55749	55933	(102940)	C	MIRb	SINE/MIR	(60)	202	31	90
	22	0.0	0.0	0.0	TRG1	56340	56361	(102512)	+	AT_rich	Low_complexity	1	22	(0)	91
	1019	19.6	1.3	0.9	TRG1	56362	56588	(102285)	C	L1_BT	LINE/L1	(0)	1161	934	92
	1322	14.4	0.0	0.2	TRG1	58092	58307	(100566)	C	Bov-tA2	SINE/BovA	(3)	209	1	93
	387	23.3	0.0	1.1	TRG1	59881	59967	(98906)	C	CHRL	SINE/tRNA-Glu	(82)	86	1	94
	345	25.3	2.5	9.3	TRG1	60651	60811	(98062)	+	L1M4	LINE/L1	4798	4947	(1199)	95
1	1299	13.2	0.5	0.5	TRG1	60897	61102	(97771)	+	Bov-tA2	SINE/BovA	7	212	(0)	96
2	196	21.4	2.4	0.0	TRG1	61198	61239	(97634)	+	MIRb	SINE/MIR	27	69	(199)	97
	363	20.3	0.0	0.0	TRG1	61272	61340	(97533)	C	Bov-tA2	SINE/BovA	(6)	206	138	98
	11388	4.6	4.4	4.4	TRG1	61436	62645	(96228)	C	BovB	LINE/RTE	(0)	3302	2039	99
	523	7.0	1.4	0.0	TRG1	62646	62716	(96157)	+	BTLTR1	LTR	44	115	(1083)	100
	11388	4.6	4.4	4.4	TRG1	62717	63060	(95813)	C	BovB	LINE/RTE	(1264)	2038	1680	99
	215	20.9	6.6	0.0	TRG1	63064	63121	(95752)	+	MIRb	SINE/MIR	102	163	(113)	101
	254	32.0	0.0	2.0	TRG1	63679	63780	(95093)	+	GA-rich	Low_complexity	1	100	(0)	102
	231	26.8	2.3	2.3	TRG1	63851	63938	(94935)	+	(CAGAGA)n	Simple_repeat	1	88	(0)	103
	207	10.0	0.0	0.0	TRG1	64010	64039	(94834)	+	(GA)n	Simple_repeat	1	30	(0)	104
	39	5.0	0.0	0.0	TRG1	64108	64167	(94706)	+	AT_rich	Low_complexity	1	60	(0)	105
	241	12.3	3.5	4.7	TRG1	64168	64252	(94621)	+	(TCTA)n	Simple_repeat	1	84	(0)	106
3	226	22.7	13.0	4.3	TRG1	64269	64360	(94513)	C	MLT1H	LTR/MaLR	(20)	529	430	107
4	399	31.9	2.2	0.7	TRG1	64415	64553	(94320)	C	MLT1H	LTR/MaLR	(207)	342	202	107
	380	16.0	0.0	5.1	TRG1	64558	64636	(94237)	C	Bov-tA2	SINE/BovA	(0)	212	138	108
	449	27.7	7.9	3.0	TRG1	65165	65253	(93620)	+	L1MD2	LINE/L1	120	213	(6334)	109
	207	0.0	0.0	0.0	TRG1	65254	65276	(93597)	+	(TTA)n	Simple_repeat	2	24	(0)	110
	449	27.7	7.9	3.0	TRG1	65277	65350	(93523)	+	L1MD2	LINE/L1	214	291	(6256)	109
5	806	30.5	5.9	2.1	TRG1	65385	65772	(93101)	+	L1MD2	LINE/L1	373	775	(5772)	109
7	638	9.8	0.0	0.0	TRG1	65866	65947	(92926)	+	Bov-tA2	SINE/BovA	7	88	(124)	111
	2021	9.3	0.7	1.5	TRG1	65950	66223	(92650)	+	BOV-A2	SINE/BovA	1	272	(0)	112
8	226	17.1	1.4	1.4	TRG1	66570	66640	(92233)	+	GA-rich	Low_complexity	2	72	(0)	113
	201	29.0	0.0	0.0	TRG1	66783	66844	(92029)	+	GA-rich	Low_complexity	2	63	(0)	114
	186	13.3	0.0	0.0	TRG1	66877	66906	(91967)	+	(TTTC)n	Simple_repeat	3	32	(0)	115
	346	23.3	5.5	1.1	TRG1	67147	67237	(91636)	C	CHRL	SINE/tRNA-Glu	(73)	95	1	116
9	3210	6.1	0.3	0.0	TRG1	67773	68163	(90710)	+	BovB	LINE/RTE	2911	3302	(0)	117
	448	16.7	0.0	0.0	TRG1	68259	68330	(90543)	+	Bov-tA3	SINE/BovA	141	212	(1)	118

10	2173	21.8	10.0	1.6	TRG1	68457	69007	(89866)	C	L1MA8	LINE/L1	(281)	6010	5414	119
	269	16.4	3.3	0.0	TRG1	69008	69068	(89805)	+	MER53	DNA	50	112	(81)	120
	1136	17.2	1.3	3.1	TRG1	71143	71370	(87503)	C	Bov-tA1	SINE/BovA	(3)	224	1	121
	234	0.0	0.0	0.0	TRG1	71380	71405	(87468)	+	(CTG)n	Simple_repeat	2	27	(0)	122
	1021	5.0	0.0	0.0	TRG1	71406	71525	(87348)	C	BOV-A2	SINE/BovA	(12)	260	141	123
11	221	30.7	6.9	6.2	TRG1	71562	71721	(87152)	+	MER5A	DNA/MER1_type	1	161	(28)	124
	406	26.8	1.0	3.0	TRG1	72184	72346	(86527)	+	L1M5	LINE/L1	4492	4646	(1541)	125
	1347	15.9	0.0	0.5	TRG1	72452	72653	(86220)	+	Bov-tA2	SINE/BovA	6	206	(6)	126
	216	29.4	4.5	6.0	TRG1	72860	72993	(85880)	C	MIR	SINE/MIR	(0)	262	131	127
	197	38.6	0.0	0.0	TRG1	73186	73255	(85618)	+	MIRb	SINE/MIR	26	95	(173)	128
	1940	7.9	4.8	0.0	TRG1	73316	73567	(85306)	+	BOV-A2	SINE/BovA	5	268	(4)	129
	2114	4.4	0.0	9.7	TRG1	74226	74525	(84348)	C	BOV-A2	SINE/BovA	(0)	272	2	130
	304	30.5	10.3	3.9	TRG1	74629	74873	(84000)	+	MIR	SINE/MIR	10	274	(0)	131
	423	0.0	0.0	0.0	TRG1	75961	76007	(82866)	+	(TG)n	Simple_repeat	1	47	(0)	132
	650	16.0	0.0	0.0	TRG1	76012	76111	(82762)	C	Bov-tA3	SINE/BovA	(10)	203	104	133
	2085	7.4	0.4	0.0	TRG1	76242	76512	(82361)	C	BOV-A2	SINE/BovA	(0)	272	1	134
	1122	5.6	0.7	0.0	TRG1	76513	76654	(82219)	C	BOV-A2	SINE/BovA	(129)	143	1	135
	250	32.8	10.3	4.0	TRG1	76729	76921	(81952)	+	MIR	SINE/MIR	1	203	(63)	136
	1500	13.2	0.0	0.0	TRG1	77092	77296	(81577)	C	Bov-tA3	SINE/BovA	(0)	213	9	137
	1398	15.4	0.8	3.4	TRG1	77416	77713	(81160)	C	L1_BT	LINE/L1	(27)	2605	919	138
	264	20.3	3.3	1.7	TRG1	78022	78081	(80792)	C	MER5B	DNA/MER1_type	(109)	69	9	139
	232	30.6	9.1	3.4	TRG1	81142	81317	(77556)	C	L1MA9	LINE/L1	(1)	6311	6126	140
2	240	31.5	10.1	2.4	TRG1	81506	81674	(77199)	+	MIR	SINE/MIR	2	183	(86)	141
6	319	27.1	6.6	3.4	TRG1	81912	82225	(76648)	+	L1MD2	LINE/L1	876	1201	(5333)	142
8	375	31.9	0.6	2.9	TRG1	82307	82477	(76396)	+	GA-rich	Low_complexity	1	167	(0)	143
10	1454	23.4	7.9	0.0	TRG1	82648	83002	(75871)	C	L1MA9	LINE/L1	(519)	5793	5411	140
	916	4.3	0.9	0.9	TRG1	84510	84625	(74248)	+	BOV-A2	SINE/BovA	1	116	(156)	144
	189	7.4	0.0	0.0	TRG1	84626	84652	(74221)	+	(CAG)n	Simple_repeat	2	28	(0)	145
11	249	20.0	3.9	3.9	TRG1	85344	85421	(73452)	+	MER5A	DNA/MER1_type	1	78	(111)	146
12	1031	11.9	9.4	0.0	TRG1	85500	85658	(73215)	C	Bov-tA2	SINE/BovA	(8)	204	31	147
14	367	29.4	0.8	0.8	TRG1	86796	86915	(71958)	+	MIR	SINE/MIR	117	236	(26)	148
	1165	12.0	9.1	1.1	TRG1	87194	87379	(71494)	+	Bov-tA2	SINE/BovA	4	204	(8)	149
	34	4.2	0.0	0.0	TRG1	88439	88486	(70387)	+	AT_rich	Low_complexity	1	48	(0)	150
	21	5.7	0.0	0.0	TRG1	88715	88749	(70124)	+	AT_rich	Low_complexity	1	35	(0)	151
	7688	4.5	0.9	0.1	TRG1	88813	89732	(69141)	+	BovB	LINE/RTE	2376	3302	(0)	152
	356	22.4	0.0	0.0	TRG1	89828	89894	(68979)	+	Bov-tA3	SINE/BovA	141	207	(6)	153
	728	26.5	3.1	3.1	TRG1	89965	90287	(68586)	+	L1MD	LINE/L1	2513	2835	(3311)	154
	1162	14.6	6.1	0.0	TRG1	90313	90513	(68360)	C	Bov-tA1	SINE/BovA	(25)	202	1	155
	1464	10.7	2.1	0.8	TRG1	90650	90869	(68004)	C	Bov-tA3	SINE/BovA	(0)	213	1	156
	207	29.1	11.0	6.2	TRG1	91079	91287	(67586)	C	MIR	SINE/MIR	(8)	254	36	157
	348	17.9	2.9	1.5	TRG1	91994	92061	(66812)	+	Bov-tA2	SINE/BovA	140	208	(4)	158
	229	36.4	8.9	2.9	TRG1	92821	93066	(65807)	+	MIR	SINE/MIR	2	262	(0)	159
	24	0.0	0.0	0.0	TRG1	93973	93996	(64877)	+	AT_rich	Low_complexity	1	24	(0)	160

	318	28.0	8.4	2.0	TRG1	94355	94516	(64357)	+	MIRb	SINE/MIR	1	177	(97)	161
	183	27.7	1.5	0.0	TRG1	94714	94778	(64095)	C	MIR	SINE/MIR	(67)	195	130	162
	207	0.0	0.0	0.0	TRG1	94882	94904	(63969)	+	(CTG)n	Simple_repeat	2	24	(0)	163
	279	10.3	0.0	0.0	TRG1	94905	94943	(63930)	C	BOV-A2	SINE/BovA	(12)	260	222	164
	384	16.7	10.8	5.0	TRG1	94966	95085	(63788)	C	MIRb	SINE/MIR	(140)	128	2	165
	1082	15.6	7.5	0.5	TRG1	95147	95333	(63540)	C	Bov-tA2	SINE/BovA	(6)	206	7	166
	22	8.8	0.0	0.0	TRG1	95349	95405	(63468)	+	AT_rich	Low_complexity	1	57	(0)	167
	225	23.7	3.0	10.6	TRG1	95431	95496	(63377)	C	MER5B	DNA/MER1_type	(109)	69	9	168
	1041	14.9	14.9	0.0	TRG1	95853	96026	(62847)	C	Bov-tA2	SINE/BovA	(8)	204	5	169
	554	23.9	0.8	0.0	TRG1	96027	96143	(62730)	C	Bov-tA2	SINE/BovA	(89)	123	6	170
	30	0.0	0.0	0.0	TRG1	96473	96502	(62371)	+	AT_rich	Low_complexity	1	30	(0)	171
	1261	12.8	1.1	0.5	TRG1	96630	96817	(62056)	+	Bov-tA2	SINE/BovA	19	207	(5)	172
	1195	15.5	1.0	1.5	TRG1	96947	97149	(61724)	C	Bov-tA2	SINE/BovA	(6)	206	5	173
9	4753	4.9	5.4	0.2	TRG1	98168	98756	(60117)	+	BovB	LINE/RTE	2683	3302	(0)	174
	439	14.9	0.0	0.0	TRG1	98852	98918	(59955)	+	Bov-tA3	SINE/BovA	141	207	(6)	175
	733	12.9	0.0	0.0	TRG1	100402	100517	(58356)	C	L1_Art	LINE/L1	(75)	2557	2442	176
	229	22.4	8.8	4.4	TRG1	100908	101066	(57807)	C	L1MA9	LINE/L1	(21)	6291	6126	177
9	2863	4.5	2.8	0.6	TRG1	101099	101453	(57420)	+	BovB	LINE/RTE	2940	3302	(0)	178
	376	20.0	0.0	0.0	TRG1	101549	101613	(57260)	+	Bov-tA3	SINE/BovA	141	205	(8)	179
	315	0.0	0.0	0.0	TRG1	101848	101882	(56991)	+	(TC)n	Simple_repeat	2	36	(0)	180
	253	10.0	2.0	2.0	TRG1	101883	101933	(56940)	+	(TA)n	Simple_repeat	1	51	(0)	181
10	1805	20.1	4.8	0.0	TRG1	101941	102298	(56575)	C	L1MA9	LINE/L1	(524)	5788	5414	177
	231	12.7	3.5	3.5	TRG1	102299	102355	(56518)	+	MER53	DNA	50	106	(87)	182
	249	21.7	1.7	0.0	TRG1	103898	103957	(54916)	+	CHR-2B	SINE/tRNA-Glu	1	61	(262)	183
	1239	16.7	1.4	0.0	TRG1	104666	104875	(53998)	+	Bov-tA1	SINE/BovA	6	218	(9)	184
11	392	32.2	4.3	2.2	TRG1	104944	105127	(53746)	+	MER5A	DNA/MER1_type	2	189	(0)	185
14	377	30.6	1.5	0.7	TRG1	106207	106341	(52532)	+	MIR	SINE/MIR	120	255	(7)	186
15	212	34.6	4.3	6.1	TRG1	106572	106734	(52139)	C	MIRb	SINE/MIR	(103)	165	6	187
	391	19.4	0.0	0.0	TRG1	107880	107946	(50927)	C	Bov-tA3	SINE/BovA	(6)	207	141	188
	423	14.9	1.3	1.3	TRG1	108042	108116	(50757)	C	BovB	LINE/RTE	(0)	3302	3228	189
	1951	6.3	3.6	0.0	TRG1	108128	108379	(50494)	C	BOV-A2	SINE/BovA	(1)	271	11	190
17	332	37.7	1.4	1.4	TRG1	108750	108897	(49976)	C	L1MD	LINE/L1	(1542)	4604	4457	191
	1009	12.6	0.0	1.3	TRG1	110339	110491	(48382)	C	Bov-tA2	SINE/BovA	(0)	212	62	192
	1217	12.0	0.6	0.6	TRG1	110988	111163	(47710)	C	Bov-tA3	SINE/BovA	(0)	213	38	193
	1316	16.0	0.0	0.2	TRG1	111350	111561	(47312)	+	Bov-tA2	SINE/BovA	1	204	(8)	194
19	197	31.8	10.6	5.3	TRG1	111626	111738	(47135)	C	L2	LINE/L2	(3)	3416	3298	195
20	343	31.8	12.6	3.5	TRG1	111957	112155	(46718)	C	MIRb	SINE/MIR	(25)	243	27	196
21	639	15.1	0.9	0.9	TRG1	112226	112332	(46541)	+	Bov-tA2	SINE/BovA	106	212	(0)	197
	367	20.0	0.0	2.6	TRG1	112939	113002	(45871)	+	Bov-tA2	SINE/BovA	138	201	(11)	198
	243	0.0	0.0	0.0	TRG1	113003	113029	(45844)	+	(AACTG)n	Simple_repeat	4	30	(0)	199
	367	20.0	0.0	2.6	TRG1	113030	113041	(45832)	+	Bov-tA2	SINE/BovA	202	212	(0)	198
	411	29.4	7.6	0.0	TRG1	113049	113167	(45706)	+	CHRL	SINE/tRNA-Glu	1	128	(40)	200
22	306	35.6	11.4	4.6	TRG1	113520	113887	(44986)	C	L4	LINE/RTE	(387)	1573	1181	201

	537	19.9	0.7	2.9	TRG1	114314	114456	(44417)	C	Bov-tA3	SINE/BovA	(0)	272	118	202
25	632	28.2	7.2	2.4	TRG1	115036	115242	(43631)	+	MIRb	SINE/MIR	25	241	(27)	203
31	192	28.7	12.9	0.0	TRG1	115706	115806	(43067)	+	MIRb	SINE/MIR	58	171	(97)	204
33	305	34.5	4.3	0.7	TRG1	116456	116595	(42278)	+	MIRb	SINE/MIR	107	251	(17)	205
	202	33.9	4.0	7.3	TRG1	117207	117330	(41543)	+	MIRb	SINE/MIR	97	216	(52)	206
35	871	10.4	0.8	2.2	TRG1	117787	117934	(40939)	+	Bov-tA2	SINE/BovA	88	252	(3)	207
36	1506	14.3	16.3	1.1	TRG1	118023	118298	(40575)	C	CHR-2B	SINE/tRNA-Glu	(4)	319	2	208
38	511	26.3	6.4	4.9	TRG1	118539	118742	(40131)	+	MIRb	SINE/MIR	60	266	(2)	209
	1148	17.5	0.6	0.0	TRG1	119515	119697	(39176)	+	Bov-tA2	SINE/BovA	29	212	(0)	210
41	1168	16.7	1.3	4.0	TRG1	119703	119927	(38946)	+	Bov-tA1	SINE/BovA	2	220	(7)	211
44	1355	12.2	2.5	1.0	TRG1	120239	120437	(38436)	+	Bov-tA2	SINE/BovA	5	206	(6)	212
45	349	34.9	7.4	3.7	TRG1	120906	121149	(37724)	+	MIR	SINE/MIR	10	262	(0)	213
46	259	32.8	5.1	0.0	TRG1	122458	122575	(36298)	+	MIR	SINE/MIR	1	122	(145)	214
47	186	27.3	1.5	0.0	TRG1	122805	122870	(36003)	C	MIR	SINE/MIR	(67)	195	129	215
	355	25.0	8.2	4.1	TRG1	122965	123110	(35763)	C	MIRb	SINE/MIR	(115)	153	2	216
	6646	10.4	1.3	0.8	TRG1	123473	124404	(34469)	+	BovB	LINE/RTE	2366	3302	(0)	217
	381	13.8	1.5	1.5	TRG1	124501	124566	(34307)	+	Bov-tA3	SINE/BovA	142	207	(6)	218
	245	18.2	0.0	0.0	TRG1	126529	126572	(32301)	C	tRNA-Glu-GAA	tRNA	(31)	44	1	219
	23	0.0	0.0	0.0	TRG1	126719	126741	(32132)	+	AT_rich	Low_complexity	1	23	(0)	220
1	2112	6.7	0.0	0.4	TRG1	127556	127823	(31050)	+	BOV-A2	SINE/BovA	1	267	(5)	221
2	208	28.2	7.3	5.3	TRG1	128108	128257	(30616)	+	MIRb	SINE/MIR	29	181	(87)	222
3	346	15.4	10.5	4.2	TRG1	129313	129407	(29466)	C	MLT1H	LTR/MaLR	(18)	466	366	223
4	351	32.1	9.7	0.0	TRG1	129461	129625	(29248)	C	MLT1H	LTR/MaLR	(196)	353	173	223
	1387	10.6	0.0	0.0	TRG1	129777	129975	(28898)	C	Bov-tA3	SINE/BovA	(2)	211	13	224
5	638	28.2	8.1	3.2	TRG1	130242	130589	(28284)	+	L1MD2	LINE/L1	420	784	(5763)	225
	989	4.8	0.7	4.8	TRG1	130590	130756	(28117)	C	BOV-A2	SINE/BovA	(12)	260	1	226
6	297	28.2	14.9	0.0	TRG1	130765	130945	(27928)	+	L1MD2	LINE/L1	817	1024	(5523)	225
7	376	15.9	0.0	0.0	TRG1	131044	131106	(27767)	+	Bov-tA2	SINE/BovA	2	64	(148)	227
8	341	33.3	0.0	2.2	TRG1	131162	131345	(27528)	+	GA-rich	Low_complexity	1	180	(0)	228
10	712	22.4	15.7	0.0	TRG1	131581	131803	(27070)	C	L1M2	LINE/L1	(512)	5788	5531	229
	1285	12.4	0.0	1.0	TRG1	131820	132022	(26851)	+	Bov-tA2	SINE/BovA	10	210	(2)	230
10	734	13.9	2.9	0.6	TRG1	132028	132179	(26694)	C	L1M2	LINE/L1	(776)	5536	5383	229
	389	17.9	0.0	0.0	TRG1	134326	134392	(24481)	+	Bov-tA3	SINE/BovA	141	207	(6)	231
12	1089	16.2	8.1	0.0	TRG1	134890	135074	(23799)	C	Bov-tA2	SINE/BovA	(8)	204	5	232
	649	17.6	0.0	0.0	TRG1	135075	135193	(23680)	C	Bov-tA2	SINE/BovA	(89)	123	5	233
13	1096	22.9	4.1	2.1	TRG1	135195	135630	(23243)	+	L1M2	LINE/L1	2942	3386	(2757)	234
	5797	15.3	1.8	0.3	TRG1	135653	136665	(22208)	+	BovB	LINE/RTE	2275	3302	(0)	235
	366	20.6	0.2	0.0	TRG1	136766	136840	(22033)	+	Bov-tA3	SINE/BovA	146	272	(0)	236
17	281	29.7	4.2	1.8	TRG1	137095	137262	(21611)	C	L1MC	LINE/L1	(1546)	4600	4429	237
	1346	10.9	2.5	1.0	TRG1	137525	137728	(21145)	C	Bov-tA2	SINE/BovA	(2)	210	4	238
	263	32.8	0.9	1.1	TRG1	138868	138897	(19976)	C	CHRL	SINE/tRNA-Glu	(0)	115	6	239
	274	32.0	0.0	1.3	TRG1	138898	138973	(19900)	C	tRNA-Glu-GAG_	tRNA	(0)	75	1	240
	404	13.5	6.7	0.0	TRG1	139542	139630	(19243)	C	L1_Art	LINE/L1	(59)	2573	2479	241

19	205	28.2	17.9	0.0	TRG1	139727	139804	(19069)	C	L2	LINE/L2	(1)	3418	3327	242	
	1096	18.1	2.5	1.5	TRG1	139964	140165	(18708)	C	Bov-tA2	SINE/BovA	(4)	208	5	243	
20	448	27.1	12.4	3.0	TRG1	140257	140443	(18430)	C	MIRb	SINE/MIR	(0)	262	56	244	
21	1086	16.0	8.2	0.0	TRG1	140445	140638	(18235)	+	Bov-tA2	SINE/BovA	2	211	(1)	245	
22	326	38.1	0.6	5.5	TRG1	141347	141510	(17363)	C	L4	LINE/RTE	(613)	1347	1192	246	
	259	29.0	5.2	6.3	TRG1	141947	142091	(16782)	C	MIR	SINE/MIR	(105)	163	18	247	
	571	12.2	1.1	0.0	TRG1	142428	142517	(16356)	+	Bov-tA2	SINE/BovA	116	206	(6)	248	
25	681	30.4	7.0	0.0	TRG1	142637	142850	(16023)	+	MIRb	SINE/MIR	26	254	(14)	249	
27	401	17.1	1.3	0.0	TRG1	143636	143711	(15162)	+	CHR-2B	SINE/tRNA-Glu	1	77	(246)	250	
	1200	18.4	1.4	0.0	TRG1	143937	144154	(14719)	C	Bov-tA1	SINE/BovA	(6)	221	1	251	
	207	6.9	0.0	0.0	TRG1	144251	144279	(14594)	+	(TG)n	Simple_repeat	2	30	(0)	252	
28	458	18.4	0.0	2.0	TRG1	144313	144412	(14461)	C	L1_Art	LINE/L1	(218)	2414	2317	253	
31	246	28.5	9.4	1.4	TRG1	145204	145300	(13573)	+	MIRb	SINE/MIR	64	167	(107)	254	
	1201	15.2	0.6	18.4	TRG1	145367	145581	(13292)	C	BOV-A2	SINE/BovA	(0)	272	95	255	
	216	0.0	0.0	0.0	TRG1	145582	145605	(13268)	+	(CTG)n	Simple_repeat	3	26	(0)	256	
	1201	15.2	0.6	18.4	TRG1	145606	145720	(13153)	C	BOV-A2	SINE/BovA	(178)	94	1	255	
	21	0.0	0.0	0.0	TRG1	145778	145798	(13075)	+	AT_rich	Low_complexity	1	21	(0)	257	
33	191	39.8	4.7	0.8	TRG1	146339	146467	(12406)	+	MIRb	SINE/MIR	107	240	(28)	258	
	358	15.9	0.0	0.0	TRG1	147174	147236	(11637)	C	Bov-tA3	SINE/BovA	(10)	203	141	259	
	1845	7.5	0.0	0.0	TRG1	147332	147583	(11290)	C	BovB	LINE/RTE	(0)	3302	3051	260	
34	200	37.4	9.5	1.9	TRG1	148057	148266	(10607)	C	L4	LINE/RTE	(313)	1647	1422	261	
36	1615	16.4	11.8	0.3	TRG1	148404	148709	(10164)	C	CHR-2	SINE/tRNA-Glu	(3)	341	1	262	
38	322	26.5	7.4	8.0	TRG1	149331	149506	(9367)	+	MIRb	SINE/MIR	60	234	(34)	263	
39	262	26.7	0.0	1.5	TRG1	149801	149875	(8998)	C	L2	LINE/L2	(11)	3408	3294	264	
	206	21.9	1.6	0.0	TRG1	150394	150457	(8416)	+	(TA)n	Simple_repeat	2	66	(0)	265	
42	312	27.5	9.2	3.2	TRG1	151235	151441	(7432)	C	MIR_Mars	SINE/MIR	(19)	249	34	266	
	197	32.8	6.7	8.1	TRG1	151606	151754	(7119)	+	MIRb	SINE/MIR	99	245	(23)	267	
43	424	31.6	7.1	0.0	TRG1	152408	152562	(6311)	+	MIR	SINE/MIR	2	167	(95)	268	
	1132	13.7	0.5	2.8	TRG1	152570	152780	(6093)	C	Bov-tA2	SINE/BovA	(0)	212	7	269	
46	369	29.7	3.5	1.0	TRG1	154196	154363	(4510)	+	MIR	SINE/MIR	1	172	(104)	270	
	1194	19.3	0.5	0.0	TRG1	155602	155803	(3070)	C	Bov-tA2	SINE/BovA	(6)	206	4	271	
	330	25.0	0.0	0.0	TRG1	155824	155899	(2974)	+	Bov-tA2	SINE/BovA	135	210	(2)	272	
	404	16.4	0.0	0.0	TRG1	155993	156059	(2814)	C	Bov-tA3	SINE/BovA	(6)	207	141	273	
	4177	5.3	2.8	0.2	TRG1	156155	156717	(2156)	C	BovB	LINE/RTE	(0)	3302	2725	274	
	251	25.0	1.5	0.0	TRG1	156717	156784	(2089)	C	L1_SS	LINE/L1	(538)	6280	6212	275	*
	806	14.4	0.0	0.0	TRG1	156799	156930	(1943)	C	Bov-tA3	SINE/BovA	(10)	203	72	276	
	745	28.7	6.3	6.8	TRG1	156955	157365	(1508)	+	L1_Art	LINE/L1	2130	2538	(94)	277	
	1459	13.6	3.1	3.5	TRG1	157379	157637	(1236)	C	BOV-A2	SINE/BovA	(13)	259	2	278	
	2128	7.0	0.0	0.4	TRG1	157642	157914	(959)	C	BOV-A2	SINE/BovA	(0)	272	1	279	
	1171	13.1	0.0	0.6	TRG1	158483	158659	(214)	+	Bov-tA2	SINE/BovA	31	206	(6)	280	

d)

	SW score[a]	perc div.[b]	perc del.[c]	perc ins.[d]	query sequence	position in query[e] begin	end	(left)[f]		matching repeat[g]	repeat class/family	position in repeat[h] begin	end	(left)[i]	ID	
1	1276	16.0	1.0	1.0	TRG2ov	2420	2627	(92465)	+	Bov-tA3	SINE/BovA	6	213	(0)	1	
2	194	21.5	20.0	5.2	TRG2ov	3593	3729	(91363)	+	MIR	SINE/MIR	16	165	(97)	2	
6	252	26.6	20.8	0.6	TRG2ov	3980	4138	(90954)	+	L1MD2	LINE/L1	874	1064	(5483)	3	*
9	4125	6.8	0.4	0.0	TRG2ov	4124	4640	(90452)	+	BovB	LINE/RTE	2784	3302	(0)	4	
	343	22.4	0.0	0.0	TRG2ov	4738	4804	(90288)	+	Bov-tA3	SINE/BovA	141	207	(6)	5	
6	382	28.4	5.8	2.9	TRG2ov	4809	4982	(90110)	+	L1MD2	LINE/L1	1038	1216	(5331)	3	
8	365	27.6	1.4	5.3	TRG2ov	5004	5320	(89772)	+	GA-rich	Low_complexity	1	304	(0)	6	
10	1505	18.9	5.1	0.6	TRG2ov	5419	5782	(89310)	C	L1M2	LINE/L1	(504)	5793	5411	7	
12	1267	16.7	0.5	3.3	TRG2ov	7553	7763	(87329)	C	Bov-tA2	SINE/BovA	(6)	206	2	8	
11	263	24.0	5.9	1.0	TRG2ov	8130	8230	(86862)	+	MER5A	DNA/MER1_type	1	106	(83)	9	
13	1504	22.3	4.5	4.3	TRG2ov	8934	9509	(85583)	+	L1M2	LINE/L1	2840	3416	(2727)	10	
	4874	24.2	3.4	2.1	TRG2ov	9525	10839	(84253)	+	L1_SS	LINE/L1	5468	6798	(20)	11	
	1331	11.9	0.5	1.5	TRG2ov	10993	11189	(83903)	+	Bov-tA2	SINE/BovA	9	203	(9)	12	
	21	0.0	0.0	0.0	TRG2ov	11349	11369	(83723)	+	AT_rich	Low_complexity	1	21	(0)	13	
16	37	7.6	0.0	0.0	TRG2ov	11479	11557	(83535)	+	AT_rich	Low_complexity	1	79	(0)	14	
17	301	31.2	2.4	2.4	TRG2ov	11614	11777	(83315)	C	L1M5	LINE/L1	(1544)	4602	4439	15	
	228	24.1	3.4	6.7	TRG2ov	12035	12123	(82969)	C	CHR-2B	SINE/tRNA-Glu	(237)	86	1	16	
18	675	15.2	11.9	0.8	TRG2ov	13051	13176	(81916)	+	Bov-tA3	SINE/BovA	68	207	(6)	17	
19	276	26.9	11.3	4.0	TRG2ov	14124	14247	(80845)	C	L2	LINE/L2	(1)	3418	3286	18	
20	464	30.6	11.0	1.7	TRG2ov	14424	14596	(80496)	C	MIRb	SINE/MIR	(17)	251	63	19	
22	257	37.0	11.7	3.6	TRG2ov	14908	15412	(79680)	C	L4	LINE/RTE	(230)	1730	1185	20	
25	585	24.1	12.0	2.6	TRG2ov	16115	16306	(78786)	+	MIRb	SINE/MIR	25	234	(34)	21	
26	615	10.3	0.0	0.0	TRG2ov	16308	16394	(78698)	+	Bov-tA2	SINE/BovA	114	200	(12)	22	
27	423	17.4	2.5	0.1	TRG2ov	17237	17345	(77747)	+	CHRL	SINE/tRNA-Glu	1	115	(0)	23	
28	424	25.5	0.0	0.0	TRG2ov	17677	17774	(77318)	C	L1_Art	LINE/L1	(218)	2414	2317	24	
29	273	30.8	0.0	0.0	TRG2ov	18357	18421	(76671)	+	CHRL	SINE/tRNA-Glu	1	65	(103)	25	
30	422	10.7	2.6	2.5	TRG2ov	18473	18558	(76534)	+	Bov-tA3	SINE/BovA	135	224	(3)	26	
31	230	21.4	6.7	7.9	TRG2ov	18585	18711	(76381)	+	MIRb	SINE/MIR	40	172	(96)	27	
33	337	33.8	3.0	2.3	TRG2ov	19358	19490	(75602)	+	MIRb	SINE/MIR	114	247	(21)	28	
34	246	29.2	16.3	7.3	TRG2ov	20614	20901	(74191)	C	L4	LINE/RTE	(240)	1720	1407	29	
35	1418	16.4	0.0	0.0	TRG2ov	21095	21302	(73790)	+	Bov-tA2	SINE/BovA	2	209	(3)	30	
38	410	22.4	5.5	7.9	TRG2ov	21858	22022	(73070)	+	MIRb	SINE/MIR	60	220	(48)	31	
40	249	33.8	0.0	0.0	TRG2ov	22299	22375	(72717)	C	MIRm	SINE/MIR	(9)	267	191	32	
	213	11.5	7.7	0.0	TRG2ov	22560	22637	(72455)	+	(TA)n	Simple_repeat	2	85	(0)	33	
	1180	17.6	0.5	0.0	TRG2ov	22667	22870	(72222)	C	Bov-tA1	SINE/BovA	(3)	224	20	34	
42	333	31.8	5.7	3.7	TRG2ov	23562	23797	(71295)	C	MIRb	SINE/MIR	(2)	266	15	35	
43	225	37.2	4.0	2.6	TRG2ov	23869	24020	(71072)	+	MIRb	SINE/MIR	16	169	(99)	36	
44	2044	7.6	2.3	0.0	TRG2ov	24033	24295	(70797)	+	BOV-A2	SINE/BovA	2	270	(2)	37	
45	497	32.3	5.6	3.4	TRG2ov	25021	25254	(69838)	+	MIR	SINE/MIR	10	248	(14)	38	

46	417	27.5	6.6	1.8	TRG2ov	26444	26630	(68462)	+	MIR	SINE/MIR	4	196	(69)	39	
	338	21.2	10.7	0.0	TRG2ov	27098	27195	(67897)	C	L1_BT	LINE/L1	(71)	2561	1070	40	
	1019	12.9	0.0	0.0	TRG2ov	27629	27767	(67325)	C	Bov-tA2	SINE/BovA	(70)	142	4	41	
	368	19.3	7.2	0.0	TRG2ov	27915	27997	(67095)	C	L1M2	LINE/L1	(620)	5692	5604	42	*
	747	28.9	5.6	4.0	TRG2ov	27992	28437	(66655)	+	L1MA7	LINE/L1	2131	5941	(59)	43	
	1589	8.8	1.8	0.9	TRG2ov	28945	29161	(65931)	C	Bov-tA1	SINE/BovA	(8)	219	1	44	
1	1401	15.0	1.0	0.0	TRG2ov	31031	31230	(63862)	+	Bov-tA2	SINE/BovA	3	204	(8)	45	
9	5274	6.1	0.6	0.1	TRG2ov	31471	32128	(62964)	+	BovB	LINE/RTE	2642	3302	(0)	46	
	396	14.1	0.0	0.0	TRG2ov	32222	32285	(62807)	+	Bov-tA3	SINE/BovA	141	204	(9)	47	
	198	0.0	0.0	0.0	TRG2ov	32286	32307	(62785)	+	(AACTG)n	Simple_repeat	3	24	(0)	48	
2	288	24.5	14.1	0.9	TRG2ov	33045	33179	(61913)	+	MIR3	SINE/MIR	13	165	(96)	49	
6	229	29.9	5.9	3.0	TRG2ov	33564	33732	(61360)	+	L1MDb	LINE/L1	899	1072	(5337)	50	
8	396	30.6	0.7	4.7	TRG2ov	33749	34091	(61001)	+	GA-rich	Low_complexity	1	329	(0)	51	
10	1384	19.8	4.6	0.3	TRG2ov	34227	34557	(60535)	C	L1M2	LINE/L1	(465)	6353	5411	52	
12	1180	15.7	0.5	3.3	TRG2ov	36389	36599	(58493)	C	Bov-tA2	SINE/BovA	(6)	206	2	53	
11	229	24.0	5.9	1.0	TRG2ov	36964	37064	(58028)	+	MER5A	DNA/MER1_type	1	106	(83)	54	
13	1293	22.8	3.9	4.3	TRG2ov	37775	38334	(56758)	+	L1M2	LINE/L1	2850	3407	(2736)	55	
	2352	20.7	2.5	1.2	TRG2ov	38359	38954	(56138)	+	L1_SS	LINE/L1	5468	6071	(747)	56	
	948	27.7	3.4	4.3	TRG2ov	38964	39444	(55648)	+	L1_SS	LINE/L1	2074	6706	(112)	57	
	1161	13.5	0.5	1.5	TRG2ov	39538	39732	(55360)	+	Bov-tA2	SINE/BovA	11	203	(9)	58	
	21	0.0	0.0	0.0	TRG2ov	39891	39911	(55181)	+	AT_rich	Low_complexity	1	21	(0)	59	
16	39	8.0	0.0	0.0	TRG2ov	40013	40100	(54992)	+	AT_rich	Low_complexity	1	88	(0)	60	
17	282	20.7	8.4	1.2	TRG2ov	40190	40272	(54820)	C	L1M5	LINE/L1	(1577)	4569	4481	61	
18	634	15.2	11.9	0.8	TRG2ov	41600	41725	(53367)	+	Bov-tA3	SINE/BovA	68	207	(6)	62	
19	282	27.3	9.7	2.4	TRG2ov	42675	42798	(52294)	C	L2	LINE/L2	(1)	3418	3286	63	
20	451	30.7	0.0	1.7	TRG2ov	43053	43168	(51924)	C	MIRb	SINE/MIR	(112)	156	43	64	
22	228	39.8	3.4	0.0	TRG2ov	43854	43971	(51121)	C	L4	LINE/RTE	(654)	1306	1185	65	
	799	12.8	0.0	2.9	TRG2ov	44451	44587	(50505)	+	BOV-A2	SINE/BovA	5	137	(135)	66	
25	595	24.1	12.0	2.6	TRG2ov	45144	45335	(49757)	+	MIRb	SINE/MIR	25	234	(34)	67	
26	504	14.9	0.0	0.0	TRG2ov	45337	45423	(49669)	+	Bov-tA2	SINE/BovA	114	200	(12)	68	
27	397	18.0	2.5	0.1	TRG2ov	46268	46376	(48716)	+	CHRL	SINE/tRNA-Glu	1	115	(0)	69	
29	289	29.2	0.0	0.0	TRG2ov	47379	47443	(47649)	+	CHRL	SINE/tRNA-Glu	1	65	(103)	70	
30	470	12.8	1.1	1.1	TRG2ov	47511	47597	(47495)	+	Bov-tA1	SINE/BovA	138	224	(3)	71	
31	242	18.5	6.7	10.0	TRG2ov	47624	47713	(47379)	+	MIRm	SINE/MIR	40	126	(150)	72	
33	341	31.4	3.1	2.4	TRG2ov	48387	48513	(46579)	+	MIRb	SINE/MIR	109	236	(32)	73	
34	278	28.9	16.7	7.3	TRG2ov	49643	49929	(45163)	C	L4	LINE/RTE	(240)	1720	1407	74	
35	1273	16.7	0.5	0.0	TRG2ov	50127	50330	(44762)	+	Bov-tA2	SINE/BovA	5	209	(3)	75	
38	394	24.5	4.3	6.8	TRG2ov	50889	51050	(44042)	+	MIRb	SINE/MIR	63	220	(48)	76	
37	2197	3.7	1.1	0.0	TRG2ov	51272	51539	(43553)	+	BOV-A2	SINE/BovA	2	272	(0)	77	
40	270	32.5	0.0	0.0	TRG2ov	51613	51689	(43403)	C	MIRm	SINE/MIR	(9)	267	191	78	
	194	17.4	2.2	0.0	TRG2ov	51870	51915	(43177)	+	(TA)n	Simple_repeat	1	47	(0)	79	
	1006	19.6	0.5	0.0	TRG2ov	51945	52148	(42944)	C	Bov-tA1	SINE/BovA	(3)	224	20	80	
	204	21.1	1.9	1.9	TRG2ov	52475	52527	(42565)	+	(TA)n	Simple_repeat	2	54	(0)	81	

	506	34.1	5.1	1.3	TRG2ov	52803	53037	(42055)	+	L1M5	LINE/L1	2409	2652	(3494)	82
	665	12.6	1.8	1.8	TRG2ov	53578	53690	(41402)	+	Bov-tA2	SINE/BovA	97	209	(3)	83
	192	25.0	0.0	2.0	TRG2ov	53816	53864	(41228)	+	MER5B	DNA/MER1_type	21	68	(110)	84
	29	2.8	0.0	0.0	TRG2ov	54102	54137	(40955)	+	AT_rich	Low_complexity	1	36	(0)	85
42	396	32.1	8.1	3.1	TRG2ov	54772	55015	(40077)	C	MIRb	SINE/MIR	(2)	266	10	86
43	201	36.7	5.0	1.4	TRG2ov	55079	55231	(39861)	+	MIR	SINE/MIR	16	169	(96)	87
44	2088	6.0	1.1	0.0	TRG2ov	55243	55508	(39584)	+	BOV-A2	SINE/BovA	2	270	(2)	88
45	496	33.2	6.0	4.0	TRG2ov	56236	56483	(38609)	+	MIR	SINE/MIR	10	262	(0)	89
46	410	30.1	6.4	0.7	TRG2ov	57665	57851	(37241)	+	MIR	SINE/MIR	4	196	(69)	90
	1375	14.2	0.5	0.0	TRG2ov	58785	58989	(36103)	C	Bov-tA2	SINE/BovA	(3)	209	4	91
	502	19.5	5.1	0.0	TRG2ov	59129	59246	(35846)	C	L1M2	LINE/L1	(585)	5727	5604	92
	630	28.1	7.5	6.2	TRG2ov	59253	59689	(35403)	+	L1_Art	LINE/L1	2131	2573	(59)	93
	1419	9.7	1.8	0.5	TRG2ov	60196	60412	(34680)	C	Bov-tA1	SINE/BovA	(7)	220	1	94
	21	0.0	0.0	0.0	TRG2ov	61801	61821	(33271)	+	AT_rich	Low_complexity	1	21	(0)	95
	639	13.8	2.8	0.0	TRG2ov	61923	62031	(33061)	+	Bov-tA3	SINE/BovA	92	203	(10)	96
	2011	5.8	4.6	0.0	TRG2ov	62388	62646	(32446)	+	BOV-A2	SINE/BovA	1	271	(1)	97
	198	0.0	0.0	0.0	TRG2ov	63601	63622	(31470)	+	(CA)n	Simple_repeat	2	23	(0)	98
	1329	18.7	0.9	0.0	TRG2ov	63710	63928	(31164)	+	Bov-tA1	SINE/BovA	4	224	(3)	99
	193	31.0	19.1	1.4	TRG2ov	63957	64103	(30989)	C	MIRb	SINE/MIR	(10)	258	86	100
	181	26.5	16.7	7.8	TRG2ov	64262	64351	(30741)	+	MIRb	SINE/MIR	17	114	(154)	101
	434	18.1	0.0	11.8	TRG2ov	64389	64423	(30669)	+	L1_Art	LINE/L1	2317	2348	(284)	102
	1283	14.0	1.0	0.5	TRG2ov	64424	64624	(30468)	+	Bov-tA2	SINE/BovA	5	206	(6)	103
	434	18.1	0.0	11.8	TRG2ov	64625	64707	(30385)	+	L1_Art	LINE/L1	2349	2421	(211)	102
	378	0.0	0.0	0.0	TRG2ov	64726	64767	(30325)	+	(TA)n	Simple_repeat	2	43	(0)	104
	192	12.5	0.0	0.0	TRG2ov	64820	64851	(30241)	+	(TAAAA)n	Simple_repeat	5	36	(0)	105
	291	27.6	7.1	1.0	TRG2ov	64852	64950	(30142)	+	MIRb	SINE/MIR	123	227	(41)	106
	867	23.4	7.5	6.6	TRG2ov	65965	66284	(28808)	+	MER33	DNA/MER1_type	2	324	(0)	107
	1475	24.5	1.6	3.8	TRG2ov	66661	67106	(27986)	+	L1_SS	LINE/L1	6286	6721	(97)	108
	276	28.8	2.3	1.1	TRG2ov	67902	67995	(27097)	+	MIR	SINE/MIR	56	149	(67)	109
	208	33.3	2.1	6.5	TRG2ov	68065	68157	(26935)	C	L3_Mars	LINE/CR1	(297)	4125	4037	110
	2705	24.2	3.3	2.0	TRG2ov	69684	70443	(24649)	+	L1_SS	LINE/L1	5998	6767	(51)	111
	397	16.4	0.0	0.0	TRG2ov	70459	70525	(24567)	C	Bov-tA3	SINE/BovA	(6)	207	141	112
	1255	5.3	0.0	0.0	TRG2ov	70622	70772	(24320)	C	BovB	LINE/RTE	(0)	3302	3152	113
	2424	4.8	0.0	0.0	TRG2ov	71341	71612	(23480)	C	BOV-A2	SINE/BovA	(0)	272	1	114
19	316	29.3	10.7	4.9	TRG2ov	72618	72739	(22353)	C	L2	LINE/L2	(2)	3376	3248	115
	1145	3.8	0.0	0.0	TRG2ov	72984	73116	(21976)	+	BOV-A2	SINE/BovA	133	265	(7)	116
20	383	21.2	1.2	3.6	TRG2ov	73172	73254	(21838)	C	MIRb	SINE/MIR	(115)	153	73	117
22	327	37.0	10.1	5.2	TRG2ov	73675	74190	(20902)	C	L4	LINE/RTE	(235)	1725	1185	118
23	1504	11.0	0.0	0.1	TRG2ov	74250	74470	(20622)	+	Bov-tA3	SINE/BovA	1	213	(0)	119
24	21	3.6	0.0	0.0	TRG2ov	74751	74778	(20314)	+	AT_rich	Low_complexity	1	28	(0)	120
	1037	22.9	8.3	8.4	TRG2ov	75137	75613	(19479)	+	L1_Art	LINE/L1	2131	6804	(69)	121
	1453	19.9	3.6	0.9	TRG2ov	75730	76058	(19034)	C	CHR-2	SINE/tRNA-Glu	(4)	340	3	122
31	326	22.1	10.5	0.0	TRG2ov	76197	76282	(18810)	+	MIRb	SINE/MIR	65	159	(109)	123

32	954	17.2	8.4	2.6	TRG2ov	76798	76988	(18104)	+	Bov-tA2	SINE/BovA	5	206	(6)	124	
33	322	28.1	7.2	3.2	TRG2ov	77172	77296	(17796)	+	MIRb	SINE/MIR	111	240	(28)	125	
34	183	37.9	6.8	0.7	TRG2ov	78548	78693	(16399)	C	L4	LINE/RTE	(204)	1756	1602	126	
	945	12.3	0.0	0.0	TRG2ov	78737	78866	(16226)	C	Bov-tA2	SINE/BovA	(23)	189	60	127	
35	1442	14.3	1.0	0.0	TRG2ov	78938	79140	(15952)	+	Bov-tA2	SINE/BovA	5	209	(3)	128	
	331	20.2	0.2	1.4	TRG2ov	79161	79239	(15853)	C	BOV-A2	SINE/BovA	(2)	270	49	129	
	7644	13.2	1.5	2.5	TRG2ov	79329	80538	(14554)	C	BovB	LINE/RTE	(4)	3298	2101	130	
	477	11.6	1.4	0.0	TRG2ov	80548	80616	(14476)	+	BTLTR1	LTR	45	114	(1084)	131	
36	1014	20.7	24.8	0.0	TRG2ov	80840	81085	(14007)	C	CHR-2B	SINE/tRNA-Glu	(16)	307	1	132	
	202	30.6	2.9	6.7	TRG2ov	81555	81659	(13433)	C	MIRb	SINE/MIR	(0)	268	168	133	*
38	375	28.9	8.5	5.5	TRG2ov	81655	81855	(13237)	+	MIRb	SINE/MIR	60	266	(2)	134	
	240	21.0	2.4	1.2	TRG2ov	82257	82338	(12754)	+	(CATA)n	Simple_repeat	3	85	(0)	135	
41	1429	15.8	0.9	0.0	TRG2ov	82482	82702	(12390)	+	Bov-tA1	SINE/BovA	1	223	(4)	136	
	551	7.4	1.2	0.0	TRG2ov	82861	82941	(12151)	+	(TA)n	Simple_repeat	1	82	(0)	137	
	233	23.9	0.0	4.3	TRG2ov	83218	83287	(11805)	+	(TA)n	Simple_repeat	1	67	(0)	138	
	189	0.0	0.0	0.0	TRG2ov	83904	83924	(11168)	+	(CAGTT)n	Simple_repeat	4	24	(0)	139	
	296	28.1	0.0	0.0	TRG2ov	83925	83988	(11104)	C	Bov-tA3	SINE/BovA	(9)	204	141	140	
	2240	6.3	0.0	0.0	TRG2ov	84084	84352	(10740)	C	BovB	LINE/RTE	(0)	3302	3034	141	
42	300	25.7	11.9	0.0	TRG2ov	84362	84462	(10630)	C	MIRm	SINE/MIR	(124)	152	40	142	
44	1491	13.6	0.0	0.0	TRG2ov	84676	84881	(10211)	+	Bov-tA2	SINE/BovA	4	209	(3)	143	
45	627	32.4	4.8	2.8	TRG2ov	85571	85818	(9274)	+	MIR	SINE/MIR	10	262	(0)	144	
	1617	9.6	0.0	0.5	TRG2ov	86098	86306	(8786)	+	Bov-tA2	SINE/BovA	5	212	(0)	145	
46	343	29.0	3.8	2.4	TRG2ov	87350	87529	(7563)	+	MIR	SINE/MIR	1	180	(96)	146	
	202	32.7	14.6	3.2	TRG2ov	87689	87846	(7246)	C	MIRb	SINE/MIR	(70)	198	23	147	
47	341	27.0	12.6	0.0	TRG2ov	88565	88675	(6417)	C	MIR	SINE/MIR	(55)	207	83	148	
	425	17.5	0.1	18.6	TRG2ov	88721	88836	(6256)	+	Bov-tA1	SINE/BovA	135	263	(3)	149	
	262	21.8	0.0	0.0	TRG2ov	88857	88911	(6181)	+	Bov-tA1	SINE/BovA	168	222	(5)	150	
	366	6.1	0.0	0.0	TRG2ov	88912	88960	(6132)	+	(CA)n	Simple_repeat	1	49	(0)	151	
	347	26.0	1.3	1.3	TRG2ov	88970	89047	(6045)	C	MIR	SINE/MIR	(178)	84	7	148	
	729	27.0	6.9	6.9	TRG2ov	89089	89434	(5658)	+	L1_Art	LINE/L1	2234	2579	(53)	152	
	314	32.5	10.7	3.0	TRG2ov	90176	90372	(4720)	C	MIRb	SINE/MIR	(48)	220	9	153	
	188	21.8	6.9	5.2	TRG2ov	90479	90536	(4556)	+	MER5B	DNA/MER1_type	112	170	(8)	154	
	439	23.5	15.1	2.1	TRG2ov	91084	91322	(3770)	C	L1MC4a	LINE/L1	(1836)	5972	5703	155	
	428	16.9	0.0	0.0	TRG2ov	91395	91465	(3627)	C	Bov-tA2	SINE/BovA	(4)	208	138	156	
	921	13.8	0.0	0.7	TRG2ov	91562	91700	(3392)	C	BovB	LINE/RTE	(1)	3301	3164	157	
	548	23.1	0.0	7.1	TRG2ov	91769	91936	(3156)	C	L1MC4a	LINE/L1	(2339)	5469	5314	155	
	396	22.2	4.0	6.4	TRG2ov	94913	95085	(7)	C	L1MA9	LINE/L1	(10)	6302	6134	158	

[a] Smith-Waterman score of the match. [b] % substitutions in matching region compared to the consensus. [c] % of bases opposite a gap in the query sequence-deleted bp. [d] % of bases opposite a gap in the repeat consensus-inserted bp. [e] starting, ending position of match in query sequence. [f] no. of bases in query sequence past the ending position of match. [g] C or + if match is or isn't with the complement of the consensus sequence in the database. [h] starting, ending position of match in database sequence. [i] no. of bases in (complement of) the repeat consensus sequence prior to beginning of the match. A number in the first column identify shared repeats listed in Tab. 2. an asterisk (*) in the final column indicates that there is a higher-scoring match whose domain partly (<80%) includes the domain of this match.
