# Supplementary figures and images for: Genomic organization and recombinational unit duplication-driven evolution of ovine and bovine T cell receptor gamma loci
Source: BMC Genomics. 2008 Feb 18;9:81. doi: 10.1186/1471-2164-9-81 (PMC2270265; doi:10.1186/1471-2164-9-81)

Genomic comparison of ovine and bovine TRG1 loci

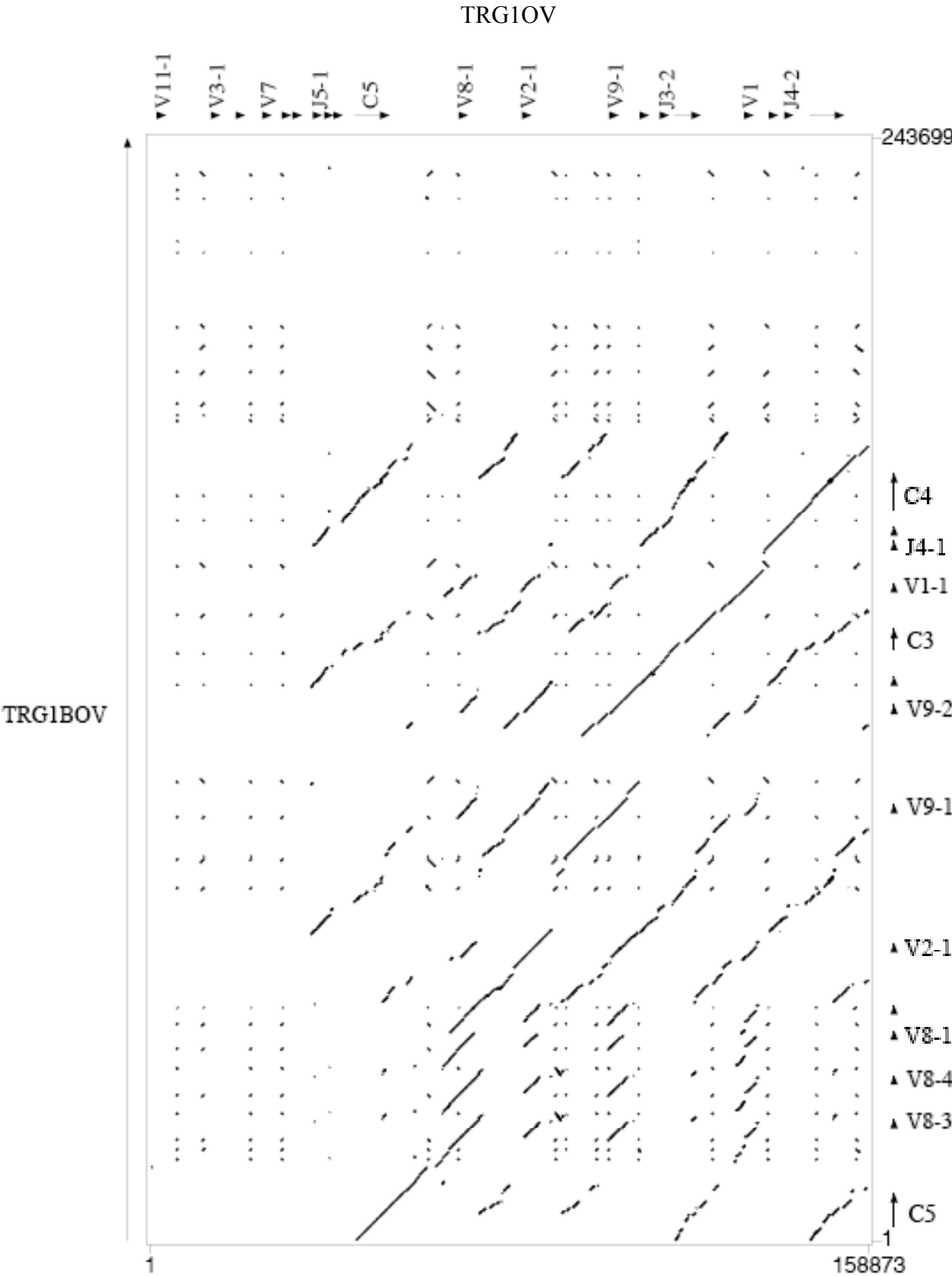

Supplement: Additional File 4 — Figure S4 – Genomic comparison of ovine and bovine TRG1 loci. The alignment of ovine TRG1 locus and bovine TRG1 locus [GenBank: AY644517] was visualized as a dotplot matrix obtained with the PipMaker program. The gene transcriptional orientation is indicated by arrows. [file 1471-2164-9-81-S4.pdf]

Genomic comparison of ovine and human TRG loci

a)

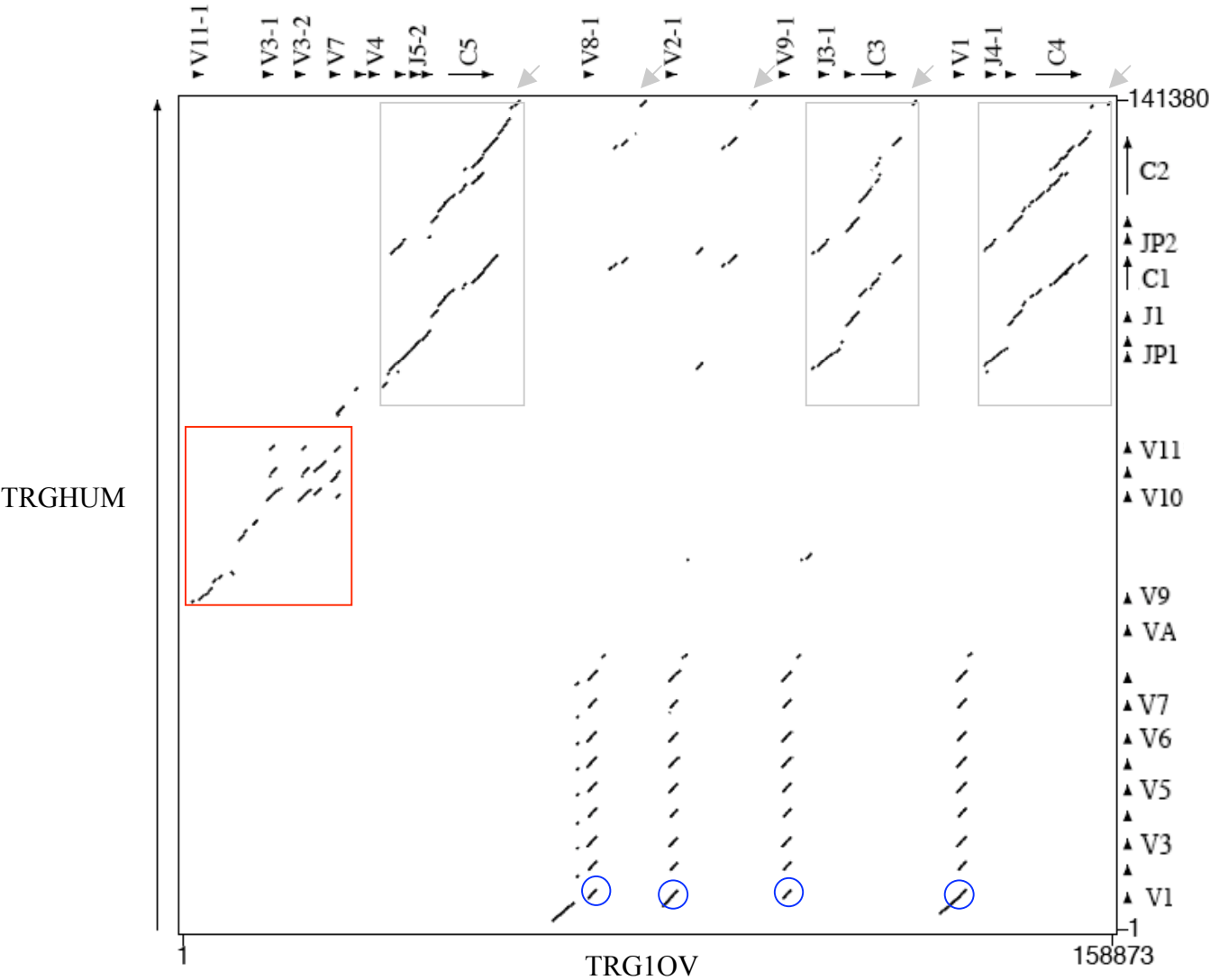

b)

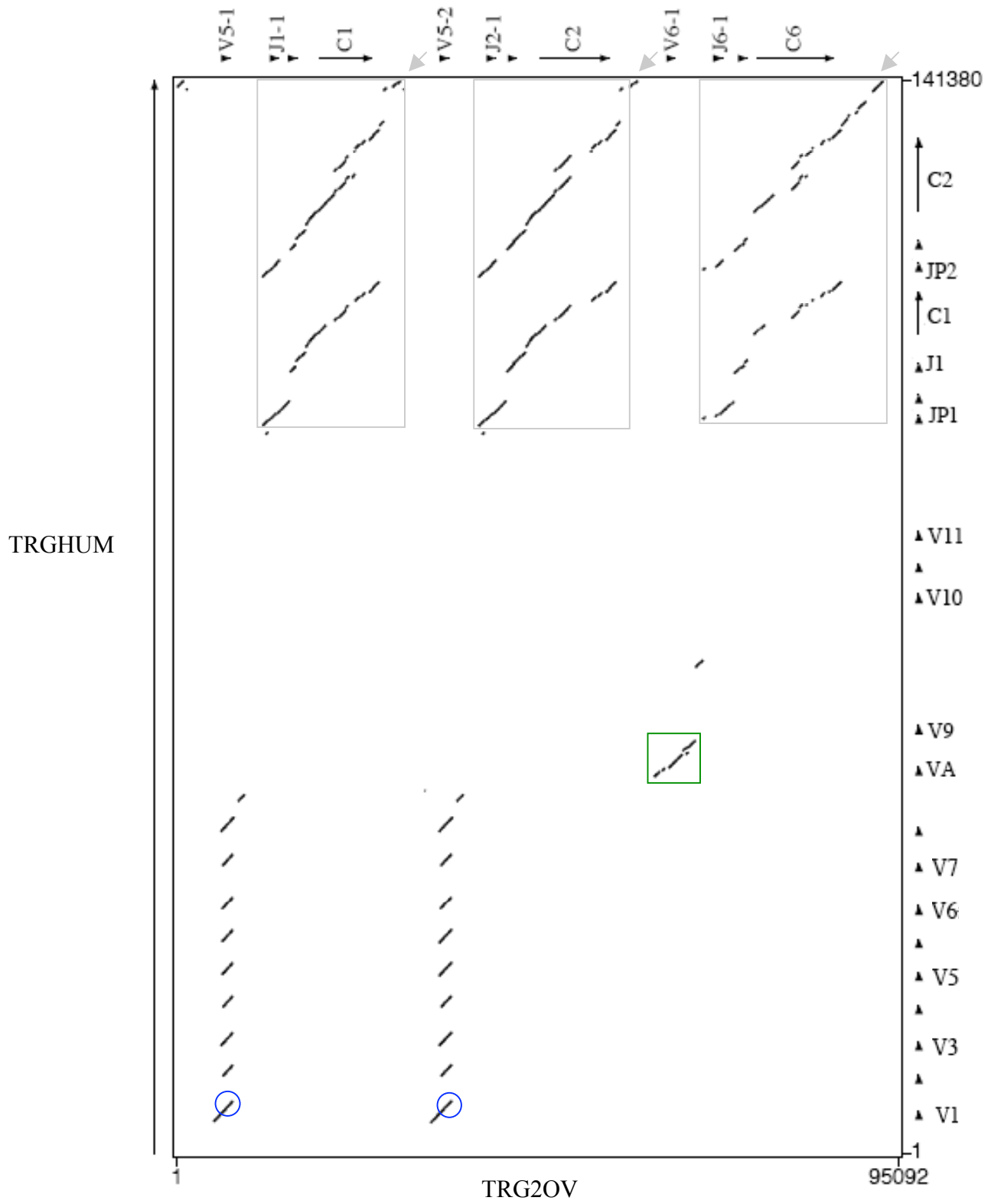

Supplement: Additional File 8 — Figure S8a, b – Genomic comparison of ovine and human TRG loci. The dotplots were obtained with the PipMaker program by using the complete sequences of ovine TRG1 and human TRG [GenBank: NG_001336] [GenBank: AC006033] (a) and ovine TRG2 and human TRG (b). The gene transcriptional orientation is indicated by arrows. The red rectangle includes similar regions between V genes belonging to ovine TRGC5 cassette and human V11, VB, V10 (a) while green rectangle highligths the similarity between V6-1 belonging to ovine TRGC6 cassette and human VA (b). Blue circles highlight similar regions between ovine V1, V2, V5, V8, V9 gene subgroups and human TRGV1 subgroup genes (a and b). The gray rectangles include conserved J-C containing regions, whereas gray arrows point to ovine enhancer-like sequences. [file 1471-2164-9-81-S8.pdf]

Genomic comparison of ovine and murine TRG loci

a)

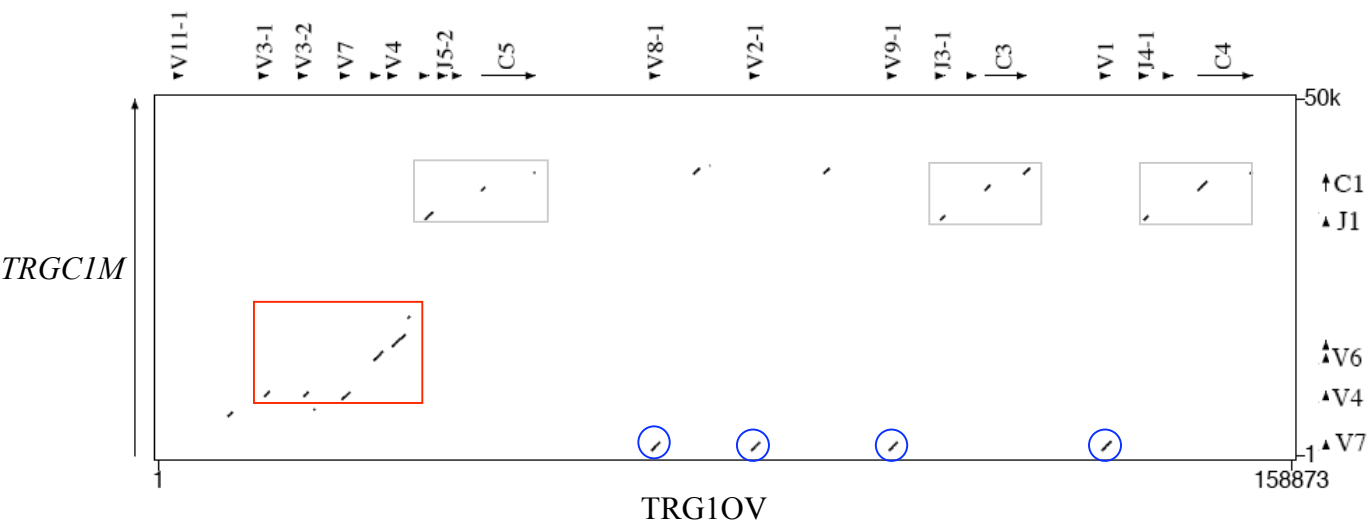

b)

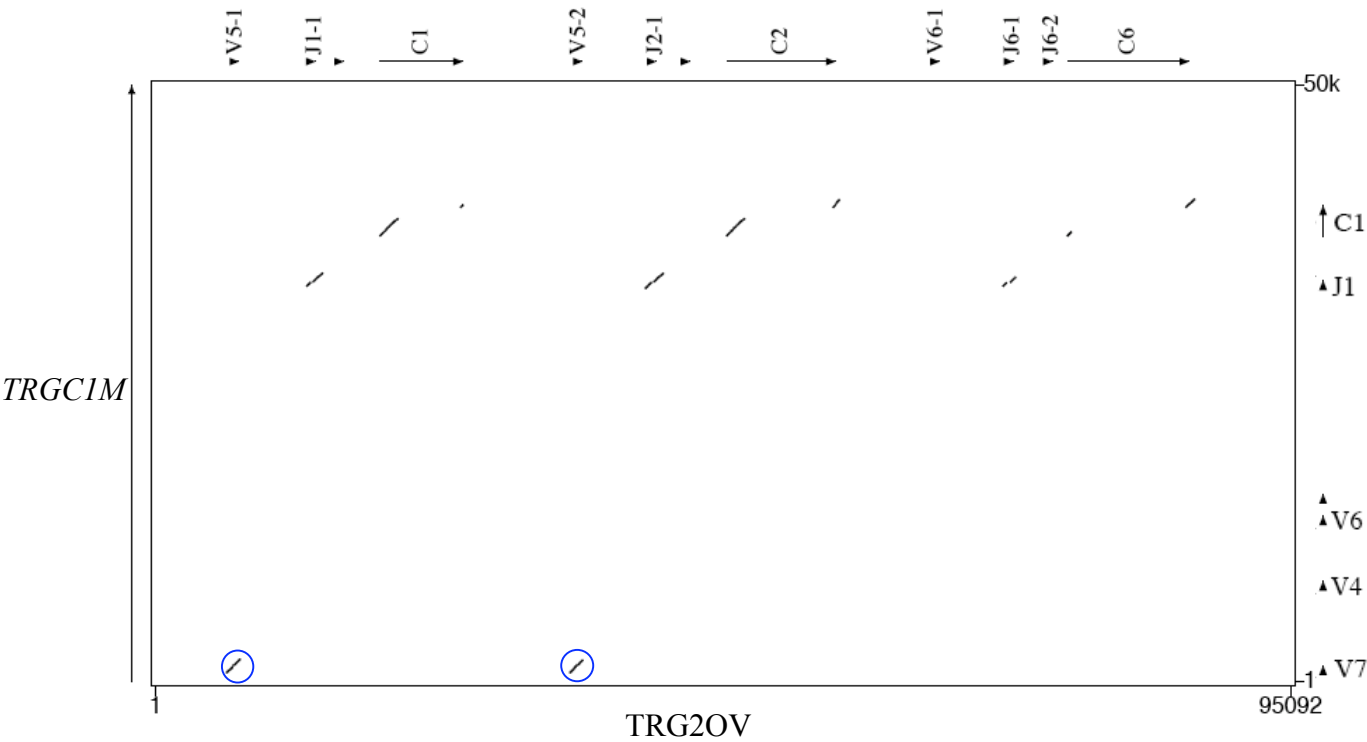

Supplement: Additional File 9 — Figure S9a, b – Genomic comparison of ovine and murine TRG loci. The dotplots were obtained with the PipMaker program by using the complete sequences of ovine TRG1 and murine TRG1 cassette [GenBank: AF037352] (a) and ovine TRG2 and murine TRGC1 cassette (b). The gene transcriptional orientation is indicated by arrows. The red rectangle includes similar regions between V genes belonging to ovine TRGC5 cassette and murine V4, V6, V5 (a). Blue circles highlight similar regions between ovine V1, V2, V5, V8, V9 gene subgroups and murine V7 (a and b). The gray rectangles include conserved J-C containing regions. [file 1471-2164-9-81-S9.pdf]
