# Supplementary material for: Genomic organization and recombinational unit duplication-driven evolution of ovine and bovine T cell receptor gamma loci
Source: BMC Genomics. 2008 Feb 18;9:81. doi: 10.1186/1471-2164-9-81 (PMC2270265; doi:10.1186/1471-2164-9-81)

Genomic comparison of ovine and bovine TRG2 loci

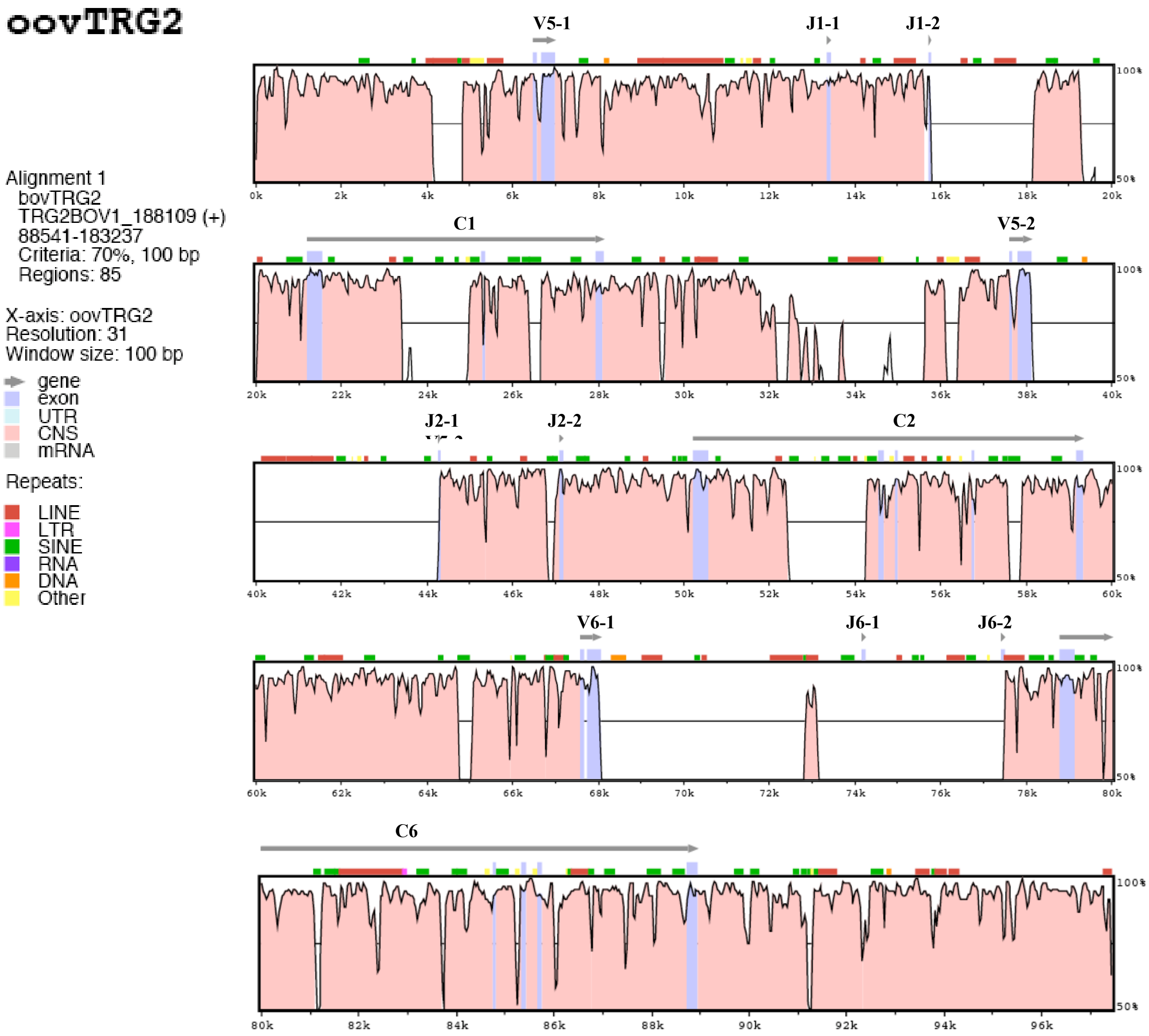

Supplement: Additional File 5 — Figure S5 – Genomic comparison of ovine and bovine TRG2 loci. The alignment of ovine TRG2 locus and bovine TRG2 locus [GenBank: AY644518] was obtained by the AVID program through the mVISTA server. The percent of conservation between the two hortologous sequences is reported on the vertical axis and is visualized as pink regions (conserved non-coding sequences or CNS) or dark blue regions (exon sequences). Genes are indicated by arrows; repeats are shown above the plot. [file 1471-2164-9-81-S5.pdf]
