# Supplementary material for: Genomic organization and recombinational unit duplication-driven evolution of ovine and bovine T cell receptor gamma loci
Source: BMC Genomics. 2008 Feb 18;9:81. doi: 10.1186/1471-2164-9-81 (PMC2270265; doi:10.1186/1471-2164-9-81)
Supplement: Additional File 6 — Table S6 – Similarity percentages of ovine TRGJ blocks pairwise alignments. Table presenting similarity percentages of ovine TRGJ blocks pairwise alignments (mVISTA). [file 1471-2164-9-81-S6.pdf]

Similarity percentages of ovine TRGJ blocks pairwise alignments.

	TRGJ5	TRGJ3	TRGJ6	TRGJ4	TRGJ2	TRGJ1
**TRGJ5**	-	2464bp - 73.3%	2937bp - 72.9%	3276bp - 72.8%	3199bp - 72.2%	1529bp - 74.1%
**TRGJ3**	2417bp - 73.2%	-	3483bp - 73.4%	3138bp - 72.1%	3887bp - 72.1%	2364bp - 73.1%
**TRGJ6**	2883bp - 72.4%	3543bp - 73.3%	-	5762bp - 75.2%	6216bp - 76.1%	2030bp - 75.4%
**TRGJ4**	3211bp - 72.4%	3439bp - 72.5%	5760bp - 75.3%	-	8406bp - 95.3%	2143bp - 73.4%
**TRGJ2**	3122bp - 71.8%	3878bp - 72.0%	6235bp - 76.1%	8406bp - 95.3%	-	2555bp - 72.5%
**TRGJ1**	1537bp - 74.1%	2373bp - 73.0%	2023bp - 75.2%	2253bp - 73.4%	2652bp - 72.5%	-
