# Supplementary material for: Genomic organization and recombinational unit duplication-driven evolution of ovine and bovine T cell receptor gamma loci
Source: BMC Genomics. 2008 Feb 18;9:81. doi: 10.1186/1471-2164-9-81 (PMC2270265; doi:10.1186/1471-2164-9-81)
Supplement: Additional File 7 — Figure S7 – Alignments of the retropositions in the six investigated TRGJ blocks. LINE L2, Sine MIRb, SINE BovA2, LINE L1M5, SINE BovtA2, CHR-L, SINE BovtA3, LINE L1 sequences are in upper case letters. The 5' and 3' ends of the retroposon insertions are boxed. For the detection of repeats, the Tandem Repeats Finder [36,37] and RepeatMasker [38] programs were used. The RepeatMasker analysis has been carried out using the Cetartiodactyla Repbase division section. [file 1471-2164-9-81-S7.pdf]

Alignments of the retropositions in the six investigated TRGJ blocks

```
R19:LINE L2

R19TRGJ5         catatggaatttgtgagttcagtgagccatctcagct-ccaaagttgatgaaaagtgtct 59
R19TRGJ6         gtaatggaatttgtgagctca---agccatctcagct-ccaaagtccatgaaaaaagtct 56
R19TRGJ3         agacttttactgcctcactta-ttagccatgtcttttaccaaagtccatgaaaactgttt 59
R19TRGJ1         atggaccagtttgtgagctcaatgagccatttcagt-tccaaagttcac-gaaagtgtct 58
R19TRGJ2         atggaccagtttgtgagctcaatgagccatttcagcctccaaagttcaccgaaagtgtct 60
R19TRGJ4         atgaacaagtttgtcagctcg---agtgatttcagct-ccaaagtctttgaaaatggtgt 56
                           *       *     **  ** **     *******      ***  ** *

R19TRGJ5         gcatggttgagcatccttatttgctt----ATTCTCTCAGCAAACATTTATTGAGGACAG 115
R19TRGJ6         gcatggttgaacatcctcacttgctc----ATTCTCTCAGCAAACATTTTTTGAGGACAG 112
R19TRGJ3         acatggttgaacatcctcatctgccc----ATTCTCTCAGCATTTATTTATTGAGGACAG 115
R19TRGJ1         tcatggctgggcattctcctttgctctTTCATTCTCTCAGCAAATATTTATTGAGGACAG 118
R19TRGJ2         tcatggctgggcattctcctttgctctTTCATTCTCTCAGCAAATATTTATTGAGGACCG 120
R19TRGJ4         gcctggttacatatcctcatttgctctTTCATTCTCTCAGCAAATATTTATTGA--ATTG 114
                  * *** *    ** **    ***      ************   **** ****  *  *

R19TRGJ5         TTTCTATGTTGGATGCTGGTGATAAAAG-GTGAAGAAGACACAAGACAGAACAAGTTTCA 174
R19TRGJ6         TTTCTATGT-GGATGCTGGAGACAGAAGTGTGAACAAGACACA-GACAGAACAAATCCCA 170
R19TRGJ3         TTTCTATGTTGGATGCTGGAGATAAAAGAGTAAGCTAGACAAA-GATAGAGTGAGTCCCA 174
R19TRGJ1         TTTCTGTGCTGGGAGCTGCAGACAGATG-ACGGGTGAACGAGA-CATGGAGCAAGTCTCA 176
R19TRGJ2         TTTCTGTGCTGGGAGCTGCAGACAGATG-ACGGGTGAGCAAGA-CTCAGAACAGGTCTCA 178
R19TRGJ4         TTTCTGTGCTGGAGGCTTGAGATAAAACTGTACACATGGCAGA---TAGAACAAGGAT-A 170
                 ***** **  **  ***   ** * *              * *     **         *

R19TRGJ5         ACACTCAAAGAG----TTTCTTGACTAGCAGAAAATCAt---ag-attccaacatgattg 226
R19TRGJ6         ACACTCAAACAG----CTTATAGTCTAGTGGAAAAGCAttctag-atgctggcttgagtg 225
R19TRGJ3         ACAGTCAAACAG----TTTCTATTCTACCCAATGAGCTttacag-attccggcttgattg 229
R19TRGJ1         ACAC-CAAACAG----CTTTTGTTCTAGTGGAAAGGCAttctag-attctggcttaattg 231
R19TRGJ2         ACAC-CAAGCAG----CTTCTGTTCTAGTGGAAAGGCAttctaacattccggcttaactg 234
R19TRGJ4         ACACTCAAACAAACCATTTCTATTCTAGTGGGATAGTGcttgaa-attctggtttaatgg 229
                 ***  ***  *      ** *   ***               *  ** *     **   *

R19TRGJ5         agcccaatgttttgtggcctgtttctgccagagaggcaagaagtcttgaactgatgagct 286
R19TRGJ6         ggctca-tattcaatggcttgtggctaccagagaaacaacagatcttggattgatgaact 284
R19TRGJ3         ggacccatgttctatgagctgtggctgctagacagacagcagatcttggattgatgggct 289
R19TRGJ1         agacctgtcttctatggtccatggctgc---agaactagggcgtcttgaattgttgacct 288
R19TRGJ2         agacctgtcttctatggttcatggctgc---agaatgagggcatcttggattgctgagct 291
R19TRGJ4         ggacccatgttctatgatctgtggctgc-agagaggcaggacaccttgatgaacttgttt 288
                  *  *  * **   **     *  ** *   * *   *      ****      *    *
```

R20:Sine MIRb

```
R20TRGJ5         taagcaatataaaatccatgtactttgttgga-agatttt---aatgaaatggccgc--- 53
R20TRGJ4         tattgtttataaaatctacaccttttgtttgatggaagac---agataaacggccttg-- 55
R20TRGJ1         tattgcttataaaaaacataccttttgtttgatggaagattttgatgaaatagcctc--- 57
R20TRGJ2         tattgctgataaaaatcatatcttttgtttgatggaagattttgatgaaatggcctc--- 57
R20TRGJ3         tatagcttataaaactcatatattttatctggtggaagaatttaatgaaatgtccttacc 60
R20TRGJ6         aaatggcaacccactccagtgttcttgcctggagaatcccagggatggcagagcctgg-- 58
                  *      *   *    *      **    *    *             *   **

R20TRGJ5         --AGACCATCTTTTtaaagagaataataatatcagCTACTATT---------TACTGAAG 102
R20TRGJ4         --AAACCATCCCATtaaagagaatttagtattagTCAATATTAGTTATATTTACTGAGG 113
R20TRGJ1         --AAACCAAGCCATtaaagagaatttaatattagTCAATATT---------TACTGAAG 106
R20TRGJ2         --AAACCATGCCATtaaagagaatttaatgttagTTAATATT---------TACTGAAG 106
R20TRGJ3         TTAAGTCATCCCACtaaagagaatttagtatcagCTTCCATT---------TACTGAGA 111
R20TRGJ6         --TGGGCTGCCATCtatggggtcgcacagagtcagACACGACTGA----AGCGACTTAG- 111
                       *       **  * *      *   * **     * *          *** *

R20TRGJ5         GCCAGACATCATGATAAGTAGTCTTAGGGCCTTATCTCTTTCCTCAACAGAAATTCTTAT 162
R20TRGJ4         GCCAGGCACCATGATAAGTA-TTTCAATGCCTTATCT-TTGCCTCAACAGGAATCCTTTC 171
R20TRGJ1         GATAGATACCATGATACGTAGTTTCAATGCCTTATAC-TTTCCTCACCAGTAATCCTTTC 165
R20TRGJ2         GATAGATACCATGACACATAGTTTCAATGTCTTATAC-TTTCCTCACCAGTAATCCTTTC 165
R20TRGJ3         ATCAGACAGCAAAGGAAG---TTTCAGTGCCGTATCTCATTCCTCAGCAGGAATCATCTT 168
R20TRGJ6         --CAGACATCATGATACGTAGTTTCAGTGCCTTATCT-TTTCCTCAAAAGGAATCCTTGT 168
                    **  * **    *     * * *  * * ***    * *****  ** ***  *

R20TRGJ5         ACTCCCATTTCATAGATAGGGAAACTGAGGTTGAGAGAAGTTCAGGCACTTGCCTAAATA 222
R20TRGJ4         ATTCTCATTTTATAGATAAAGAAACTGAGGTTTAGAGAAGTTCACTTAAATGCT---GTA 228
R20TRGJ1         ATTCCCATTTTAGAGATAAAGAAACTGAGGTTTAGGAAAGTTTAGTTACTTGCC---TAA 222
R20TRGJ2         ATTCCCATTTTAGAGATAAAGAAACTGAGGTTTAGAGAAGTTTAATTACTTGCC---TAA 222
R20TRGJ3         ATTCCCAGTTCATATATAAGGACACTGAGGTTTTAAGAAGCTCAGTTACTAGCC---TAA 225
R20TRGJ6         CTTCCCATTCCACAGATAAGATAACTGAGGTTTAGAGAAGTTCAGGTGCTTGCC---TAA 225
                   ** ** *  * * ***     *********     *** * *       **      *

R20TRGJ5         AAGTACATGACTAGTTACTG-TGGTGAAGTTTGGAATCTGATAGTCTATTCCTAAG--TG 279
R20TRGJ4         AGTCACATGACTAGTTACTGCTGGCAAAGTTTGGGATCCAATAGGA-ATTCCCTTG--TG 285
R20TRGJ1         AGTCACATGACTAGC-ACTG-TGGCAAAGTTTGGGATCCAGTAGAGACTCGTCAAAC-TT 279
R20TRGJ2         AGTCACATGACTAGCTACTG-TGGCAAAGTTTGGGATCCAGTAGAGACTTGTCAAAC-TT 279
R20TRGJ3         AGTCACCTGACAAGATACATTGCAAAAAGTTTAGGATTCAATAAGGATTTGTCTATTCCA 285
R20TRGJ6         AGTCACATAACAAGTTACTG-TGGCAAATATTGTAATTCAGTAGAGACTTGTTTATT-CC 283
                 *   ** * ** **  **        **   **   **    **     *

R20TRGJ5         CATGGTCTGG------ATcattaagtgatgtgagaagcatcctctttttatattctgaat 330
R20TRGJ4         GCTCAGCTGGTAAAGAATctgtttgtaatgtgagagac---ctggggtgaa-tccctggg 341
R20TRGJ1         AGAGGACTGG------ATcattacgtgatatttgaaaca--ttaagtcat-gtctttaca 330
R20TRGJ2         AGAGGACTGG------ATcattacgtgatatttgaaaca--ttaagtcat-gtctttaca 330
R20TRGJ3         AAGTGAATGGTCT--GGActgttattgatgttagaaacat-ttaagttat-gtttttata 341
R20TRGJ6         AAAGGACAGAGTCTGGACcattacataaggtgagaaaaa--tgaagtctaagtttttata 341
                         *         *  *   * *  *  **                 *
```

R31:Sine MIRb

```
R31TRGJ3         tatgttcatgaaagctggaagccttgatatggggc-------------agttaaaagtca 47
R31TRGJ6         tgtgttcatgaattttggaagg--------------------------agataaaactca 34
R31TRGJ4         tatattcatgaattttggaagccttgatatggagtattcacatggcgtatttaaaagtca 60
R31TRGJ5         tttgtttatgaatgtcacaagcattgatatggggc-------------atttttaagtat 47
R31TRGJ1         --gagtcagacacgactgaagc---gacttggcat-------------gcacacaagatg 42
R31TRGJ2         --gagtcggacacgactgaagc---aacttggcat-------------gcacgcaagatg 42
                      *     *      ***                                 **

R31TRGJ3         taccccatcataacatgtggagaaagaacaaatgtcaaGGT-TAATCCAGCTTCTGTTAC 106
R31TRGJ6         tacctcatcttaccatatggaaaaagaacaaaggccaaCGTGTGATCCAGGTTCTACTAC 94
R31TRGJ4         tacgccatctttagaaatggagaaa-aatgaaggtcaaGGCATAATCTAGCTT-TACTAC 118
R31TRGJ5         cagtccaacttagcacttggaaaggaaaccaaagccaaGACGTGGGCGAGCCTCTGCTTC 107
R31TRGJ1         caccccatctctgtatatggagagaaacggaaggtcaaCTTATAATCCAGCTC-TGCTAC 101
R31TRGJ2         caccccatctctgcatatggagagaaacggaaggtcaaGTTATAATCCAGCTC-TGCTAC 101
                  *   ** *     *  **** *   *   ** * ***    *   * **    *  * *

R31TRGJ3         ATATTAGCTGGGACTTT----AAGTTTTATGATGTCTTGAACCTCTCTTCCCATTGAAAA 160
R31TRGJ6         GC--TAGCTGTGACTTTGGTCAAGTTACATAACCTCTTGAACGTCTCTTCCTATTGAAAA 152
R31TRGJ4         TTAAGAGCTCTGACTTTGGGCAAGTTACATGAAGTCTTGAACTTTCCCTTCCATCAAAGA 178
R31TRGJ5         TGAAAAACTGTGACTTTGGGCAAGTGATATAACTTTTTGAACATCTCTTCCCATTGAA-A 166
R31TRGJ1         TTAACAGCGGTGACTTTGGGCAAGTTCCATGAAGTCTTGAACTTCTCTTGCCATTAAACA 161
R31TRGJ2         TTAACAGCGGTGACTTTGGGCAAGTTCCATGAAGTCTTGAACTTCTCTTGCCATTAAAAA 161
                      * *   ******    ****   ** *  * ****** *  * * * **  ** *

R31TRGJ3         TAAGAACAATAAAACACCCTTCACCaccttctgtggatgaaca-ac----caagctataa 215
R31TRGJ6         TAAGGATAGTAAG---TCCCTCACAatctactgtgaatggaaa-ccatggcgagcactag 208
R31TRGJ4         TAAGAACAATAATCAGCATCTGACAatctattgtggatgaaaggccacggtgagcactgg 238
R31TRGJ5         TAA-AACAATAATAGGTACCTCATCatcttctgtggatgaaaagccatgatgagagttag 225
R31TRGJ1         TAA-AATAGTACTAAGCACCTAACCacgtactgcggatgaaaggccatagtgagcagtag 220
R31TRGJ2         TAA-AACAGTACTAAGCACCTAACCatatactgcggatgaaaggccatagtgagcagtag 220
                 ***  * * **         * *  *  *  ** * *** *    *      **   *

R31TRGJ3         ttcaatgacaaattttcctcctcttaccacttgctaggttgttccaaatcacactaacat 275
R31TRGJ6         ttcagtgacatgttttcttcctttta ttacacgctagtttattccatggttctccgtgtt 268
R31TRGJ4         ttcaatgatg-----cctttcttttaccactcgctgtgctgtcctcagtcattc-actcg 292
R31TRGJ5         ttcactggtagcttttcttcttttcactctaagctaagttgttccaaacttcatgacatg 285
R31TRGJ1         ccttttg-----------ttttttcactactcactaagtttttccaagccacaacatgtt 269
R31TRGJ2         ccttttg-----------ttttttcactactcactaggtttttccaagccacaacatgtt 269
                      **           *  * * *       **    * * *
```

R23: Sine BovA2

```
R23TRGJ5        gtgcaaacaggtggccagagcatcgaagtgtttggttccag-aacacaacttatt--gtt 57
R23TRGJ6        ccatttgttttggacaacaaaagtgaagtaattccatatactgactatacatattaggct 60
                            * * * *  *  *****  **   *  *   **   ** ****  * *

R23TRGJ5        acaggcgaagtttcttta--AATTCTGTAGTGAGGTAGGGATAGGAG-----GTAGGCAA 110
R23TRGJ6        ttagacaatgttcccttaTTGCTTCCCCGGTGACTCAGAGGTTAAAGCGTCTGCCCGCAA 120
                  ** * * *** * ***    ***    ****   ** * *   **     *   ****

R23TRGJ5        TACAGGCACTGCAGGCTTGAGTGTTGGTGCTGGC---TCAGATGGAGAAGGCAATGGCAA 167
R23TRGJ6        TGCAGGAGACCTGGTTTCAATCCCTGGGTCAGGAAGATCCCCTGGAGAAGGAAATGGCAA 180
                * ****       *  *  *    ***  * **    **   ********* ********

R23TRGJ5        CCCACTCCAGTACTCTTGCCTGGAAAATCCCATGGATGGAGAAGACTGGTAGGCTGCAGT 227
R23TRGJ6        CCCACTCTAGTATTCTTGCCTGGAGAATGCCATGGACGGAGGAGCCTGGTGGGCTATAGT 240
                ******* **** *********** *** ******* **** ** ***** ****  ***

R23TRGJ5        ACATGGGGTCATGAACAGTCGGACACGACTGAGTGACTTTACTT--TCACTTTTCACTTT 285
R23TRGJ6        CCACGGGGTGGCAAAGAGTCAGATACGACTGAGTGACTTCACTTGTTCAATGTTCCCTTT 300
                 ** *****    ** **** ** *************** ****  *** * *** ****

R23TRGJ5        C----CTGCATTGGAGAAggaaatggcaacccactccagtgttcttgcctggagaatccc 341
R23TRGJ6        CGAGGTTGCTTCACCCGTggaaa-gacagt--ggctaagagctacagacccaagtgcctt 357
                *     *** *       ***** * **         ** * *   * *   **   *

R23TRGJ5        agggatgacggagcctggtaggc-tgccatctgtggggtcgcacagagtcagacacaact 400
R23TRGJ6        taaggtg--tgtgtatgaaaagcacgtgccctatgaca----atggcgttaagaattcct 414
                   * **   * *  **  * **  *    ** **       *  * ** *   *   **
```

R24: AT rich

```
R24TRGJ5        tggctaaatgtaatgagtgtaatttca-aatgttagaatataaggt------gctctctc 53
R24TRGJ6        tcagtaagggcaa--gccctggtcccatgatattagaatattatgttggagcacacaatc 58
                   ***  * **      *  *  **  ** ********* * **       * *  **

R24TRGJ5        atcttgtagta-gaaatGTTTGGAAATTAAAAATATTGTATTT-TTAAAATAtgagtttt 111
R24TRGJ6        agctagtaataagaaatCACTAATAATAATGATAATAATAATTATTATGGTCttcttttt 118
                * ** *** ** *****   *   *** *  *  **  ** ** ***   * *   ****

R24TRGJ5        t----gcaacttgaacatacaagcttatagggatacatacaaaagtcaaat--attcaag 165
R24TRGJ6        ccatagcattttaatacaatcacttttcaaatcttgtttcatgttggtctcacttcag 178
                     ***  ** ** * *    *** *    **        * **       * *  **
```

R15: Sine Mirb

```
R15TRGJ5        aattat--tgattttgtatgcagc-gctaagatttttctgttctttgcgagaaagtgaa 57
R15TRGJ3        cattaccctgtgcttcctctgtggctgctgggaacacttggcaacggctgagcaccacta 60
                 ****   **   **  * **  ** ***  **    *  * *** *     *

R15TRGJ5        ttctttgttcctgaattttgcctcatatcttTCTAGACACATTATCTCATGTGAACTTTAC 117
R15TRGJ3        atttccagacctgaacttgacctcattttttCTGACTTAGGTACTATCAT----ATCCTCC 116
                 * *     ****** **  ****** * *   *   *  *  * ****    *   * *

R15TRGJ5        AAAAGTACTGAGGAATAGATAACAACACCTCCCTTTTAAAGATGAGGAAACTGACACTCT 177
R15TRGJ3        CTTACCCCTAATGCATGGTGAA-----CTTGGGATTCAGAGA--AACTCTGTAGCA-TCC 168
                   *   ** * * ** *  **     * *    ** * ***  *      *  ** **

R15TRGJ5        GAGATGTACCCAAGAACATGTCACCAGGAGTGGCGGGGTTAAAATTGGAGCTCTATCCTT 237
R15TRGJ3        AAGAT-CACAAAGGTAT-TGATATCAGAAGAGACACAGTTTGAAGCCAAG---TATAAAT 223
                 ****  **  * * *  **  * *** ** * *   ***  **    **   ***   *

R15TRGJ5        CTGATTTTAAAACATCTTTTCCTTCactttcaacctcctgagcgttaatttgatttctcc 297
R15TRGJ3        GTCATTTAAACATATGCTTTTCCCTacactctaccc----agaaccaacaagaagt---- 275
                 * **** ** * **  *** *   **  ** ***     **    **   **   *

R15TRGJ5        catttgattttcttagaaacctgatgaacgtgttaatatttactgcacca- 350
R15TRGJ3        -aatgagaggataaagaagagaagtggggagtcagggtgatctgtatatt 325
                 * *          ****      **   * ** *   *   *** *
```

R25: Sine MIRb

```
R25TRGJ5         tggcccaaggccaaagtttattactacaggtaagttttgtgagttgacacatttcctttt 60
R25TRGJ3         tggctcaaggtttacatttgttattacaagtaagttttgtgagttgacacgtttcctttc 60
R25TRGJ4         --gcccaaggttgaagtttgtcattacagctaagttttgtgagttgacaggtttcctttt 58
R25TRGJ1         --gcccagggttgaagtttgtcattacaggtaagctttatgagttaacatgtctcccttc 58
R25TRGJ2         -ggcccagggttgaagtttgtcattacag-taagttttatgagttaacatgtctcccttt 58
                              *  *** * * ****  **** *** ****** ***  * *** **

R25TRGJ5         ctgatgatgaaaggatgtttttAAGGAATGTAAGCTTTGGG-------TAAGGTGGAGCT 117
R25TRGJ3         ctgatg-tggaagggtgtttc-AAAGAACCTGGGCTTTGGAATCAGGTTGACTTGGAACT 118
R25TRGJ4         ctgatgctgaagatgtattttcAAA-AACATGGGCTTTCGAGTCAGGCTGACCTGGAGCT 117
R25TRGJ1         ctgatgctgaaggggtatttccAAAGAAAGTGGGCTTTGGAGTCAGGCTGACCGAGAACT 118
R25TRGJ2         ctgatgctgaaggggtatttccAAAGAAAGTGGGCTTTGGAGTCAGGCTGACCGAGAACT 118
                 ****** ** *    * ***  **  **  *  ***** *        * *    ** **

R25TRGJ5         CTAGATCCATCACTTACTGCTTATATGAACTTGGA-GATGATACTCTGGCTTCAGTTTCC 176
R25TRGJ3         TCAGGTGTACCACTTACTGCTTATGTGAATTTGGGTAATTATTTTCTGGCTTCAGTCTCC 178
R25TRGJ4         TCCGACCCACCACTAACTACTTATGTGAACATGGG-GGTGATTCTCTGGCCTCAGTCTCC 176
R25TRGJ1         TC-GATCCACCACTAACTACACATGTGAACATGGA-AGTTCTTCTCTGGACTCAGTCCCC 176
R25TRGJ2         TC-GATCCACCACTAACTACACATGTGAACATGGA-AGTTCTTCTCTGGACTCAGTCTCT 176
                    *    * **** *** *  ** ****  ***    *  *  *****  *****  *

R25TRGJ5         TAATCCGTAAA-CAAGATAACAATATGTACCCAGCAGGGGTTTTTGGAA-TTGAATGAG 234
R25TRGJ3         TAATCCATAAAGTGGGATAATAATGTATACCCAGTAGGGGTTGTATGAAAATTGAATGAG 238
R25TRGJ4         TAATCCATAAGATAGGATAACAGTGTATATTCAGCAGGGGTAGTAGGAGAATTGAATGAG 236
R25TRGJ1         TAATCTGTAAAATAGCATAACAGTAT-TATCCAGCAGGGGTTGTACGAGAACTAAATGAG 235
R25TRGJ2         TAATCTGTAAAATAGCATAACAGTAT-TATCCAGCAGGGGTTGTACGAGAATTAAATGAG 235
                 *****  ***      **** * * * **  *** ******  *  *  *  * ******

R25TRGJ5         ATAGTGTGAA--TATAGAACTTTGCACATTGACTTA--CTTATCATGGGTGTTGGATA-A 289
R25TRGJ3         ATAATGTGTAGCTATCATATTTGGTACATTGTCTTGGGCAGATAATAGGGAATGTATATA 298
R25TRGJ4         ATAATGTGAA--TAAAGTACTTAGTACACTGGCTAA--CACCTCATAGGTGCTGGATAGG 292
R25TRGJ1         ACAGTGTGA---TGAAGTATTTAGCACATTGTCTAA--TGCATCAGTATTCCTGTCTGGA 290
R25TRGJ2         ACAGTGTGA---TGAAGTATCTAGCACATTGTCTAA--TGCATCAGTATTCCCGTCTGGA 290
                 * * ****    *     *  * * *** ** **        * *        *  *

R25TRGJ5         TGtaactacaacctattcaaa--------gagaattatatccaggatggagctatttgca 341
R25TRGJ3         TGtacctacactcaattcaaatgttaagggagaattgtatgcagtagggagttgtttgca 358
R25TRGJ4         TGt---------ttattcaaatgtaaagtgaaaatggcatccagaagggagagtttttg 343
R25TRGJ1         TAagcc-----cgtggacagaggagccaggcgggctacagtc--catggggttgcaaaga 343
R25TRGJ2         TAagcc-----cgtgcacagaggagccaggcaggccacagtc--catggggttgcaaaga 343
                 *                **  *        *        *  *   * ** *
```

R17: LINE L1M5

```
R17TRGJ1             ----tatatcaatttaaaacaaatataactagctcagccccaatctttataacagggaac 56
R17TRGJ2             -ttttatatcaatttaaaacaaatataactagctcagcctcaatctttacaacagggaac 59
R17TRGJ3             attcttaatcatatttaatgttatttaacttgct-aaccctgaaccttataccatagaac 59
R17TRGJ4             ------tttcaaaattcttacaacataattagctcaaccccaatctttacaatatacaac 54
                             ***   *       *  *** * *** ***    * ** ** *  *   ***

R17TRGJ1             aaaaaaataaaatgttaccttaAGTCTCATTGGAATTAGTCTA--TTTATACACATGATT 114
R17TRGJ2             aaaaaaataaaatgttaccttaAGTCTCATTGGAATTAGTCTA--TTTATACACATGATT 107
R17TRGJ3             aaaaaa-tgaaatattttcttaATTCTCATAAGAATTATAGTAACTTCATAAATGTGAAT 118
R17TRGJ4             aacaacaaataaagtttacttaACTCTGATTGGAATTAGAGTC----AATTTATGTGATT 110
                     ** **     **  **  ***** *** **  ******   *      **  *  *** *

R17TRGJ1             TTAAGAAGAGTTTGCATGTTTATGACATTGAGCTTTTCCTTCATGAATATGATATGCCTC 174
R17TRGJ2             TTAAGAAGAGTTTGCATGTTTATGACATTGAGCTTTTCCTTCATGAATATGATATGTCTC 167
R17TRGJ3             TTAGGAAAGATCAGTATCTTTATGATATTTAGTTTTTCTTACACAAATGTGATATGTCTC 178
R17TRGJ4             TTAGGAAGGACTAGCATGTTTATGATACGGAGTTTTTCTTTCATGAATATGATATGTCCC 170
                     *** ***      * ** ******* *   ** ***** * **  *** ******* * *

R17TRGJ1             TCCATTTATGCAGGTTTAATTTTATGCCCTTTAGTAAAATCTTTTACTCTTTCTTCTTAC 234
R17TRGJ2             TCCATTTATGCAGGTTTAATTTTATGCCCTTTAGTAAAACCTTTTACTCTTTCTTCTTAC 227
R17TRGJ3             TTTATTTATTCAAGTTTAATTTTATGCCCTTTA-TAAAAACTTGTATTCTTTCTTCTTAG 237
R17TRGJ4             TCTAAGTGTGCAGGGTTTTTTTCA-ACCCTTTAGTAAAATCCTGTACTGTTTTCTTCATA 229
                     *  *  * * ** * **    *** *  ******* *****  * * ** * ***    *

R17TRGJ1             TGGTCTTATATt-aataaagcattaattacata-ctcttta-ttctttgcattctatgct 291
R17TRGJ2             TGTTCTTATATtttaataaagcattaattacataactctttcgttaatctttcgcatttta 287
R17TRGJ3             AGCTCTGGAAttttcttataaatttgacaaatgattttataactctttacttactctttt 297
R17TRGJ4             TGGTCTTGTAttattttgtaaatataatcaaagattttgtaactgtttacctattct-tt 288
                      * ***   ***   *     **        *    * * *   *  *         *
```

R21: Sine BovtA2

```
R21TRGJ3             caa--aaagtttaggattcaataaggatttgtctattccaaagtgaatggtctggactgt 58
R21TRGJ4             ctggcaaagtttgggatccaataggaattcccttgtggctcagctggtaaag-aatctgt 59
                     *    ******* **** ***** * ***    * *  *  **    *        ****

R21TRGJ3             tattgatgttagaaacatttaagttatgtttttatatcctgagtaTTTACAGTTCATAGC 118
R21TRGJ4             ttgtaatgtgagagac--ctggggtgaatccctgggttgggaagaTCCCTGGAGAAGGGA 117
                     *  * **** *** **    *  * *   *   *   *   **  **      *   *   *

R21TRGJ3             CTG---ACCTTTTCCAGTATCCTCACCTGAAAAATCCATGGTCAGAGGAGCCTGGCAGGC 175
R21TRGJ4             AAGGCTACCCACTTGAGTATTCTTGCCTAGAGAATTC-------------CATGGATTGC 164
                       *   ***   *  ***** **  ***  * *** *             * ***     **

R21TRGJ3             TACAGTCCAAAGGGTCTCAAAGAGTCAGACACAACTGAGTGACTAA-GCACtcagcctca 234
R21TRGJ4             -ATACTCCATAGGGTCACAAAGAGTCGGACATGACTGAATGACTTTCACACtcacacaga 223
                      * * **** ****** ********* ****  ***** *****    ******  *  *

R21TRGJ3             ccatt-taaatgagtactttgattgactacttagtagcttataattagctttactctcac 293
R21TRGJ4             gacttgtttattctgaagtaagtggactgggccattacatgatattagaaacattaagtc 283
                        ** *  **     *  *    * ****         *  * *   *****    * *    *
```

R27: CHR-L

```
R27TRGJ1         ---aggcagaagttcccaagagttttcttttcttctcagatgagaagtccaatttctccc 57
R27TRGJ2         ---aggcagaagttcccaagagttttcttttcttctcagatgagaagtccaatttctccc 57
R27TRGJ4         cataggccaaaattctgaataat--tcttttcttctcatgtgagaagtccaatttttctt 58
                    ****  ** ***  ** * *  *************  *************** **

R27TRGJ1         ttcttaagagatttccccgtgaAGGACTTTCCCAGTGGTCCAGCGGTTAAGACTCCACCT 117
R27TRGJ2         ttcttaagagatttccccgtgaAGGACTTTCCCAGTGGTCCAGCGGTTAAGACTCCACCT 117
R27TRGJ4         ttcttaagatatttcccagtgaGGGGCTTCCCTGGTGGTTCAATGGTTAAGATTCCTCCT 118
                 ********* ******* **** ** *** **  ***** **  ******** *** ***

R27TRGJ1         TCCAATGCAGGTG-CACAGGTTCGAACCCTTGTCAGGATACATGCTTTATGCAGCTGGTG 176
R27TRGJ2         TCCAATGCAGGTG-CACAGGTTCGAACCCTTGTCAGGATACATGCTTTATGCAGCTGGTG 176
R27TRGJ4         TCTAGTGCAGGGGGCACAGGTTCAATTCCTGGTTGGGATATACACTCTATTCAGTAGGTG 178
                 ** * ****** * ********* *  *** **  ***** *  ** *** ***  ****

R27TRGJ1         GAAAAAAAGAAAAtagtggcagt--aaagcagttcagcagccttagggggcaaaattcaa 234
R27TRGJ2         AAAAAAAAGAAAAtagtggcagt--aaagcagtacagcagccttagggggcaaa-ttcaa 233
R27TRGJ4         GAAAAGA-GCAAAtagtggtgctccaaagcacttcagcagccttatagggtagg-ttcaa 236
                  **** * * *********   *  ****** * ***********  *** *   *****
```

R16: AT rich

```
R16TRGJ1         --------atgtcacagtattcttactccattattgtgttatgtcagtaactttgcaata 52
R16TRGJ2         ttttattcatgtcacagtattcttacaccattattgtgttatgtcagtaactttgcgata 60
                         ****************** ***************************** ***

R16TRGJ1         agggttaagagctgctatgccaaattagTTATTTAAAATCATTATTTTAATAAAAAAATT 112
R16TRGJ2         agggttaagagctgctatgctaaattagTTATTTAAAATCATTATTTTAATAAAAAAATT 120
                 ******************** ***************************************

R16TRGJ1         T-AAGTTATTTCATATTTCATTTTTATATCAATTTAAAACAAATATAACtagctcagccc 171
R16TRGJ2         TTAAGTTATTTCATATTTCATTTTTATATCAATTTAAAACAAATATAACtagctcagcct 180
                 * *********************************************************

R16TRGJ1         caatctttataacagggaacaaaaaaataaaatgttaccttaagtctcattggaattagt 231
R16TRGJ2         caatctttacaacagggaacaaaaaaataaaatgttaccttaagtctcattggaattagt 240
                 ********* **************************************************
```

R18: Sine BovtA3

```
R18TRGJ1         atcaatgttttcagtgattactacttcttggaaagcagatatttgggtccatttatctga 60
R18TRGJ2         atcaatgttttcagtgattactacttcttggaaagcacatatttgggtccatttatctga 60
                 ************************************* **********************

R18TRGJ1         ggaaagtggcttccccagtgGATCTGTGAGTCAGGAAGATTCCCTGGAGA-GGAAATGGC 119
R18TRGJ2         ggaaagtggcttccccagtgGACCCCTGAGTCAGGAAGATTCCCTGGAGAAGGAAATGGC 120
                 ********************** *  ************************ *********

R18TRGJ1         AACCGACTCCAGTATTCTTCCCTGGGAAATCCCATGGAAGGCTACAGTCCAAGCGGTTGC 179
R18TRGJ2         AACCCACTCCAGTATTCTTCCC-GGGAAATCCCATGGCAGGCTACATTCCAAGGAGTTGC 179
                 **** ***************** ************** ******** ******  *****

R18TRGJ1         AAAGGAGTTGGACACATCCAAGTGACTgaataataacaataaatctgagggaaatcttat 239
R18TRGJ2         AAAGGAGTTGGGCACATCCAAGTGACTgaataataacaataaatctgagggaaatcttat 239
                 *********** ************************************************

R18TRGJ1         aaaacattggctgctgtgaaacctatatccaaggacttccaaaagaa 286
R18TRGJ2         aaaacattggctgctgtgaaacctatatccaagaacttccaaaagaa 286
                 ********************************* *************
```

R26: Sine BovtA2

```
R26TRGJ1            taacagtattatccagcaggggttgtacgagaactaaatgagacagtgtgatgaagtatt 60
R26TRGJ2            taacagtattatccagcaggggttgtacgagaattaaatgagacagtgtgatgaagtatc 60
                    ********************************* *************************

R26TRGJ1            tagcacattgtctaatgcatCAGTATTCCTGTCTGGATAAGCCCGTGGACAGAGGAGCCA 120
R26TRGJ2            tagcacattgtctaatgcatCAGTATTCCCGTCTGGATAAGCCCGTGCACAGAGGAGCCA 120
                    ***************************** ***************** ************

R26TRGJ1            GGCGGGCTACAGTCCATGGGGTTGCAAAGAGTTGGACACGACTGAGCactgtatgttttg 180
R26TRGJ2            GGCAGGCCACAGTCCATGGGGTTGCAAAGAGTTGGACACGACTGAGCaccgtacgttttg 180
                    *** *** ***************************************** *** ******

R26TRGJ1            ttggctaacacatcatatgtgttggataggtgtttactctattcaaatgtaaagggaaaa 240
R26TRGJ2            ttggctaacacatcatacgtgttggataggtgtttactctattcaaatgtaaagggaaaa 240
                    ***************** ******************************************
```

R29: CHR-L

```
R29TRGJ1            -tattcccaccacctgcccatccacttcaaaacaggctccagtattatagaacaaaagag 59
R29TRGJ2            ctattcccaccacctgcccatccacttcaaaacaggctcca-tattatagaacaaaagag 59
                     **************************************** ******************

R29TRGJ1            atttttaaaaagattaattggGGACTTCCCTGGCAGTCCAGCGATTGGGACTCCACATTC 119
R29TRGJ2            atttttaaaaagattaattggGGACTTCCCTGGCAGTCCAGCGATTGGGACTCCACATTT 119
                    ***********************************************************

R29TRGJ1            CCACTCTAGGAAGCATGGATTCAGTCAAT----------------tctggaagtcttgat 163
R29TRGJ2            CCACTGTAGGAAGCATGGATTCAATCAATAAACATGCTCATGAATtctggaagtcttgat 179
                    ***** ***************** *****                ***************

R29TRGJ1            acggggtatttatatgggatatttaaaagtcataatcccaatggacagaggagactggca 223
R29TRGJ2            gtagggtatttatatgggatatttaaaagtcataatcccaatggac-------------- 225
                       *******************************************
```

R28: Line L1

```
R28TRGJ1            ---------tttttgtgtgtgtgtattatttttcagattcttttccatta----aAGGTT 47
R28TRGJ2            gtgattcagtttttgtgtgtgtgtattatttttcagattcttttccatta----aAGGTT 56
R28TRGJ4            ---------tatctgtgtgtgtatagtctttttcaggttctttcccattattacaAGGTT 51
                             * * ********* ** * ******** ****** ******    ******

R28TRGJ1            ATTACAAGATATTTAACATACTTCTCTGTTCTACATAGTAGGTTCTTATTG-TTTATCTA 106
R28TRGJ2            ATTACAAGATATTTAATATACTTCTCTGTTCTACATAGTAGGTTATTATTG-TTTATCTA 115
R28TRGJ4            ATTACAAGATATTGGATATAGTTCCCTGCGCTATATAGTAGGTCCTTATTGGTTTATCTA 111
                    *************  * *** *** ***  *** *********  ****** ********

R28TRGJ1            TTTTATATGCAGTAGTGTG-TATCTGCTAATCCCAaattaggagtttgggattaataata 165
R27TRGJ2            TTTTATATACAGTAGGGTG-TATCTGCTAATCCCAaattaggagtttgggattaataata 174
R28TRGJ4            TTTTATATATAGTATTGTGCTATCTGTTAATTCCAaattaggagtttgggattaattata 171
                    ********  ****  *** ****** **** ************************ ***

R28TRGJ1            cattttcaatagaagttttggagatactgttat---------acaatcaaggcactataa 216
R28TRGJ2            cattttcaatagaagttttggaaatactgttat---------acaatcaaggcactataa 225
R28TRGJ4            cattttaaatagacattttagaaatatagctatcttgcttccacattcaaggcactgtaa 231
                    ****** ******  **** ** ***  * ***         *** ********** ***

R28TRGJ1            ataacatgctgctctactccaaaatctggagtggttcaggtataaaattgagaggtcata 276
R28TRGJ2            ataacatgctgctctactccaaaatctggagaggttcaggtataaaattgagaggtcata 285
R28TRGJ4            ataatgtactgctctactaaaaaatctggagtggttcagatgtaaaattgagaggttata 291
                    ****  * **********  *********** ******* * ************** ***
```
