# Supplementary material for: Genomic organization and recombinational unit duplication-driven evolution of ovine and bovine T cell receptor gamma loci
Source: BMC Genomics. 2008 Feb 18;9:81. doi: 10.1186/1471-2164-9-81 (PMC2270265; doi:10.1186/1471-2164-9-81)
Supplement: Additional File 10 — Table S10a, b – Plasmid subclones covering the entire sheep TRG1 and TRG2 loci. Tables presenting list of the plasmid subclones covering the entire sheep TRG1 (a) and TRG2 (b) loci. [file 1471-2164-9-81-S10.pdf]

Plasmid subclones covering the entire TRG1 and TRG2 loci.

a)

Clone	Approximate size (bp)	Cloning sites	Gene content	Gene content (IMGT name)	Source[a]
PCR	13390	Not I/EcoR V	V9P	V11-1	BAC 202C5
pBSVG3	2569	EcoR V	V3.1	V3-1	"
pBS22	2860	EcoR V			"
pBS8E	892	EcoR V			"
pBSVG3B	4103	EcoR V	V3.2	V3-2	"
PCR	421	EcoR V/Hind III			"
pBSVG410.0	9458	Xho I/Not I	V7-VP-V4	V7/V10-1/V4	BAC 655D3
pBSVG4	2091	Kpn I/EcoR V	partial V4	V4	DNA genom.
pBSE1.3	1274	EcoR I			"
pBSJ5R	1368	EcoR I/Kpn I	partial J5.1	J5-1	"
pBSC57.0	7358	Kpn I	partial J5.1/J5.2	J5-1/J5-3	"
pBS50.45	404	Kpn I	partial Ex.1 C5	Ex.1 C5	"
pBSC55.5	5750	Kpn I/EcoR V	partial Ex.1/Ex2 C5	Ex.1/Ex2 C5	"
pBSC51.6	1740	Kpn I	partial Ex.2/Ex3 C5	Ex.2/Ex3 C5	"
pBS1K	2153	Kpn I	partial Ex.3 C5	Ex.3 C5	BAC 202C5
pBS23	5401	Kpn I/EcoR V			"
pBS7	1501	EcoR V			"
pBS166	4986	EcoR V			"
PCR	2255	EcoR V			BAC 202C5
pBS21	1900	EcoR V	V2.3	V8-1	"
pBS14	1919	EcoR V			"
pBS3EX	5481	EcoR V/Xho I			"
C13F48.0	7300	Xho I/Xba I	V2.1	V2-1	BAC 634F4
pBS6X	2397	Xba I			BAC 202C5
pBS4X	2866	Xba I			"
pBS4EVX	4412	Xba I/EcoR V			"
pBS19	11231	EcoR V	V2.4	V9-1	"
PCR	796	EcoR V/EcoR I			"
pBS3	1649	EcoR I/Kpn I			DNA genom.
pBSJ3R	1153	Kpn I	partial J3.1	J3-1	"
CA3U3	3600	EcoR I	J3.1	J3-1	λ3U3
C233U3	1000	EcoR I			λ3U3
C4.3	4300	EcoR I	J3.2/Ex1 C3	J3-2/Ex1 C3	"
pBS8E	2700	EcoR I	Ex.2A/2B/3 C3	Ex.2A/2B/3 C3	λ3U1
pBS2E	3888	EcoR I			"
pBS5E	4000	EcoR I			"
pBS3E	2800	EcoR I			"
C4.4	2454	Xba I/EcoR I	V1	V1	λ6A3
C36A3	3500	Xba I	J1.2	J4-1	"
C26A3	2200	Xba I			"
C5.5	5500	Xba I	J1.1/Ex1 C1	J4-2/Ex1 C1	"
C3.0	3101	EcoR I	Ex1 C1	Ex1 C1	"
pBSC1I	1586	Kpn I/EcoR I			DNA genom.
pBSC12B	1300	EcoR I/Kpn I	partial Ex2A C1	Ex2A C1	"
pBSC13.5	3340	Kpn I	Ex2A/2B/2C/3	Ex2A/2B2C/3	"

[a] BAC clones derive from Romanov breed, λ phages derive from Altamurana breed and genomic DNA from Gentile di Puglia breed.

b)

Clone	Approximate size (bp)	Cloning sites	Gene content	Gene content (IMGT name)	Source[a]
C3-7	7000	Xba I	V5.1	V5-1	BAC201E2
C1817A52.3	2500	EcoR I	J6.1	J1-1	λ17A5
C1717A52.6	2900	EcoR I	J6.2	J1-2	"
VG5.1KJ2C21	3900	Kpn I	partial V5.1	V5-1	BAC268E12
VG5.1KJ2C5	2970	Kpn I	partial J6.1	J1-1	"
c7.5	7400	EcoR I	C6	C1	λ17A5
C517A53.8	3800	EcoR I/Not I			"
C6-12	12000	Xba I	partial C6/V5.2	C1/V5-2	BAC201E2
C5TRGJ2	3000	Kpn I			"
C14BJ2	10000	Kpn I	J2.1/J2.2	J2-1/J2-2	"
c6.0	6000	EcoR I	Ex2A/2B/2C C2	Ex2A/2B2C C2	λ12A6
c3.5	3500	EcoR I	Ex3 C2	Ex3 C2	"
c5.0	5000	EcoR I			"
C12-15	18000	Xba I	V6/J4.2	V6-1/J6-2	BAC201E2
J4.2-J4.1	3000	Kpn I			"
C8	8000	EcoR I	J4.1/Ex1 C4	J6-1/Ex1 C6	λ4P1
c0.9	900	EcoR I			"
c4.3	4300	EcoR I	Ex2A/2B/2C C4	Ex2A/2B/2C C6	λ4R1
c0.67	670	EcoR I	Ex3 C4	Ex3 C6	"
C34R13.6	3600	EcoR I			"
C124R15.0	5000	EcoR I			"

[a] BAC clones derive from Romanov breed, λ phages derive from Altamurana breed and genomic DNA from Gentile di Puglia breed.
